# Structural and Magnetization Dynamics of Borohydride-Bridged Rare-Earth Metallocenium Cations

**DOI:** 10.1021/acs.inorgchem.3c01038

**Published:** 2023-06-14

**Authors:** Christopher G. T. Price, Arpan Mondal, James P. Durrant, Jinkui Tang, Richard A. Layfield

**Affiliations:** † Department of Chemistry, School of Life Sciences, University of Sussex, Brighton BN1 9QJ, U.K.; ‡ State Key Laboratory of Rare Earth Resource Utilization, Changchun Institute of Applied Chemistry, Chinese Academy of Sciences, Changchun 130022, P. R. China

## Abstract

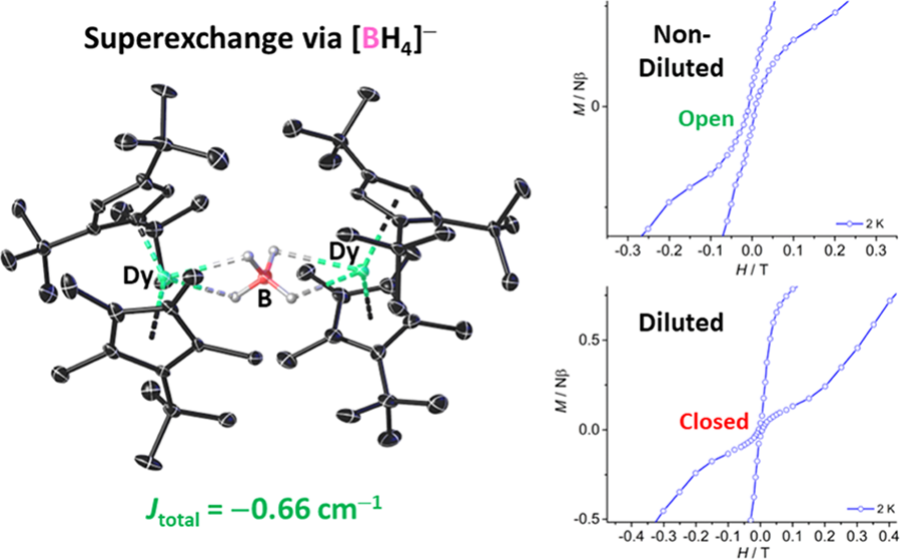

The structure and magnetic properties of the bimetallic borohydride-bridged dysprosocenium compound [{(η^5^-Cp^ttt^)(η^5^-Cp^Me4t^)Dy}_2_(μ:κ^2^:κ^2^-BH_4_)]^+^[B(C_6_F_5_)_4_]^−^ ([**3**_**Dy**_][B(C_6_F_5_)_4_]) are reported along with the solution-phase dynamics of the isostructural yttrium and lutetium analogues (Cp^ttt^ is 1,2,4-tri(*tert*-butyl)cyclopentadienyl, Cp^Me4t^ is tetramethyl(*tert*-butyl)cyclopentadienyl). The synthesis of [**3**_**M**_][B(C_6_F_5_)_4_] was accomplished in the 2:1 stoichiometric reactions of [(η^5^-Cp^ttt^)(η^5^-Cp^Me4t^)Dy(BH_4_)] (**2**_**M**_) with [CPh_3_][B(C_6_F_5_)_4_], with the metallocenes **2**_**M**_ obtained from reactions of the half-sandwich complexes [(η^5^-Cp^ttt^)M(BH_4_)_2_(THF)] (**1**_**M**_) (M = Y, Dy, Lu) with NaCp^Me4t^. Crystallographic studies show significant lengthening of the M···B distance on moving through the series **1**_**M**_, **2**_**M**_, and **3**_**M**_, with essentially linear {M···B···M} bridges in **3**_**M**_. Multinuclear NMR spectroscopy indicates restricted rotation of the Cp^ttt^ ligands in **3**_**Y**_ and **3**_**Lu**_ in solution. The single-molecule magnet (SMM) properties of [**3**_**M**_][B(C_6_F_5_)_4_] are characterized by Raman and Orbach processes, with an effective barrier of 533(18) cm^–1^ and relaxation via the second-excited Kramers doublet. Although quantum tunneling of the magnetization (QTM) was not observed for [**3**_**M**_][B(C_6_F_5_)_4_], it was, surprisingly, found in its magnetically dilute version, which has a very similar barrier of *U*_eff_ = 499(21) cm^–1^. Consistent with this observation, slightly wider openings of the magnetic hysteresis loop at 2 K are found for [**3**_**M**_][B(C_6_F_5_)_4_] but not for the diluted analogue. The dynamic magnetic properties of the dysprosium SMMs and the role of exchange interactions in **3**_**Dy**_ are interpreted with the aid of multireference ab initio calculations.

## Abstract 2

The structure and dynamic magnetic properties of a bimetallic borohydride-bridged dysprosocenium single-molecule magnet are reported and interpreted with the aid of ab initio calculations. The magnetic relaxation in this compound is characterized by Raman and Orbach processes, the latter with a barrier of 533(18) cm^−1^. Magnetic dilution introduces QTM as the dominant relaxation process at low temperatures. The solution-phase dynamics of the isostructural yttrium and lutetium analogues are also described.

## Introduction

The discovery of slow magnetic relaxation and magnetic hysteresis in dysprosium metallocenes has led to the development of a large and growing family of organometallic lanthanide single-molecule magnets (SMMs), typically based on terbium, dysprosium, or erbium.^1−3^ Different types of SMM within this family include: weakly exchange-coupled bimetallic and trimetallic metallocenes with μ-bridging group 14, 15, or 16 donors;^4−9^ strongly exchange-coupled, radical-bridged metallocenes;^10−13^ pseudo-2-coordinate metallocenium cations of the type [(η^5^-Cp^R^)_2_Dy]^+^ (Cp^R^ is a sterically bulky cyclopentadienyl ligand);^14−17^ metallocene-type complexes containing heterocyclopentadienyl ligands, such as borolyl,^18^ phospholyl,^19,20^ or cyclobutadienyl;^21,22^ and linear divalent dysprosium and terbium metallocenes.^23^ A half-sandwich dysprosium SMM containing a direct metal–metal bonding interaction has recently been described,^24^ and several erbium sandwich SMMs based on the {Er(η^8^-COT)}^+^ (COT = cyclo-octatetraenyl) building block are also known.^25−34^ Some members of the family show extremely high effective energy barriers to reversal of the magnetization (*U*_eff_)^18^ and, in some cases, magnetic hysteresis and coercivity above liquid nitrogen temperatures.^14^ Notwithstanding their air sensitivity, discoveries such as these fuel the optimistic view that SMMs may eventually find use in functional quantum materials.^35−37^

Beyond record-breaking performance parameters and potential applications, perhaps the most valuable contribution arising from studies of metallocene SMMs is the new fundamental insight they provide into lanthanide electronic structure and magnetism, complementing the vast body of knowledge generated from studies of lanthanide SMMs based on Werner-type coordination chemistry.^38−42^ This is particularly true in the case of crystal field and magnetic exchange interactions involving highly anisotropic lanthanide ions and the softer, more polarizable ligands that become accessible through organometallic synthetic approaches. In this context, our attention has turned to borohydride as a μ-bridging ligand in dysprosium metallocene SMMs. The multiple coordination modes available to the [BH_4_]^−^ anion make this ligand a source of structural diversity, which can, for example, support the formation of bimetallic and trimetallic borohydride-bridged dysprosium SMMs.^43,44^ Furthermore, studies of the magnetic susceptibility of binary rare-earth borohydrides, i.e., [M(BH_4_)_3_], revealed that the boron 2p orbitals play an important role in the magnetic exchange between lanthanide ions, which can be weakly ferromagnetic or antiferromagnetic.^45^ Weak ferromagnetic exchange was reported for the α- and β-forms of [Dy(BH_4_)_3_], which may be relevant to the design of borohydride-ligated dysprosium SMMs. To provide further insight into the structures and magnetic properties of borohydride-bridged metallocenium cations, we now report the synthesis of the bimetallic complexes [{(η^5^-Cp^ttt^)(η^5^-Cp^Me4t^)M}_2_(μ:κ^2^:κ^2^-BH_4_)]^+^ as salts of [B(C_6_F_5_)_4_]^−^ ([**3**_**M**_][B(C_6_F_5_)_4_]), where M is yttrium, dysprosium, and lutetium; Cp^ttt^ is 1,2,4-tri(*tert*-butyl)cyclopentadienyl; and Cp^Me4t^ is tetramethyl(*tert*-butyl)cyclopentadienyl.

## Results and Discussion

**Scheme 1:**
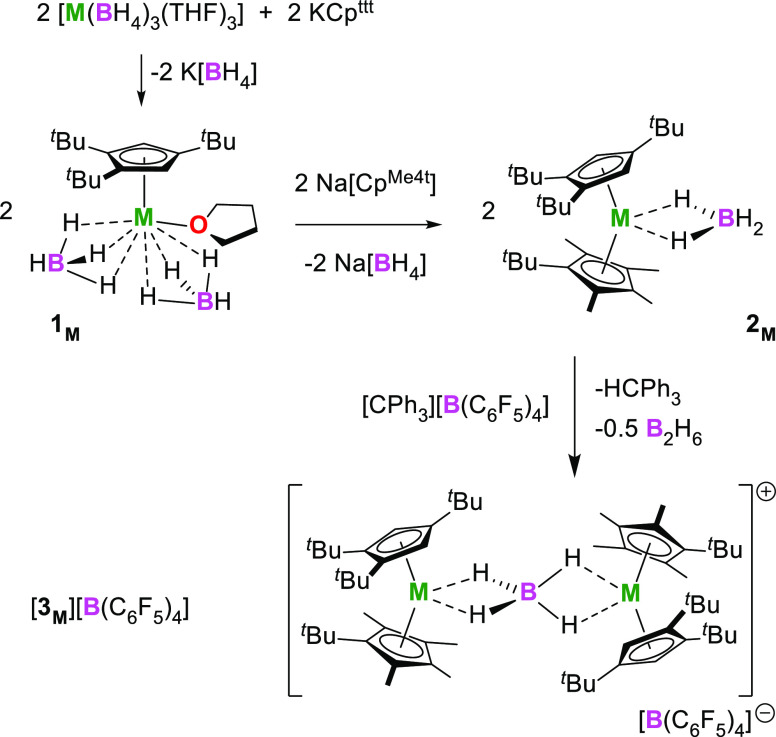
Synthesis of **1**_**M**_, **2**_**M**_, and [**3**_**M**_][B(C_6_F_5_)_4_] (M = Y, Dy, Lu)

The synthesis of [**3**_**M**_][B(C_6_F_5_)_4_] was accomplished by first reacting [M(BH_4_)_3_(THF)_3_] with KCp^ttt^ to produce [(η^5^-Cp^ttt^)M(BH_4_)_2_(THF)] (**1**_**M**_) (M = Dy, Y, Lu in Scheme 1). Compounds **1**_**M**_ were then isolated and reacted with NaCp^Me4t^ to give the heteroleptic metallocenes [(η^5^-Cp^ttt^)Dy(η^5^-Cp^Me4t^)(BH_4_)] (**2**_**M**_). With the aim of removing the borohydride ligand from **2**_**M**_, the metallocenes were added to a stirred suspension of one stoichiometric equivalent of the electrophilic reagent [CPh_3_][B(C_6_F_5_)_4_] in hexane. Subsequent workup and analysis of the products using X-ray crystallography revealed the formation of the ion-separated borohydride-bridged bimetallic species [**3**_**Y**_][B(C_6_F_5_)_4_], [**3**_**Dy**_][B(C_6_F_5_)_4_], and [**3**_**Lu**_][B(C_6_F_5_)_4_]. Thus, only half of the **2**_**M**_ reacts with [CPh_3_][B(C_6_F_5_)_4_], suggesting that the putative cations [(η^5^-Cp^ttt^)M(η^5^-Cp^Me4t^)]^+^ are trapped upon formation by unreacted **2**_**M**_. Addition of excess [CPh_3_][B(C_6_F_5_)_4_] either to **2**_**M**_ or [**3**_**M**_][B(C_6_F_5_)_4_] did not change the outcome. The rational synthesis of [**3**_**M**_][B(C_6_F_5_)_4_] was then achieved by combining **2**_**M**_ and [CPh_3_][B(C_6_F_5_)_4_] in a 2:1 stoichiometry, resulting in isolated yields of 81, 76, and 31% based on yttrium, dysprosium, and lutetium, respectively.

**Figure 1:**
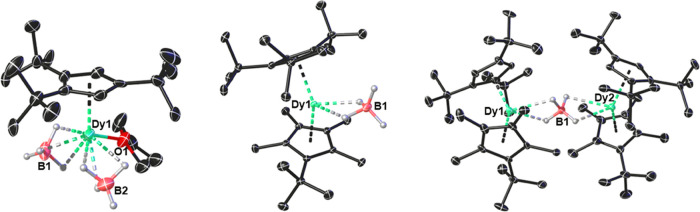
Thermal ellipsoid representation (50% probability) of the molecular structures of **1**_**Dy**_ (disordered component A, left), **2**_**Dy**_ (center), and [**3**_**Dy**_][B(C_6_F_5_)_4_] (right). Carbon atoms are unlabeled and depicted in black. Hydrogen atoms, except those of the borohydride ligands, are omitted for clarity.

Compounds **1**_**M**_ are isostructural, as are compounds **2**_**M**_ and compounds [**3**_**M**_][B(C_6_F_5_)_4_]; hence, only the solid-state molecular structures of the dysprosium versions are described in detail here (Figure 1 and Tables S1–S6), whereas the yttrium and lutetium versions are summarized in the Supporting Information (Figures S1–S3 and Tables S1–S6).

The Cp^ttt^ ligand in the piano-stool complexes **1**_**M**_ is disordered over two sites with approximately 50:50 occupancy (referred to as parts A and B). The Dy–C distances in **1**_**Dy**_ are in the range 2.598(10)–2.720(12) and 2.562(10)–2.706(12) Å for parts A and B, respectively, and the Dy–Cp_cent_^ttt^ distances are 2.369(6) and 2.357(6) Å, respectively (“cent” refers to the ligand centroid). The borohydride ligands in **1**_**M**_ adopt the κ^3^-bonding mode, leading to Dy···B1 and Dy···B2 separations of 2.507(6) and 2.504(5) Å, respectively, with the *H*-donor atoms occupying axial and equatorial positions relative to an axial direction defined by the Cp^ttt^ ligand. Consistent with the κ^3^-borohydride motif, two sets of absorptions occur in the FTIR spectra of **1**_**M**_, centered on 2470 and 2400–2050 cm^–1^ for the terminal and bridging B–H stretching vibrations, respectively (Figure S4).

In the metallocenes **2**_**M**_, the M–C distances span similar ranges to those in **1**_**M**_, being 2.626(3)–2.709(3) and 2.646(3)–2.655(3) Å for the Cp^ttt^ and Cp^Me4t^ ligands in **2**_**Dy**_, respectively, corresponding to Dy–Cp_cent_^ttt^ and Dy–Cp_cent_^Me4t^ distances of 2.3692(11) and 2.3578(12) Å, respectively. The Cp_cent_^ttt^–Dy–Cp_cent_^Me4t^ angle is 143.44(4)°. To accommodate the Cp^Me4t^ ligand, the remaining borohydride moves away from the metal, resulting in a significant increase in the Dy···B separation to 2.713(3) in **2**_**Dy**_ and a switch to the κ^2^-coordination mode in the equatorial plane, relative to the axial direction defined by the cyclopentadienyl ligands. The FTIR spectrum of **2**_**Dy**_ shows stretching absorptions in the range 2450–2250 cm^–1^ for the terminal B–H bonds and in the range 2250–1900 cm^–1^ for the bridging B–H bonds (Figure S5).

The structures of [{(η^5^-Cp^ttt^)(η^5^-Cp^Me4t^)M}_2_(μ:κ^2^:κ^2^-BH_4_)]^+^ (**3**_**M**_) consist of two metallocene units twisted with respect to each other because of the μ:κ^2^:κ^2^-bridging mode of the tetrahedral borohydride ligand. The twist can be quantified by the dihedral angle formed by the two Cp_cent_^ttt^–M–Cp_cent_^Me4t^ planes, with a slight increase from 67.95(5)° in **3**_**Y**_ to 68.70(10)° in **3**_**Dy**_ and 70.65(10)° in **3**_**Lu**_ presumably a consequence of the different radii of the M^3+^ ions and steric congestion between the substituents. Similarly, the metallocene units are slightly more bent in **3**_**M**_ than in the corresponding precursor complexes **2**_**M**_, as typified by the Cp_cent_^ttt^–Dy–Cp_cent_^Me4t^ angles in **3**_**Dy**_ of 138.94(11)° for Dy1 and 141.23(12)° for Dy2. Significant lengthening of the M···B distances also occurs in **3**_**M**_ relative to **2**_**M**_, with Dy1···B1 and Dy2···B1 separations of 2.855(7) and 2.857(7) Å in **3**_**Dy**_, resulting in a Dy1···Dy2 distance of 5.7097(6) Å and a near-linear Dy–B–Dy angle of 176.5(3)°. For comparison, the Y1···B1 and Y2···B1 separations in **3**_**Y**_ are 2.853(4) and 2.839(4) Å, respectively, and the Lu1···B1 and Lu2···B1 separations in **3**_**Lu**_ are 2.801(9) and 2.808(9) Å, respectively, reflecting the smaller ionic radius of Lu^3+^. The coordination mode of the borohydride ligand in **3**_**M**_ results in a single FTIR absorption for the B–H stretching vibrations, centered on 2265 cm^–1^ for all three dimetallic complexes (Figure S6).

The ^1^H, ^11^B, and ^13^C NMR spectra of diamagnetic **1**_**Y**_ (Figures S7–S11), **1**_**Lu**_ (Figures S12–S16), **2**_**Y**_ (Figures S17–S21), and **2**_**Lu**_ (Figures S22–S26) are straightforward to interpret and show that the structures in solution are consistent with the solid state. In contrast, the ^11^B{^1^H} and ^11^B NMR spectra of [**3**_**Y**_][B(C_6_F_5_)_4_] (Figures S31, S32, S48, and S49) and [**3**_**Lu**_][B(C_6_F_5_)_4_] (Figures S54, S55, S71, and S72) in chlorobenzene-D_5_ show three environments and a marked temperature dependence from −30 to +80 °C. One ^11^B resonance observed for both compounds is essentially temperature-independent and readily assignable to the [B(C_6_F_5_)_4_]^−^ anion at δ(^11^B) = −15.55 ppm. The other two resonances occur at higher field and are distinct from −30 °C up to +50 °C for **3**_**Y**_ and up to +20 °C for **3**_**Lu**_, before coalescing at higher temperatures. Focusing on the ^11^B{^1^H} NMR spectrum of **3**_**Y**_ at +10 °C (Figure S49), the more intense resonance at δ(^11^B) = −16.84 ppm appears as a broad binomial triplet, presumably due to coupling with the ^89^Y nuclei (*I* = 1/2, 100%) with ^2^*J* ≈ 7 Hz. The minor resonance centered on δ(^11^B) = −17.27 ppm at +10 °C also appears to have a triplet structure, with an estimated coupling constant of ^2^*J* ≈ 5 Hz. The analogous resonances in the ^11^B{^1^H} NMR spectrum of **3**_**Lu**_ at 0 °C occur at δ(^11^B) = −17.17 ppm and δ(^11^B) = −17.56 ppm as singlets (Figure S72, coupling to strongly quadrupolar ^175^Lu is not normally observed), indicating that **3**_**Y**_ and **3**_**Lu**_ behave similarly in solution. In the ^1^H-coupled ^11^B NMR spectra, the major resonances observed in the ^11^B{^1^H} NMR spectra appear as binomial quintets, with the minor resonances occurring as shoulder-type peaks, confirming that both are due to the borohydride ligand (Figures S48 and S71). Coalescence of the resonances at higher temperatures suggests that the two environments interchange rapidly on the NMR timescale and that this process is more facile for **3**_**Lu**_ than **3**_**Y**_.

**Figure 2:**
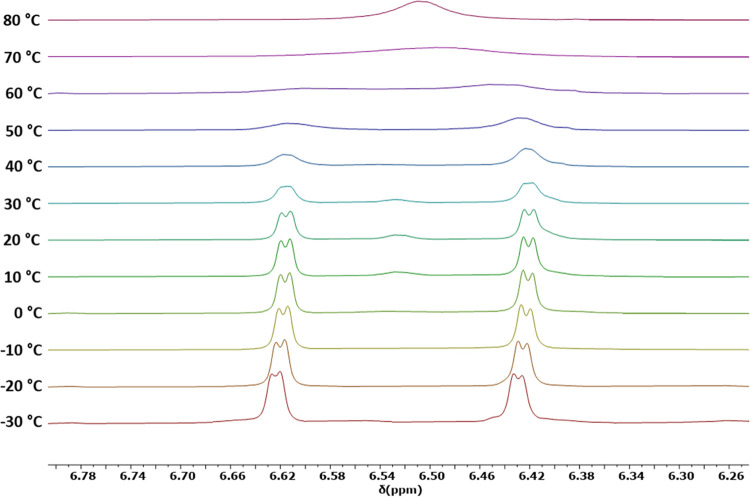
^1^H NMR spectra of [**3**_**Y**_][B(C_6_F_5_)_4_] in the range δ = 6.2–6.8 ppm, corresponding to the methine environments on the Cp^ttt^ ligands, from −30 to 80 °C in chlorobenzene-D_5_.

The solution dynamics of [**3**_**Y**_][B(C_6_F_5_)_4_] and [**3**_**Lu**_][B(C_6_F_5_)_4_] were elucidated further via their ^1^H NMR spectra (Figures S27, S28, S33–S47 and Figures S50, S51, S56–S70, respectively), illustrated most clearly with the resonances of the Cp^ttt^ ring protons (Figures 2 and S70). For **3**_**Y**_, from −30 to +20 °C, two mutually coupled 1:1 doublets occur at δ = 6.43 and 6.62 ppm with ^4^*J* = 4.0 Hz. At higher temperatures, the signals broaden and eventually coalesce on δ = 6.51 ppm at +80 °C. The behavior of the Cp^ttt^ ring protons in **3**_**Lu**_ is very similar, with two mutually coupled 1:1 doublets at 6.47 and 6.74 ppm at −30 °C, with ^4^*J* = 4.0 Hz (Figure S70). The doublets are distinct up to +40 °C before merging at higher temperatures and coalescing as a singlet at +80 °C with δ = 6.58 ppm. Resonances for the *tert*-butyl and methyl ligand substituents in **3**_**Y**_ and **3**_**Lu**_ occur in a more congested region of the spectrum. At +80 °C, the ^1^H NMR spectrum of **3**_**Lu**_ shows five signals expected for the Cp^ttt^ and Cp^Me4t^ ligands; two sharp signals occur for the Cp^ttt^*tert*-butyl groups at 1.18 and 1.20 ppm, and one for the Cp^Me4t^*tert*-butyl group at 1.41 ppm. The Cp^Me4t^ methyl groups occur at 2.07 ppm (broad with FWHM = 20.8 Hz) and 2.39 ppm. On lowering the temperature, significant broadening of the resonances due to the Cp^Me4t^ substituents occurs followed by decoalescence into multiple overlapping singlets at −30 °C. Consistent with the same dynamic process being more facile in the lutetium complex than in the yttrium analogue, the ^1^H NMR spectrum of [**3**_**Y**_][B(C_6_F_5_)_4_] at +80 °C is reminiscent of the spectrum of [**3**_**Lu**_][B(C_6_F_5_)_4_] at +60 °C, with a very similar decoalescence pattern observed at lower temperatures (Figures S67–S70).

An Eyring analysis of the ^1^H NMR resonances associated with the Cp^ttt^ methine protons yielded estimated Δ*G*^‡^ values of 67 kJ mol^–1^ for [**3**_**Y**_][B(C_6_F_5_)_4_] at 333 K and 62 kJ mol^–1^ for [**3**_**Lu**_][B(C_6_F_5_)_4_] at 313 K (Figure S73, Table S7). The solution-phase behavior of **3**_**M**_ is evidently complex and indicative of restricted rotation of the cyclopentadienyl ligands, presumably because of steric interactions between substituents on the two metallocene units, consistent with the static solid-state molecular structures. The estimated barriers for **3**_**M**_ are comparable to those determined for the samarium(II) metallocenes [(η^5^-Cp^ttt^)_2_Sm(L)] (L = THF, pyridine), for which restricted rotation of the Cp^ttt^ ligands was also proposed.^46^

## Magnetic Properties

The molar magnetic susceptibility (χ_M_) of **1**_**Dy**_, **2**_**Dy**_, and [**3**_**Dy**_][B(C_6_F_5_)_4_] was determined in static (DC) and dynamic (AC) magnetic fields along with the isothermal magnetization (*M*) and magnetic hysteresis properties. In an applied field of 0.1 T (1 kOe), the values of χ_M_*T* at 300 K are 14.01, 13.52, and 29.43 cm^3^ K mol^–1^ for **1**_**Dy**_, **2**_**Dy**_, and [**3**_**Dy**_][B(C_6_F_5_)_4_], respectively, consistent with the expected values for one (14.1 cm^3^ K mol^–1^) or two (28.2 cm^3^ K mol^–1^) Dy^3+^ ions (Figures S74, S77, and S79).^47^ For **1**_**Dy**_, χ_M_*T* shows a weak temperature dependence in the region 10–300 K before decreasing slightly at lower temperatures, reaching a value of 12.09 cm^3^ K mol^–1^ at 2 K. Similar behavior is found for **2**_**Dy**_, with χ_M_*T* reaching 10.90 cm^3^ K mol^–1^ at 2 K. The gradual decrease in χ_M_*T* for [**3**_**Dy**_][B(C_6_F_5_)_4_] is followed by a more pronounced decrease below 10 K, reaching 19.25 cm^3^ K mol^–1^ at 2 K. The temperature dependence of the magnetic susceptibility for **1**_**Dy**_ and **2**_**Dy**_ is most likely a consequence of thermal depopulation of the excited crystal field levels with the ^6^H_15/2_ ground multiplet of Dy^3+^, whereas in the case of [**3**_**Dy**_][B(C_6_F_5_)_4_], the greater decrease in χ_M_*T* at low temperatures is also consistent with the occurrence of magnetic blocking (see below). The isothermal field dependence of the magnetization, *M*(*H*), for **1**_**Dy**_, **2**_**Dy**_, and [**3**_**Dy**_][B(C_6_F_5_)_4_] reaches values of 5.37, 4.96, and 9.64 Nβ, respectively, at a field of 7 T and a temperature of 2 K (Figures S74, S77, and S79).

**Figure 3:**
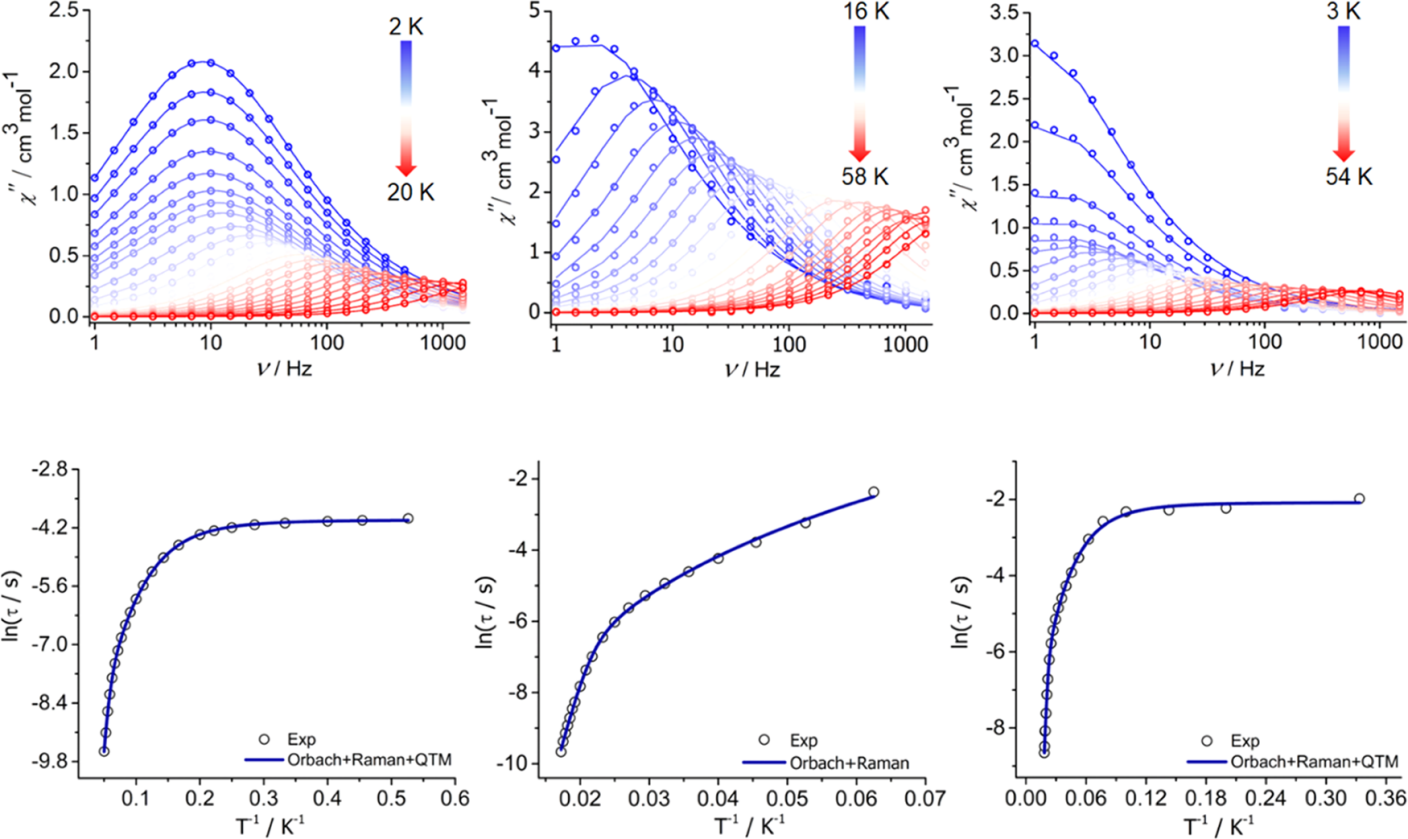
Top: Imaginary part of the AC susceptibility as a function of frequency, χ″(*v*), in zero DC field for: **1**_**Dy**_ at *T* = 2–20 K (left); [**3**_**Dy**_][B(C_6_F_5_)_4_] at *T* = 16–58 K (center); and **Dy@3**_**Y**_ at *T* = 3–54 K (right). Bottom: Plots of ln τ versus *T*^–1^ for **1**_**Dy**_ (left), [**3**_**Dy**_][B(C_6_F_5_**)**_4_] (middle), and **Dy@3**_**Y**_ (right), with solid lines representing the best fits to the data using the parameters stated in the text.

AC magnetic susceptibility studies revealed that **1**_**Dy**_ and [**3**_**Dy**_][B(C_6_F_5_)_4_] are SMMs in zero DC field, whereas **2**_**Dy**_ is not. The frequency dependence of the imaginary part of the AC susceptibility, χ″(*ν*), for **1**_**Dy**_ shows maxima in the temperature range 1.9–20 K, with a weak temperature dependence of the frequency maximum up to 4.5 K and a more pronounced temperature dependence up to 20 K (Figures 3 and S75). Cole–Cole plots of the imaginary versus real parts of the susceptibility, χ″(χ′), at each temperature adopt a parabolic shape and were curve-fitted using α-parameters in the range 0.04–0.32 (Figure S76, Table S8). These data indicate a relatively large distribution of relaxation times (τ), spanning 18.77 ms at 1.9 K to 0.07 ms at 20 K. The plot of ln τ versus *T*^–1^ for **1**_**Dy**_ revealed a linear dependence of the relaxation time in the region 16–20 K, an essentially temperature-independent regime below 5 K, and a region of curvature at intermediate temperatures (Figure 3). Qualitatively, these data indicate Orbach and Raman relaxation processes at higher temperatures and quantum tunneling of the magnetization (QTM) at lower temperatures. A fit of the relaxation times was achieved using τ^–1^ = τ_0_^–1^ e^–*U*_eff_/*k*_B_*T*^ + *CT*^*n*^ + τ_QTM_^–1^, in which τ_0_^–1^ is the attempt time; *C* and *n* are the Raman coefficient and Raman exponent, respectively; and τ_QTM_^–1^ is the rate of QTM. The resulting fit parameters for **1**_**Dy**_ are τ_0_ = 7.02 × 10^–10^ s, *U*_eff_ = 165(5) cm^–1^, *C* = 0.051(4) × 10^–3^ s^–1^ K^–*n*^, *n* = 3.78(3), and τ_QTM_ = 18.0(2) ms.

AC susceptibility measurements on **2**_**Dy**_ in zero DC field did not produce maxima in χ″(*T*) even at the maximum frequency of 1000 Hz accessible to the measurement system (Figure S78). In contrast, [**3**_**Dy**_][B(C_6_F_5_)_4_] showed χ″(*v*) maxima in the range 16–58 K, with the position of the peak maximum shifting to a higher frequency with increasing temperature (Figures 3 and S80). The Cole–Cole plots for the borohydride-bridged bimetallic species were curve-fitted using α = 0.04–0.32 (Figure S81, Table S9). Unlike **1**_**Dy**_, the ln τ versus *T*^–1^ plot for [**3**_**Dy**_][B(C_6_F_5_)_4_] does not enter a temperature-independent regime and consists of a linear region in the range 50–58 K and a curved dependence at lower temperatures (Figure 3). A fit of the relaxation times using only Orbach and Raman parameters, i.e., τ^–1^ = τ_0_^–1^ e^–*U*_eff_/*k*_B_*T*^ + *CT*^*n*^, was achieved with τ_0_ = 1.32 × 10^–10^ s, *U*_eff_ = 533(18) cm^–1^, *C* = 3.7(8) × 10^–4^ s^–1^ K^–*n*^ and *n* = 3.74(6).

To gain insight into the role of magnetic exchange interactions in determining the SMM properties of [**3**_**Dy**_][B(C_6_F_5_)_4_], we conducted a dilution study using the heterobimetallic species [(η^5^-Cp^ttt^)(η^5^-Cp^Me4t^)Dy(μ:κ^2^:κ^2^-BH_4_)Y(η^5^-Cp^ttt^)(η^5^-Cp^Me4t^)][B(C_6_F_5_)_4_], which was synthesized in a diamagnetic matrix of [**3**_**Y**_][B(C_6_F_5_)_4_] (**Dy@3**_**Y**_). A 16% dilution level of dysprosium at the single-ion level was achieved by reacting an appropriate mixture of **2**_**Dy**_ and **2**_**Y**_ with [CPh_3_][B(C_6_F_5_)_4_] (see the Supporting Information for details), producing a sample containing approximately 27% of the heterobimetallic complex. The AC susceptibility properties of **Dy@3**_**Y**_ are somewhat different from those of the dysprosium-only version, with the observation of QTM in zero field at low temperatures. While maxima were observed in χ″(*v*) in the range 3–54 K, they are essentially temperature-independent up to 10 K before shifting to higher frequencies with increasing temperature (Figures 3, S82, and S83). Fitting the temperature dependence of the relaxation time for **Dy@3**_**Y**_ using Orbach, Raman, and QTM terms yielded τ_0_ = 5.80 × 10^–10^ s, *U*_eff_ = 499(21) cm^–1^, *C* = 0.0014(5) s^–1^ K^–*n*^, *n* = 3.3(1), and τ_QTM_ = 125(6) ms (Figure 3). Thus, the similar Orbach and Raman parameters for [**3**_**Dy**_][B(C_6_F_5_)_4_] and **Dy@3**_**Y**_ indicate similar high-temperature dynamic magnetism. In contrast, the behavior of **Dy@3**_**Y**_ is characterized by QTM at low temperatures, whereas the relaxation times for [**3**_**Dy**_][B(C_6_F_5_)_4_] do not enter a temperature-independent regime within the lower temperature limits of our measurement system. These observations contrast with the behavior of related dimetallic dysprosium SMMs,^48,49^ including [{(η^5^-Cp*)_2_Dy}(μ-Fp)]_2_ (Fp = CpFe(CO)_2_), where dilution results in a reduction in the rate of QTM and an increase in relaxation times at low temperatures.^50^ The differing behavior of [**3**_**Dy**_][B(C_6_F_5_)_4_] and **Dy@3**_**Y**_ is explained below with the aid of an ab initio theoretical study.

A qualitative rationale for the variation in SMM behavior for **1**_**Dy**_, **2**_**Dy**_, and [**3**_**Dy**_][B(C_6_F_5_)_4_] can be derived by considering the interaction of the oblate spheroidal 4f^9^ electron density of Dy^3+^ with the crystal field. For the piano-stool complex **1**_**Dy**_, the crystal-field splitting should be dominated by the axial Cp^ttt^ ligand due to its proximity to dysprosium, while also consisting of axial and equatorial contributions from the hydride donors of the κ^3^-borohydride ligands as well as a near-equatorial THF ligand. This should lead to SMM behavior in zero field in addition to waist-restricted *M*(*H*) hysteresis loops (see below), consistent with our observations. A similar scenario was observed for the isostructural SMM [(η^5^-C_5_^*i*^Pr_5_)M(BH_4_)_2_(THF)], which has a larger energy barrier of *U*_eff_ = 241(7) cm^–1^.^14^ The only significant difference in the structures of **1**_**Dy**_ and [(η^5^-C_5_^*i*^Pr_5_)Dy(BH_4_)_2_(THF)] is the Dy–O distance to the THF ligand, which is 0.074 Å shorter in **1**_**Dy**_, leading to a stronger equatorial crystal field and, hence, a smaller barrier in **1**_**Dy**_. This observation highlights the sensitivity of the SMM properties to local coordination geometry, particularly in relation to the bulk of the cyclopentadienyl substituents and how this affects coordination of the other ligands. The dynamic magnetic properties of **2**_**Dy**_ reflect the appreciable bending angle of 143.44(4)° and coordination of an equatorial κ^2^-borohydride ligand, which should result in diminished magnetic axiality. Comparison with the related metallocene [(η^5^-C_5_^*i*^Pr_5_)Dy(η^5^-Cp*)(κ^2^-BH_4_)] is instructive; this species has a slightly less bent structure with an angle of 150.289(1)°, leading to a very small *U*_eff_ of 7(1) cm^–1^.^13^ The absence of a measurable barrier in **2**_**Dy**_ is, therefore, likely to be a consequence of the greater bending that occurs with the less bulky cyclopentadienyl substituents. In the case of the bimetallic cation **3**_**Dy**_, while the two metallocene units retain an appreciable degree of bending with angles of 138.94(11) and 141.23(12)°, the Dy–Cp_cent_ distances are shorter than in **2**_**Dy**_, leading to a stronger axial crystal field. Furthermore, the distance to the μ:κ^2^:κ^2^-borohydride ligand in **3**_**Dy**_, as measured by the Dy···B separation, is considerably longer than in **2**_**Dy**_ by 0.14 Å, meaning a much weaker equatorial crystal field in the bimetallic SMM and a more dominant axial crystal field originating from the cyclopentadienyl ligands.

**Figure 4:**
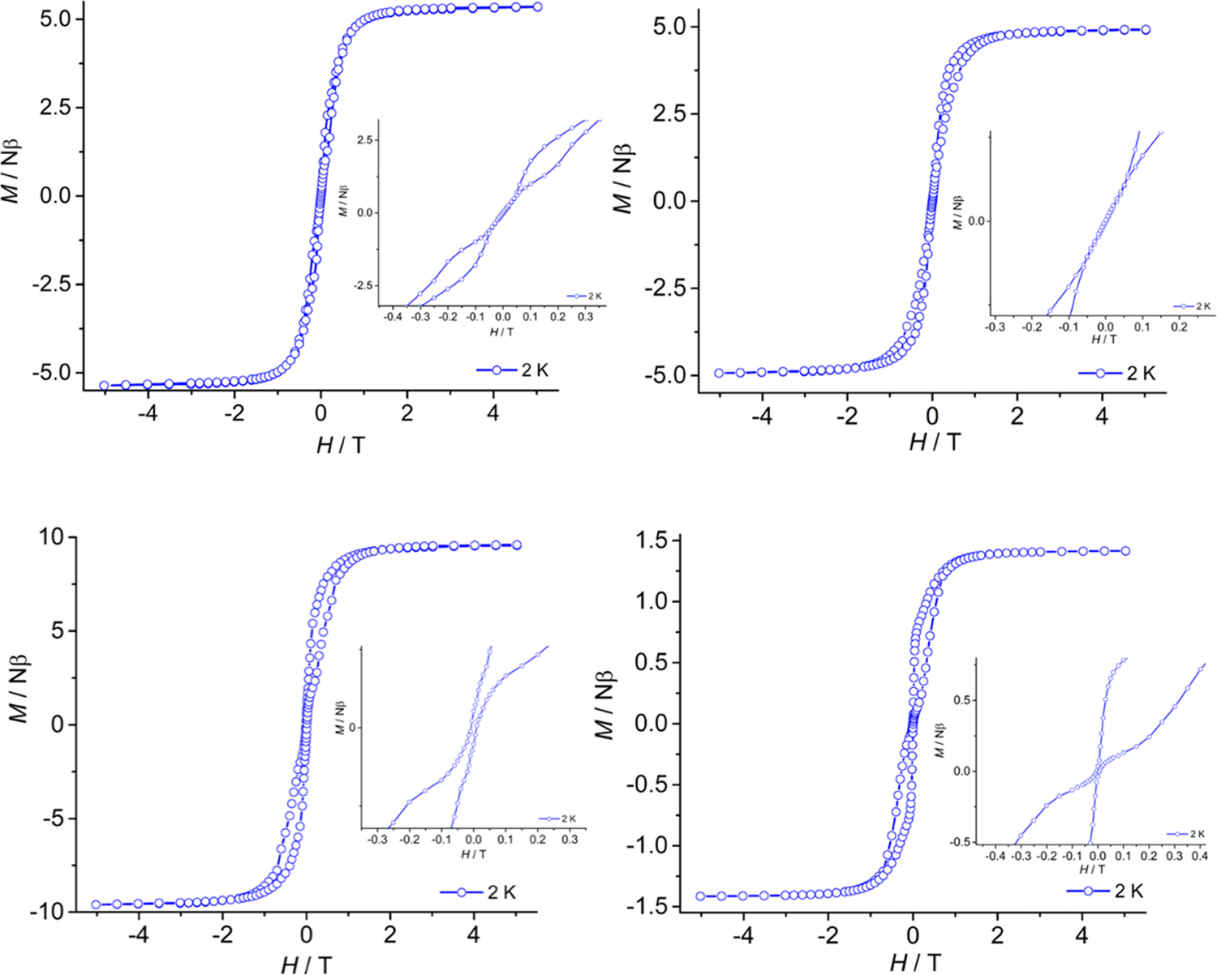
Magnetic hysteresis loop for **1**_**Dy**_ (top left), **2**_**Dy**_ (top right), [**3**_**Dy**_][B(C_6_F_5_)_4_] (bottom left), and **Dy@3**_**Y**_ (bottom right). The data were continuously collected at 2 K under a varying field sweep rate (5 mT s^–1^ |0–0.02| T, 10 mT s^–1^ |0.02–0.1| T, 50 mT s^–1^ |0.1–0.4| T, 100 mT s^–1^ |0.4–2.0| T, 200 mT s^–1^ |2.0–3.0| T, 500 mT s^–1^ |3.0–5.0| T). Solid lines are a guide to the eye.

The magnetic hysteresis properties of each dysprosium complex were measured at 2 K with field sweep rates varying in the range of 5–500 mT s^–1^ (Figures 4 and S84). The *M*(*H*) hysteresis data for **1**_**Dy**_ and **2**_**Dy**_ consist of S-shaped loops that close at zero field, but open slightly in fields greater than approximately 0.1 T. These observations indicate fast QTM in zero applied field, broadly consistent with the temperature dependence of the relaxation time determined through AC susceptibility measurements. The hysteresis loops for [**3**_**Dy**_][B(C_6_F_5_)_4_] are also S-shaped; however, the openings are typically wider than in **1**_**Dy**_ and **2**_**Dy**_ and they also remain open at zero field, implying that QTM is not a significant relaxation pathway in this system, as found with the AC susceptibility measurements. The hysteresis loops for **Dy@3**_**Y**_ are slightly wider than those for [**3**_**Dy**_][B(C_6_F_5_)_4_] in the region around *H* = 0.5 T, but closed at zero field owing to QTM. While the observation of QTM in the magnetic hysteresis of **Dy@3**_**Y**_ is consistent with the AC susceptibility data, the fact that QTM is prominent in the magnetically dilute sample but not in the dysprosium-only SMM [**3**_**Dy**_][B(C_6_F_5_)_4_] is counterintuitive. Principally, this is because intramolecular exchange interactions between Dy^3+^ ions are often responsible for fast QTM, which can be mitigated by magnetic dilution in an isostructural diamagnetic host. This has been demonstrated in several examples of dysprosium metallocene SMMs. To shed light on this unusual behavior, we undertook a theoretical study of the dysprosium centers in **1**_**Dy**_, **2**_**Dy**_, and **3**_**Dy**_.

## Theoretical Studies

The electronic structure and magnetization dynamics of **1**_**Dy**_, **2**_**Dy**_, and **3**_**Dy**_ were analyzed using ab initio calculations implemented within the ORCA 5.0.2 software.^51^ All calculations were of the CASSCF/QDPT/SINGLE_ANISO type and were performed using the atomic coordinates of all nonhydrogen atoms as determined by X-ray crystallography. Hydrogen atoms were placed in optimized positions. For **1**_**Dy**_, disordered parts A and B were calculated, and for **3**_**Dy**_ calculations were performed on the two unique dysprosium centers.

**Figure 5:**
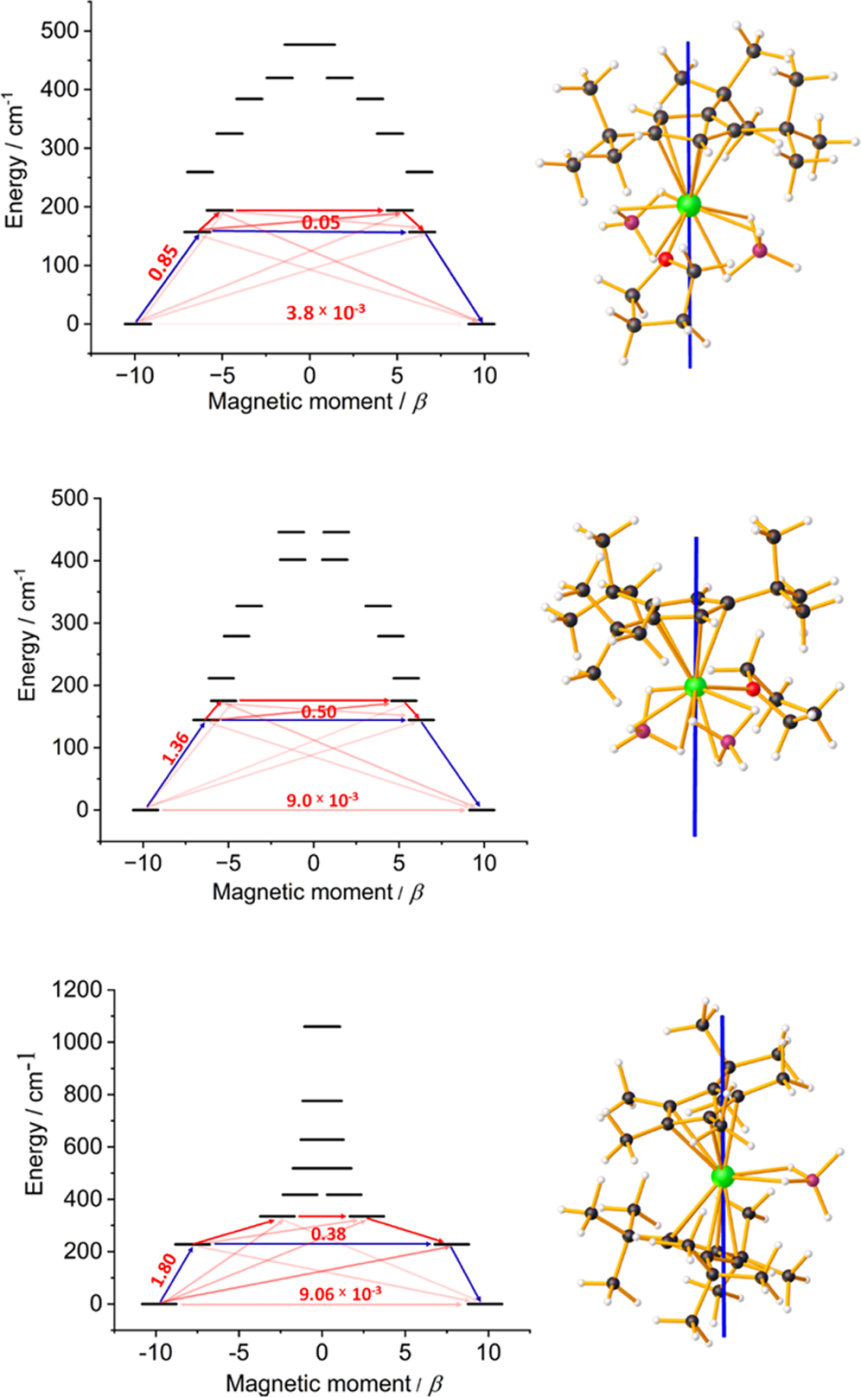
Relaxation barriers and principal magnetic axes (blue lines) of the ground KD for **1**_**Dy**_ (disordered part A, top), **1**_**Dy**_ (disordered part B, center), and **2**_**Dy**_ (bottom). Arrows on the relaxation barriers represent possible relaxation processes, with associated numbers being the transition magnetic moments. Darker red shading represents transitions of higher probability, with blue arrows being the most probable pathway according to ab initio calculations. Green = Dy, pink = B, black = C, red = O, gray = H.

The eight Kramers doublets (KDs) arising from splitting of the ^6^H_15/2_ ground multiplet by the crystal field reach energies of up to 445 and 477 cm^–1^ for the eighth KD in parts A and B of **1**_**Dy**_, respectively, up to 1060 cm^–1^ in **2**_**Dy**_, and up to 1162 and 1205 cm^–1^ for Dy1 and Dy2, respectively, in **3**_**Dy**_ (Table 1). The trend of increasing splitting across the series reflects the expected pattern based on the number of axial cyclopentadienyl ligands in each complex and their relative Dy–Cp distances. The calculated *g*-tensors and wavefunction compositions of each KD are shown in Tables S11–S15. The *g*-tensors show that both ground KDs in disordered **1**_**Dy**_ are highly axial, with particularly small transverse contributions for part A (*g*_*x*_ = 0.006, *g*_*y*_ = 0.016, *g*_*z*_ = 19.70) and somewhat larger transverse components for part B (*g*_*x*_ = 0.018, *g*_*y*_ = 0.035, *g*_*z*_ = 19.62). The wavefunctions defining the two ground KDs in disordered **1**_**Dy**_ are almost pure (98%) |*M*_*J*_| = 15/2 character; however, the first-excited KDs at 144/157 cm^–1^ feature significant mixing of wavefunctions and appreciable transverse components of the *g*-tensor. In addition, the easy axes of magnetization in the first-excited KDs are oriented at angles of 47.9 and 38.5° relative to the ground KD (Figure 5). The calculations therefore predict that the Orbach relaxation occurs via first the first KD with a barrier in the region of 144–157 cm^–1^ for **1**_**Dy**_, in reasonable agreement with the experimental barrier of 165(5) cm^–1^.

**Table 1:** Energies (cm^–1^) of the Eight KDs Corresponding to the Crystal-Field Split Ground ^6^H_15/2_ Multiplets of **1**_**Dy**_, **2**_**Dy**_, and **3**_**Dy**_

1_Dy_		2_Dy_	3_Dy_	
part A	part B		Dy1	Dy2
0	0	0	0	0
144	157	228	280	304
175	194	335	490	517
211	259	417	612	643
279	324	519	685	717
327	384	628	786	813
402	420	776	925	957
446	477	1060	1162	1205

The ground KD of **2**_**Dy**_ is characterized by *g*_*x*_ = 0.019, *g*_*y*_ = 0.035, and *g*_*z*_ = 19.59 with the wavefunction consisting of 95% |*M*_*J*_| = 15/2 character, which is highly axial and remarkably similar to disordered part B of **1**_**Dy**_. Although the first-excited KD features an easy axis of magnetization disposed at an angle of only 4.7° relative to the ground KD, appreciable mixing of the |*M*_*J*_| wavefunctions (Table S13) implies that **2**_**Dy**_ should display an energy barrier in the region of 228 cm^–1^. While the AC susceptibility of **2**_**Dy**_ is reminiscent of the related metallocenes [(Cp^ttt^)_2_DyCl] (for which a barrier could not be determined)^16^ and [(η^5^-C_5_^*i*^Pr_5_)Dy(η^5^-Cp*)(κ^2^-BH_4_)] (*U*_eff_ = 7 cm^–1^),^14^ the absence of a measurable energy barrier is clearly inconsistent with the *g*-tensor analysis. A possible reason for the inconsistency is that the hydride-donor atoms on the borohydride ligand are treated in optimized positions by the calculations. Since the crystal-field splitting is sensitive to small changes in molecular structure, it is possible that the axial character of **2**_**Dy**_ is overestimated by the calculations. The experimental data clearly indicate ground-state QTM, suggesting that intricate features of the spin-phonon coupling in this system are not accounted for by the calculations. Indeed, we have previously found similar discrepancies between measured and calculated barriers in metallocene-like dysprosium SMMs.^22^ Qualitative relaxation barriers for **1**_**Dy**_ and **2**_**Dy**_ are depicted in Figure 5, in which the possible pathways follow the states connected by non-negligible values of the transition magnetic moment matrix elements (Tables S16–S18). The barriers constructed for both disordered components of **1**_**Dy**_ illustrate that the dominant crossing transition occurs via the first-excited KD, whereas for **2**_**Dy**_, the most probable barrier-crossing transition is also calculated to occur via the first-excited KD, despite the dominant QTM observed experimentally.

**Figure 6:**
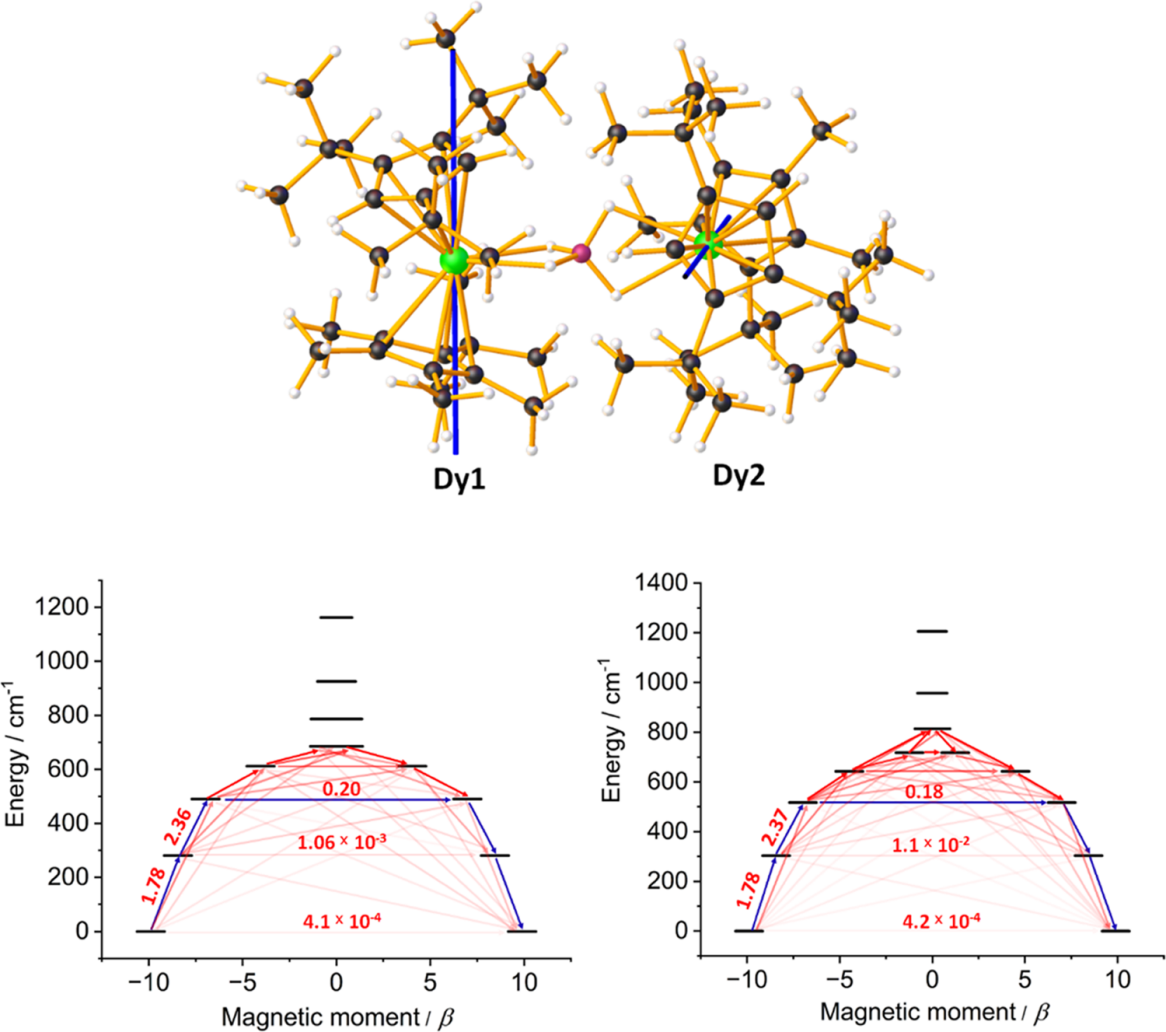
Principal magnetic axes (blue lines) of the ground KDs for **3**_**Dy**_, and relaxation barriers for Dy1 (left) and Dy2 (right). Arrows on the relaxation barriers represent possible relaxation processes, with associated numbers being the transition magnetic moments. Darker red shading represents transitions of higher probability, with blue arrows being the most probable pathway according to ab initio calculations. Green = Dy, pink = B, black = C, red = O, gray = H.

The changes in coordination geometry that accompany the formation of **3**_**Dy**_ are sufficient to induce stronger axiality and weaker equatorial components into the crystal fields experienced by the two inequivalent dysprosium centers. For Dy1, the *g*-tensors in the ground KD are *g*_*x*_ = 0.001, *g*_*y*_ = 0.001, *g*_*z*_ = 19.78, and *g*_*x*_ = 0.028, *g*_*y*_ = 0.035, *g*_*z*_ = 16.89 in the first-excited KD at 280 cm^–1^, with the associated principal magnetic axes being almost colinear (deviation of 2°, Tables 1, S14, and S15). The wavefunctions describing the two lowest-lying KDs for Dy1 are almost pure |*M*_*J*_| = 15/2 (98%) and |*M*_*J*_| = 13/2 (96%) character, respectively. The second-excited KD at 490 cm^–1^ also has strong axial character and is almost colinear with the ground KD (deviation of 4°); however, the transverse components are appreciable, i.e., *g*_*x*_ = 0.533, *g*_*y*_ = 0.717, *g*_*z*_ = 13.97, and significant mixing of the *M*_*J*_ states occurs. A very similar picture emerges for Dy2 in **3**_**Dy**_, for which the second-excited KD lies at 517 cm^–1^ (Table 1). The relaxation barrier and transition magnetic moments for **3_Dy_** therefore point toward dominant relaxation via the second-excited KD with a barrier in the region of 500 cm^–1^ (Figure 6 and Tables S19 and S20), in good agreement with the experimentally determined barrier of 534(19) cm^–1^.

Further understanding of the SMM properties of **1**_**Dy**_, **2**_**Dy**_, and **3**_**Dy**_ was obtained from a consideration of the ab initio calculated crystal-field parameters.^52^ Appreciable values of the axial parameter *B*_2_^0^ are found for all dysprosium centers, with gradual increases in axial character on moving through the series (Table S21). For the nonaxial parameters *B*_2_^*q*^ (*q* = ±2, ±1), smaller average contributions are found in **1**_**Dy**_ and **3**_**Dy**_, whereas for **2**_**Dy**_, a relatively large value of *B*_2_^2^ occurs along with a larger average nonaxial contribution originating from the terminally bound, equatorial κ^2^-borohydride ligand. The relatively large nonaxial contribution in **2**_**Dy**_ should result in fast QTM within the ground state, broadly consistent with experimental observations. In contrast, the molecular structure of **1**_**Dy**_ shows that the κ^3^-borohydride ligands are tilted away from equatorial towards axial positions. While the μ:κ^2^:κ^2^-borohydride ligand in **3**_**Dy**_ adopts a similar equatorial position to its counterpart in **2**_**Dy**_, the Dy–H and Dy···B distances are substantially longer in the bimetallic complex, thereby significantly weakening the equatorial component of the crystal field.

Finally, to analyze the exchange interactions in **3**_**Dy**_, we simulated the experimental magnetic susceptibility and magnetization data using the Lines model and an Ising-type Hamiltonian represented as eq 1, implemented using the POLY_ANISO module in ORCA.
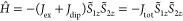
1In eq 1, *S̃* denotes the pseudospin-1/2 operator for the ground state of each Dy^3+^ ion, and *J*_ex_ and *J*_dip_ are the exchange and dipolar coupling constants, respectively. Good fits of the experimental χ_M_*T*(*T*) and *M*(*H*) data were obtained with a very small intermolecular term of *zJ* = −0.003 cm^–1^ (Figure S85 and Table S22), yielding *J*_ex_ = −0.7 cm^–1^ and *J*_dip_ = +0.04 cm^–1^ and, hence, *J*_tot_ = −0.66 cm^–1^. Furthermore, the calculated tunnel splitting between the ground states in **3**_**Dy**_ is small (on the order of 10^–7^ cm^–1^) (Table S23), suggesting the suppression of ground-state QTM in this complex. This theoretical observation agrees well with the fitting of the AC susceptibility of **3**_**Dy**_ using only Orbach and Raman processes, compared to the analogous fitting for **Dy@3**_**Y**_, which required a QTM term. The observation is also consistent with the wider hysteresis loops for **3**_**Dy**_ at zero field compared to **Dy@3**_**Y**_ at 2 K (Figure 4). An additional noteworthy feature in **3**_**Dy**_ was observed in the lowest-lying exchange states, where the *g*_*zz*_-tensor approaches an intermediate value of 25.01 rather than zero, which would occur for more strongly antiferromagnetically coupled Dy^3+^ centers. This is likely to be a consequence of the relative alignment of the principal magnetic axes of the ground KDs in Dy1 and Dy2, which are disposed at an angle of 78.8°, comparable to the dihedral angle of 68.70(10)° formed by the two Cp_cent_^ttt^–Dy–Cp_cent_^Me4t^ planes. Overall, the absence of QTM in **3**_**Dy**_ is linked to the borohydride ligand in terms of its bonding to dysprosium and the structure-directing influence of the tetrahedral μ:κ^2^:κ^2^-coordination mode, which results in canting of the metallocene units in a way that does not occur in multimetallic SMMs where the Dy^3+^ ions are either strictly or almost symmetry-related.

## Conclusions

In conclusion, the borohydride-bridged bimetallic metallocenium cations **3**_**M**_ were synthesized from the metallocene precursors **2**_**M**_, which, in turn, were obtained from the half-sandwich complexes **1**_**M**_. The ligand environment in **1**_**M**_ is pseudo-axially symmetrical, with the Cp^ttt^ centroid defining the axis and with the *H*-donor atoms of the borohydride ligands occupying a mixture of axial and equatorial sites. In **2**_**M**_, the κ^2^-borohydride ligand occupies a well-defined equatorial position relative to the two axial cyclopentadienyl ligands. SMM behavior with an effective energy barrier of 165(5) cm^–1^ and relaxation via the first-excited KD were determined for **1**_**Dy**_, whereas **2**_**Dy**_ is not an SMM. In **3**_**Dy**_, a key role was identified for the μ:κ^2^:κ^2^-borohydride and the cyclopentadienyl ligands in deriving a magnetostructural correlation to describe the SMM properties. The structure of **3**_**Dy**_ supports SMM behavior consisting of Orbach relaxation via the second-excited KD at higher temperatures, with a large barrier of 534(19) cm^–1^, and Raman relaxation but, notably, not QTM at low temperatures. Unexpectedly, a study of the magnetically dilute material **Dy@3**_**Y**_ led to the onset of QTM as the dominant relaxation mechanism at low temperatures, as reflected in closed magnetic hysteresis loops at 2 K, whereas the loops for **3**_**Dy**_ are open at zero field. An ab initio computational study of all three dysprosium species provided a theoretical basis for the magnetization dynamics consistent with the experimental observations. The calculated *g*-tensors and crystal field parameters revealed strong axial and weak equatorial character for the dysprosium centers in **1**_**Dy**_ and **3**_**Dy**_, and non-negligible equatorial components in **2**_**Dy**_. Further studies on exchange-coupled multimetallic lanthanide borohydride compounds are underway in our laboratory.

## Supporting Information Available

Synthesis, spectroscopic characterization, crystallography details, magnetic measurements, and computational details (PDF)

## References

[ref1] Day B. M.; Guo F.-S.; Layfield R. A. Cyclopentadienyl Ligands in Lanthanide Single-Molecule Magnets: One Ring To Rule Them All?. Acc. Chem. Res. 2018, 51, 1880–1889. 10.1021/acs.accounts.8b00270. 30091896

[ref2] Harriman K. L. M.; Murugesu M. An Organolanthanide Building Block Approach to Single-Molecule Magnets. Acc. Chem. Res. 2016, 49, 1158–1167. 10.1021/acs.accounts.6b00100. 27195740

[ref3] Meng Y.-S.; Jiang S.-D.; Wang B.-W.; Gao S. Understanding the Magnetic Anisotropy toward Single-Ion Magnets. Acc. Chem. Res. 2016, 49, 2381–2389. 10.1021/acs.accounts.6b00222. 27768294

[ref4] Zhang P.; Benner F.; Chilton N. F.; Demir S. Organometallic Lanthanide Bismuth Cluster Single-Molecule Magnets. Chem 2022, 8, 717–730. 10.1016/j.chempr.2021.11.007.

[ref5] Pugh T.; Chilton N. F.; Layfield R. A. Antimony-Ligated Dysprosium Single-Molecule Magnets as Catalysts for Stibine Dehydrocoupling. Chem. Sci. 2017, 8, 2073–2080. 10.1039/C6SC04465D. 28451326 PMC5399632

[ref6] Pugh T.; Vieru V.; Chibotaru L. F.; Layfield R. A. Magneto-Structural Correlations in Arsenic- and Selenium-Ligated Dysprosium Single-Molecule Magnets. Chem. Sci. 2016, 7, 2128–2137. 10.1039/C5SC03755G. 29899940 PMC5968533

[ref7] Pugh T.; Tuna F.; Ungur L.; Collison D.; McInnes E. J. L.; Chibotaru L. F.; Layfield R. A. Influencing the Properties of Dysprosium Single-Molecule Magnets with Phosphorus Donor Ligands. Nat. Commun. 2015, 6, 7492 10.1038/ncomms8492. 26130418 PMC4507012

[ref8] Tuna F.; Smith C. A.; Bodensteiner M.; Ungur L.; Chibotaru L. F.; McInnes E. J. L.; Winpenny R. E. P.; Collison D.; Layfield R. A. A High Anisotropy Barrier in a Sulfur-Bridged Organodysprosium Single-Molecule Magnet. Angew. Chem., Int. Ed. 2012, 51, 6976–6980. 10.1002/anie.201202497. 22684888

[ref9] Errulat D.; Gabidullin B.; Mansikkamäki A.; Murugesu M. Two Heads Are Better than One: Improving Magnetic Relaxation in the Dysprosium Metallocene [Dy_2_Cp*_4_(μ-BPh_4_)][Al(OC(CF_3_)_3_)_4_] upon Dimerization by Use of an Exceptionally Weakly-Coordinating Anion. Chem. Commun. 2020, 56, 5937–5940. 10.1039/D0CC01980A. 32347247

[ref10] Demir S.; Gonzalez M. I.; Darago L. E.; Evans W. J.; Long J. R. Giant Coercivity and High Magnetic Blocking Temperatures for N_2_^3–^ Radical-Bridged Dilanthanide Complexes upon Ligand Dissociation. Nat. Commun. 2017, 8, 2144 10.1038/s41467-017-01553-w. 29247236 PMC5732206

[ref11] Gould C. A.; Darago L. E.; Gonzalez M. I.; Demir S.; Long J. R. A Trinuclear Radical-Bridged Lanthanide Single-Molecule Magnet. Angew. Chem., Int. Ed. 2017, 56, 10103–10107. 10.1002/anie.201612271. 28157259

[ref12] Gould C. A.; Mu E.; Vieru V.; Darago L. E.; Chakarawet K.; Gonzalez M. I.; Demir S.; Long J. R. Substituent Effects on Exchange Coupling and Magnetic Relaxation in 2,2′-Bipyrimidine Radical-Bridged Dilanthanide Complexes. J. Am. Chem. Soc. 2020, 142, 21197–21209. 10.1021/jacs.0c10612. 33322909

[ref13] Mavragani N.; Errulat D.; Gμlico D. A.; Kitos A. A.; Mansikkamäki A.; Murugesu M. Radical-Bridged Ln_4_ Metallocene Complexes with Strong Magnetic Coupling and a Large Coercive Field. Angew. Chem., Int. Ed. 2021, 60, 24206–24213. 10.1002/anie.202110813. 34427984

[ref14] Guo F.-S.; Day B. M.; Chen Y.-C.; Tong M.-L.; Mansikkamäki A.; Layfield R. A. Magnetic Hysteresis up to 80 Kelvin in a Dysprosium Metallocene Single-Molecule Magnet. Science 2018, 362, 1400–1403. 10.1126/science.aav0652. 30337456

[ref15] Goodwin C. A. P.; Ortu F.; Reta D.; Chilton N. F.; Mills D. P. Molecular Magnetic Hysteresis at 60 Kelvin in Dysprosocenium. Nature 2017, 548, 439 10.1038/nature23447. 28836589

[ref16] Guo F.-S.; Day B. M.; Chen Y.-C.; Tong M.-L.; Mansikkamäki A.; Layfield R. A. A Dysprosium Metallocene Single-Molecule Magnet Functioning at the Axial Limit. Angew. Chem., Int. Ed. 2017, 56, 11445–11449. 10.1002/anie.201705426. 28586163

[ref17] Randall McClain K.; Gould C. A.; Chakarawet K.; Teat S. J.; Groshens T. J.; Long J. R.; Harvey B. G. High-Temperature Magnetic Blocking and Magneto-Structural Correlations in a Series of Dysprosium(III) Metallocenium Single-Molecule Magnets. Chem. Sci. 2018, 9, 8492–8503. 10.1039/C8SC03907K. 30568773 PMC6256727

[ref18] Vanjak J. C.; Wilkins B. O.; Vieru V.; Bhuvanesh N. S.; Reibenspies J. H.; Martin C. D.; Chibotaru L. F.; Nippe M. A High-Performance Single-Molecule Magnet Utilizing Dianionic Aminoborolide Ligands. J. Am. Chem. Soc. 2022, 144, 17743–17747. 10.1021/jacs.2c06698. 36162057

[ref19] Guo F.-S.; He M.; Huang G.-Z.; R Giblin S.; Billington D.; W Heinemann F.; Tong M.-L.; Mansikkamäki A.; A Layfield R. Discovery of a Dysprosium Metallocene Single-Molecule Magnet with Two High-Temperature Orbach Processes. Inorg. Chem. 2022, 61, 6017–6025. 10.1021/acs.inorgchem.1c03980. 35420419 PMC9044448

[ref20] Evans P.; Reta D.; F S Whitehead G.; F Chilton N.; P Mills D. Bis-Monophospholyl Dysprosium Cation Showing Magnetic Hysteresis at 48 K. J. Am. Chem. Soc. 2019, 141, 19935–19940. 10.1021/jacs.9b11515. 31751131 PMC7007226

[ref21] Durrant J. P.; Tang J.; Mansikkamäki A.; Layfield R. A. Enhanced Single-Molecule Magnetism in Dysprosium Complexes of a Pristine Cyclobutadienyl Ligand. Chem. Commun. 2020, 56, 4708–4711. 10.1039/D0CC01722A. 32215423

[ref22] Durrant J. P.; Day B. M.; Tang J.; Mansikkamäki A.; Layfield R. A. Dominance of Cyclobutadienyl Over Cyclopentadienyl in the Crystal Field Splitting in Dysprosium Single-Molecule Magnets. Angew. Chem., Int. Ed. 2022, 61, e202200525 10.1002/anie.202200525. PMC9302998 35108431

[ref23] Gould C. A.; McClain K. R.; Yu J. M.; Groshens T. J.; Furche F.; Harvey B. G.; Long J. R. Synthesis and Magnetism of Neutral, Linear Metallocene Complexes of Terbium(II) and Dysprosium(II). J. Am. Chem. Soc. 2019, 141, 12967–12973. 10.1021/jacs.9b05816. 31375028

[ref24] Gould C. A.; McClain K. R.; Reta D.; Kragskow J. G.; Marchiori D. A.; Lachman E.; Choi E. S.; Analytis J. G.; Britt R. D.; Chilton N. F.; Harvey B. G. Ultrahard Magnetism from Mixed-Valence Dilanthanide Complexes with Metal-Metal Bonding. Science 2022, 375, 198–202. 10.1126/science.abl5470. 35025637

[ref25] Bernbeck M. G.; Hilgar J. D.; Rinehart J. D. Probing Axial Anisotropy in Dinuclear Alkoxide-Bridged Er–COT Single-Molecule Magnets. Polyhedron 2020, 175, 114206 10.1016/j.poly.2019.114206.

[ref26] Hilgar J. D.; Flores B. S.; Rinehart J. D. Ferromagnetic Coupling in a Chloride-Bridged Erbium Single-Molecule Magnet. Chem. Commun. 2017, 53, 7322–7324. 10.1039/C7CC02356A. 28487927

[ref27] Hilgar J. D.; Bernbeck M. G.; Rinehart J. D. Million-Fold Relaxation Time Enhancement across a Series of Phosphino-Supported Erbium Single-Molecule Magnets. J. Am. Chem. Soc. 2019, 141, 1913–1917. 10.1021/jacs.8b13514. 30672697

[ref28] Hilgar J. D.; Bernbeck M. G.; Flores B. S.; Rinehart J. D. Metal–Ligand Pair Anisotropy in a Series of Mononuclear Er–COT Complexes. Chem. Sci. 2018, 9, 7204–7209. 10.1039/C8SC01361F. 30746111 PMC6335627

[ref29] Meihaus K. R.; Long J. R. Magnetic Blocking at 10 K and a Dipolar-Mediated Avalanche in Salts of the Bis(η^8^-Cyclooctatetraenide) Complex [Er(COT)_2_]–. J. Am. Chem. Soc. 2013, 135, 17952–17957. 10.1021/ja4094814. 24188004

[ref30] Le Roy J. J.; Ungur L.; Korobkov I.; Chibotaru L. F.; Murugesu M. Coupling Strategies to Enhance Single-Molecule Magnet Properties of Erbium–Cyclooctatetraenyl Complexes. J. Am. Chem. Soc. 2014, 136, 8003–8010. 10.1021/ja5022552. 24805804

[ref31] Ungur L.; Le Roy J. J.; Korobkov I.; Murugesu M.; Chibotaru L. F. Fine-Tuning the Local Symmetry to Attain Record Blocking Temperature and Magnetic Remanence in a Single-Ion Magnet. Angew. Chem., Int. Ed. 2014, 53, 4413–4417. 10.1002/anie.201310451. 24652777

[ref32] aJiang S. Da.; Wang B. W.; Sun H. L.; Wang Z. M.; Gao S. An Organometallic Single-Ion Magnet. J. Am. Chem. Soc. 2011, 133, 4730–4733. 10.1021/ja200198v. 21401130

[ref33] Münzfeld L.; Schoo C.; Bestgen S.; Moreno-Pineda E.; Köppe R.; Ruben M.; Roesky P. W. Synthesis, Structures and Magnetic Properties of [(η^9^-C_9_H_9_)Ln(η^8^-C_8_H_8_)] Super Sandwich Complexes. Nat. Commun. 2019, 10, 3135 10.1038/s41467-019-10976-6. 31316061 PMC6637141

[ref34] Moutet J.; Schleinitz J.; La Droitte L.; Tricoire M.; Pointillart F.; Gendron F.; Simler T.; Clavaguéra C.; Le Guennic B.; Cador O.; Nocton G. Bis-Cyclooctatetraenyl Thulium(II): Highly Reducing Lanthanide Sandwich Single-Molecule Magnets. Angew. Chem., Int. Ed. 2021, 60, 6042–6046. 10.1002/anie.202015428. PMC7613950 36530221

[ref35] Moreno-Pineda E.; Wernsdorfer W. Measuring Molecular Magnets for Quantum Technologies. Nat. Rev. Phys. 2021, 3, 645–659. 10.1038/s42254-021-00340-3.

[ref36] Coronado E. Molecular Magnetism: From Chemical Design to Spin Control in Molecules, Materials and Devices. Nat. Rev. Mater. 2020, 5, 87–104. 10.1038/s41578-019-0146-8.

[ref37] Atzori M.; Sessoli R. The Second Quantum Revolution: Role and Challenges of Molecular Chemistry. J. Am. Chem. Soc. 2019, 141, 11339–11352. 10.1021/jacs.9b00984. 31287678

[ref38] Feng M.; Tong M.-L. Single Ion Magnets from 3d to 5f: Developments and Strategies. Chem. - Eur. J. 2018, 24, 7574–7594. 10.1002/chem.201705761. 29385282

[ref39] Liu J.-L.; Chen Y.-C.; Tong M.-L. Symmetry Strategies for High Performance Lanthanide-Based Single-Molecule Magnets. Chem. Soc. Rev. 2018, 47, 2431–2453. 10.1039/C7CS00266A. 29492482

[ref40] Zhu Z.; Guo M.; Li X.-L.; Tang J. Molecular Magnetism of Lanthanide: Advances and Perspectives. Coord. Chem. Rev. 2019, 378, 350–364. 10.1016/j.ccr.2017.10.030.

[ref41] Zabala-Lekuona A.; Seco J. M.; Colacio E. Single-Molecule Magnets: From Mn12-Ac to Dysprosium Metallocenes, a Travel in Time. Coord. Chem. Rev. 2021, 441, 213984 10.1016/j.ccr.2021.213984.

[ref42] Briganti M.; Lucaccini E.; Chelazzi L.; Ciattini S.; Sorace L.; Sessoli R.; Totti F.; Perfetti M. Magnetic Anisotropy Trends along a Full 4f-Series: The f^n+7^ Effect. J. Am. Chem. Soc. 2021, 143, 8108–8115. 10.1021/jacs.1c02502. 34024105 PMC8297734

[ref43] He M.; Guo F.-S.; Tang J.; Mansikkamäki A.; Layfield R. A. Fulvalene as a Platform for the Synthesis of a Dimetallic Dysprosocenium Single-Molecule Magnet. Chem. Sci. 2020, 11, 5745–5752. 10.1039/D0SC02033H. 32832050 PMC7422961

[ref44] He M.; Guo F.-S.; Tang J.; Mansikkamä A.; Layfield R. A. Synthesis and Single-Molecule Magnet Properties of a Trimetallic Dysprosium Metallocene Cation. Chem. Commun. 2021, 57, 6396–6399. 10.1039/D1CC02139G. PMC8240697 34085074

[ref45] Wegner W.; van Leusen J.; Majewski J.; Grochala W.; Kögerler P. Borohydride as Magnetic Superexchange Pathway in Late Lanthanide Borohydrides. Eur. J. Inorg. Chem. 2019, 2019, 1776–1783. 10.1002/ejic.201801488.

[ref46] Nocton G.; Ricard L. N-Aromatic Heterocycle Adducts of Bulky [1,2,4-(Me_3_C)_3_C_5_H_2_]_2_Sm: Synthesis, Structure and Solution Analysis. Dalton Trans. 2014, 43, 4380–4387. 10.1039/C3DT52641K. 24231794

[ref47] Benelli C.; Gatteschi D. Introduction to Molecular Magnetism; John Wiley & Sons, Ltd., 2015; pp 1–23.

[ref48] Burns C. P.; Wilkins B. O.; Dickie C. M.; Latendresse T. P.; Vernier L.; Vignesh K. R.; Bhuvanesh N. S.; Nippe M. A Comparative Study of Magnetization Dynamics in Dinuclear Dysprosium Complexes Featuring Bridging Chloride or Trifluoromethanesulfonate Ligands. Chem. Commun. 2017, 53, 8419–8422. 10.1039/C7CC02457F. 28702526

[ref49] Gysler M.; El Hallak F.; Ungur L.; Marx R.; Hakl M.; Neugebauer P.; Rechkemmer Y.; Lan Y.; Sheikin I.; Orlita M.; Anson C. E.; Powell A. K.; Sessoli R.; Chibotaru L. F.; van Slageren J. Multitechnique Investigation of Dy_3_ – Implications for Coupled Lanthanide Clusters. Chem. Sci. 2016, 7, 4347–4354. 10.1039/C6SC00318D. 30155081 PMC6013819

[ref50] Pugh T.; Chilton N. F.; Layfield R. A. A Low-Symmetry Dysprosium Metallocene Single-Molecule Magnet with a High Anisotropy Barrier. Angew. Chem., Int. Ed. 2016, 55, 11082–11085. 10.1002/anie.201604346. 27460170

[ref51] Neese F.; Wennmohs F.; Becker U.; Riplinger C. The ORCA Quantum Chemistry Program Package. J. Chem. Phys. 2020, 152, 224108 10.1063/5.0004608. 32534543

[ref52] Ungur L.; Chibotaru L. F. Ab Initio Crystal Field for Lanthanides. Chem. - Eur. J. 2017, 23, 3708–3718. 10.1002/chem.201605102. 27983776

