# Supplementary material for: Structural and Magnetization Dynamics of Borohydride-Bridged Rare-Earth Metallocenium Cations
Source: Inorg Chem. 2023 Jun 14;62(25):9924–33. doi: 10.1021/acs.inorgchem.3c01038 (PMC10302870; doi:10.1021/acs.inorgchem.3c01038)
Supplement: Supplementary file 1 — ic3c01038_si_001.pdf [file ic3c01038_si_001.pdf]

Supporting Information

Structural and Magnetization Dynamics of Borohydride-Bridged Rare-Earth Metallocenium Cations

Christopher G. T. Price,[a] Arpan Mondal,[a] James P. Durrant,[a] Jinkui Tang,[b] and Richard A. Layfield*[a]

[a] Department of Chemistry, School of Life Sciences, University of Sussex, Brighton, BN1 9QR, U.K. E-mail: r.layfield@sussex.ac.uk

[b] Changchun Institute of Applied Chemistry, Chinese Academy of Sciences, Renmin Street 5626, 130022 Changchun, China

Contents

Synthesis details	S1-S4
X-ray crystallography	S5-S11
IR spectra and NMR spectra	S12-S80
Magnetic property measurements	S81-S89
Computational details	S89-S102
References	S103

SYNTHESIS DETAILS

General considerations

All reactions were carried out under rigorous anaerobic, anhydrous conditions using argon or nitrogen atmospheres and standard Schlenk and glove-box techniques. All solvents were refluxed over an appropriate drying agent for a minimum of three days (molten potassium for benzene-D_6, toluene, toluene-D_8, THF, Na/K alloy for hexane, and CaH_2 for chlorobenzene-D_5), and then distilled and degassed via a minimum of three freeze-pump-thaw cycles. Solvents were then stored in ampoules over potassium mirrors (hexane and toluene) or activated 4 Å molecular sieves (benzene-D_6, toluene-D_8, chlorobenzene-D_5). Elemental analyses were carried out at MEDAC Ltd. NMR spectra were recorded on a Varian VNMR S400 spectrometer operating at 30 °C, unless otherwise stated, at frequencies of 400 MHz (^{1}H), 128 MHz (^{11}B), 100 MHz (^{13}C), and 376 MHz (^{19}F). Literature procedures were used to synthesise $[Ln(BH_4)_3(THF)_3]$ (Ln = Y, Dy, Lu),[1] $Cp^{ttt}H$,[2] and $Cp^{Me4t}H$.[3] Both KCp^{ttt} and $NaCp^{Me4t}$ were synthesised by reacting $Cp^{ttt}H$ and $Cp^{Me4t}H$ with $KN(SiMe_3)_2$ or $NaNH_2$, respectively, in toluene overnight.

- **Synthesis of $[Y(Cp^{ttt})(\kappa^3\text{-}BH_4)_2(THF)]$ (1_Y)**

THF (20 mL) was added to a mixture of solid $[Y(BH_4)_3(THF)_3]$ (321 mg, 0.92 mmol) and KCp^{ttt} (250 mg, 0.92 mmol) and the resulting colourless suspension was stirred at 60 °C for 24 hours. The solvent was removed under vacuum and the residual solids extracted with hot hexane (3 × 10 mL) and filtered. The filtrate volume was reduced under vacuum until crystallisation began to occur, then the crystals were re-dissolved with gentle heating and the resulting solution stored at –35 °C overnight. Colourless crystals of $\mathbf{1_Y}$ suitable for X-ray crystallography were isolated (299 mg, 77%).

Elemental analysis (%), found (calculated) for $C_{21}H_{45}OB_2Y$: C 61.19 (61.03); H 10.88 (10.97).

FTIR ($\tilde{\upsilon}$/cm^{-1}): 3000-2800 (m, br, C-H), 2470 (s, B-H$_t$), 2400-2050 (m, br, B-H$_b$).

$^{1}H\{^{11}B\}$ NMR (δ/ppm): 1.00 (4H, m, CH_2), 1.06 (8H, s, BH_4), 1.26 (9H, s, ^{t}Bu), 1.55 (18H, s, ^{t}Bu), 3.53 (4H, m, CH_2O), 6.40 (2H, s, $C_5\underline{H_2}{}^{t}Bu_3$). **$^{13}C\{^{1}H\}$ NMR** (δ/ppm): 24.83 (CH_2), 31.94 (4-CH_3), 32.77 (4-$\underline{C}Me_3$), 34.09 (1,2-CH_3), 34.33 (1,2-$\underline{C}Me_3$), 73.70 (CH_2O), 111.37 (1,2-C_5 ring), 137.81 (4-C_5 ring), 137.84 (3,5-C_5 ring). **^{11}B NMR** (δ/ppm): –22.69 (quintet, BH_4, $^{1}J_{BH}$ = 84 Hz). **$^{11}B\{^{1}H\}$ NMR** (δ/ppm): –22.69 (s, BH_4).

- **Synthesis of [Dy(Cp^{ttt})(κ^3-BH_4)$_2$(THF)] (1_{Dy})**

Compound **1_{Dy}** was synthesised via the same procedure as for **1_Y**, using [Dy(BH_4)$_3$(THF)$_3$] (445 mg, 1.05 mmol) and KCp^{ttt} (286 mg, 1.05 mmol), and isolated as pale yellow crystals (370 mg, 71%).

Elemental analysis (%), found (calculated) for $C_{21}H_{45}OB_2Dy$: C 51.68 (51.80); H 9.19 (9.32).

FTIR ($\tilde{\nu}$/cm^{-1}): 3000-2800 (m, br, C-H), 2470 (s, B-H_t), 2400-2050 (m, br, B-H_b).

- **Synthesis of [Lu(Cp^{ttt})(κ^3-BH_4)$_2$(THF)] (1_{Lu})**

Compound **1_{Lu}** was synthesised via the same procedure as for **1_Y**, using [Lu(BH_4)$_3$(THF)$_3$] (400 mg, 0.92 mmol) and KCp^{ttt} (250 mg, 0.92 mmol), and isolated as colourless crystals (372 mg, 80%).

Elemental analysis (%), found (calculated) for $C_{21}H_{45}OB_2Lu$: C 50.43 (50.51); H 8.99 (9.08). FTIR ($\tilde{\nu}$/cm^{-1}): 3000-2800 (m, br, C-H), 2470 (s, B-H_t), 2400-2050 (m, br, B-H_b).

^{1}H NMR (δ/ppm): 1.02 (4H, m, CH_2), 1.26 (9H, s, ^{t}Bu), 1.56 (18H, s, ^{t}Bu), 1.83 (8H, quartet, BH_4, $^{1}J_{HB}$ = 96 Hz), 3.57 (4H, m, CH_2O), 6.30 (2H, s, $C_5\underline{H_2}{}^{t}Bu_3$). **$^{13}C\{^{1}H\}$ NMR** (δ/ppm): 24.92 (CH_2), 31.94 (4-CH_3), 32.80 (4-$\underline{C}Me_3$), 34.16 (1,2-CH_3), 34.31 (1,2-$\underline{C}Me_3$), 74.52 (CH_2O), 110.25 (1,2-C_5 ring), 136.50 (4-C_5 ring), 136.66 (3,5-C_5 ring). **^{11}B NMR** (δ/ppm): –22.62 (quintet, BH_4, $^{1}J_{BH}$ = 85 Hz). **$^{11}B\{^{1}H\}$ NMR** (δ/ppm): –22.62 (s, BH_4).

- **Synthesis of [Y(Cp^{ttt})(Cp^{Me4t})(κ^2-BH_4)] (2_Y)**

Toluene (20 mL) was added to a mixture of solid **1_Y** (421 mg, 0.99 mmol) and NaCp^{Me4t} (199 mg, 0.99 mmol) and the resulting colourless suspension was stirred at 110 °C for 72 hours. The solvent was removed under vacuum and the residual solids extracted with hexane (2 × 10 mL) and filtered. The filtrate volume was reduced under vacuum until crystals began to form, then the crystals were re-dissolved with gentle heating and the resulting solution stored at –20 °C for 24 hours. Colourless crystals of **2_Y** suitable for X-ray crystallography were isolated (301 mg, 59%).

Elemental analysis (%), found (calculated) for $C_{30}H_{54}BY$: C 70.15 (70.04); H 10.44 (10.58).

FTIR ($\tilde{\nu}$/cm^{-1}): 3050-2700 (m, br, C-H), 2450-2250 (m, s, B-H_t), 2250-1900 (m, br, B-H_b).

$^{1}H\{^{11}B\}$ NMR (δ/ppm): 1.17 (9H, s, Cp^{ttt} ^{t}Bu), 1.26 (9H, s, Cp^{Me4t} ^{t}Bu), 1.33 (4H, d, BH_4, $^{1}J_{HY}$ = 12 Hz), 1.44 (18H, s, Cp^{ttt} ^{t}Bu), 1.96 (6H, s, Cp^{Me4t} 1,4-Me), 2.44 (6H, s, Cp^{Me4t} 2,3-Me), 6.58 (2H, s, $C_5\underline{H_2}{}^{t}Bu_3$). **$^{13}C\{^{1}H\}$ NMR** (δ/ppm): 12.78 (Cp^{Me4t} 2,3-Me), 16.90 (Cp^{Me4t} 1,4-Me), 31.91 (Cp^{ttt} 4-C($\underline{C}H_3$)$_3$), 32.18 (Cp^{ttt} 1,2-C($\underline{C}H_3$)$_3$), 32.70 (Cp^{ttt} 4-$\underline{C}Me_3$), 33.40 (Cp^{Me4t} C($\underline{C}H_3$)$_3$), 34.40 (Cp^{ttt} 1,2-$\underline{C}$(CH_3)$_3$), 35.18 (Cp^{Me4t} $\underline{C}$(CH_3)$_3$), 115.19 (Cp^{Me4t} 1,4-C_5 ring), 122.63 (d, Cp^{Me4t} 2,3-C_5 ring $^{1}J_{CY}$ = 2 Hz), 134.95 (d, Cp^{Me4t} 5-C_5 ring, $^{1}J_{CY}$ = 1 Hz), 135.89 (d, Cp^{ttt} 1,2-C_5 ring, $^{1}J_{CY}$ = 2 Hz), 136.32 (d, Cp^{ttt} 4-C_5 ring, $^{1}J_{CY}$ = 2 Hz). **^{11}B NMR** (δ/ppm): –16.16 (quintet, BH_4, $^{1}J_{BH}$ = 78 Hz). **$^{11}B\{^{1}H\}$ NMR** (δ/ppm): –16.22 (s, BH_4).

- **Synthesis of [Dy(Cp^{ttt})(Cp^{Me4t})(κ^2-BH$_4$)] (2$_{Dy}$)**

Compound **2$_{Dy}$** was synthesised via the same procedure as for **2$_Y$**, using **1$_{Dy}$** (377 mg, 0.76 mmol) and NaCp^{Me4t} (152 mg, 0.76 mmol), and isolated as pale-yellow crystals (332 mg, 75%).

Elemental analysis (%), found (calculated) for $C_{30}H_{54}BDy$: C 61.36 (61.27); H 9.27 (9.26).

FTIR ($\tilde{\nu}$/cm^{-1}): 3050-2700 (m, br, C-H), 2450-2250 (m, s, B-H$_T$), 2250-1900 (m, br, B-H$_B$).

- **Synthesis of [Lu(Cp^{ttt})(Cp^{Me4t})(κ^2-BH$_4$)] (2$_{Lu}$)**

Compound **2$_{Lu}$** was synthesised via the same procedure as for **2$_Y$**, using **1$_{Lu}$** (565 mg, 1.11 mmol) and NaCp^{Me4t} (222 mg, 1.11 mmol), and isolated as colourless crystals (198 mg, 30%).

Elemental analysis (%), found (calculated) for $C_{30}H_{54}BLu$: C 60.09 (60.00); H 9.12 (9.06).

FTIR ($\tilde{\nu}$/cm^{-1}): 3050-2700 (m, br, C-H), 2450-2250 (m, s, B-H$_T$), 2250-1900 (m, br, B-H$_B$).

^{1}H{^{11}B} NMR (δ/ppm): 1.15 (9H, s, Cp^{ttt} ^{t}Bu), 1.26 (9H, s, Cp^{Me4t} ^{t}Bu), 1.45 (18H, s, Cp^{ttt} ^{t}Bu), 1.96 (4H, s, BH$_4$), 1.97 (6H, s, Cp^{Me4t} 1,4-Me), 2.49 (6H, s, Cp^{Me4t} 2,3-Me), 6.63 (2H, s, C$_5$$\underline{H_2}^{t}Bu_3$). **^{13}C{^{1}H} NMR** (δ/ppm): 13.17 (Cp^{Me4t} 2,3-Me), 17.41 (Cp^{Me4t} 1,4-Me), 32.29 (Cp^{ttt} 4-C($\underline{C}$H$_3$)$_3$), 32.54 (Cp^{Me4t} C($\underline{C}$H$_3$)$_3$), 33.19 (Cp^{ttt} 4-$\underline{C}$Me$_3$), 33.89 (Cp^{ttt} 1,2-C($\underline{C}$H$_3$)$_3$), 34.82 (Cp^{ttt} 1,2-$\underline{C}$(CH$_3$)$_3$), 35.49 (Cp^{Me4t} $\underline{C}$Me)$_3$), 115.73 (Cp^{Me4t} 1,4-ring), 122.15 (Cp^{Me4t} 2,3-ring), 135.67 (Cp^{Me4t} 5-ring), 151.59 (Cp^{ttt} 3,5-ring); the remaining ring carbons are obscured by the D$_8$-toluene signals. **^{11}B NMR** (δ/ppm): –16.56 (quintent, BH$_4$, $^1J_{BH}$ = 90 Hz). **^{11}B{^{1}H}** NMR (δ/ppm): –16.51 (s, BH$_4$).

- **Synthesis of [{Y(Cp^{ttt})(Cp^{Me4t})}$_2$(μ-κ^2:κ^2-BH$_4$)][B(C$_6$F$_5$)$_4$] (3$_Y$)**

A solution of **2$_Y$** (211 mg, 0.41 mmol) in hexane (8 mL) was added dropwise to a stirred suspension of [CPh$_3$][B(C$_6$F$_5$)$_4$] (189 mg, 0.20 mmol) in hexane (1 mL). The solids quickly became sticky, and the mixture was stirred for 72 hours at room temperature, after which a white suspension had formed alongside residual sticky solids. A few drops of diethyl ether were added, and the solids were agitated with a spatula, and this process repeated with additional portions of diethyl ether until all sticky solids had become white powder. The solvent was removed by filtration and the off-white solids washed with hexane (4 × 5 mL) and then cold (–35 °C) diethyl ether (2 mL). Drying the solid under vacuum yielded **3$_Y$** as a white powder (208 mg, 81%). Single crystals suitable for X-ray crystallography were grown via slow diffusion of heptane into a toluene solution of **3$_Y$** at room temperature.

Elemental analysis (%), found (calculated) for $C_{84}H_{104}B_2F_{20}Y_2$: C 59.71 (59.59); H 6.23 (6.19).

FTIR ($\tilde{\nu}$/cm^{-1}): 3050-2700 (m, br, C-H), 2400-2150 (s, B-H$_B$).

^{1}H{^{11}B} NMR (δ/ppm): 0.65 (4H, br s, BH$_4$), 1.15; 1.20 (36 H, 2 s, Cp^{ttt} ^{t}Bu), 1.29 (9H, s, ^{t}Bu), 1.49 (9H, s, ^{t}Bu), 1.74 (6H, s, Me), 2.23; 2.28; 2.32; 2.36 (18H, 4 s, Me), 6.42; 6.53; 6.61 (4H, 3 s, C$_5$$\underline{H_2}^{t}Bu_3$). **^{13}C{^{1}H} NMR** (δ/ppm): 12.43 (Me), 13.29 (Me), 15.90 (Me), 17.49 (Me), 31.60; 31.94; 32.41; 33.10; 33.66; 33.88; 35.08; 35.25 ($\underline{C}$Me$_3$ and C($\underline{C}$H$_3$)$_3$), 113.87; 117.52; 123.24; 124.13 (Cp ring carbons), 135.56 (CF), 136.61 (CF), 137.25; 137.82; 138.10; 138.57; 139.48; 139.68 (Cp ring carbons), 147.74 (CF), 150.13 (Cp ring carbon). **^{11}B NMR** (δ/ppm): –16.95 (quintet, BH$_4$, $^1J_{BH}$ = 82 Hz), –15.55 (s, BAr$_4$). **^{11}B{^{1}H} NMR** (δ/ppm): –17.41; –16.96 (2 s, BH$_4$), –15.55 (s, BAr$_4$). ^{19}F{^{1}H} NMR (δ/ppm): –165.91 (8F, m, *m*-ArF), –162.24 (4F, m, *p*-ArF), –131.22 (8F, m, *o*-ArF).

- **Synthesis of [{Dy(Cp^{ttt})(Cp^{Me4t})}$_2$(μ-κ^2:κ^2-BH$_4$)][B(C$_6$F$_5$)$_4$] ([3$_{Dy}$][B(C$_6$F$_5$)$_4$])**

Compound [**3$_{Dy}$**][B(C$_6$F$_5$)$_4$] was synthesised via the same procedure as for [**3$_Y$**][B(C$_6$F$_5$)$_4$], using **2$_{Dy}$** (260 mg, 0.44 mmol) and [CPh$_3$][B(C$_6$F$_5$)$_4$] (203 mg, 0.22 mmol), and isolated as a yellow powder (309 mg, 76%). Yellow crystals suitable for x-ray crystallography were grown from a saturated diethyl ether solution stored at –35 °C for 72 hours.

Elemental analysis (%), found (calculated) for $C_{84}H_{104}B_2Dy_2F_{20}$: C 54.96 (54.82); H 5.72 (5.70).

FTIR ($\tilde{\upsilon}$/cm^{-1}): 3050-2700 (m, br, C-H), 2400-2150 (s, B-H$_B$).

- **Synthesis of [{Lu(Cp^{ttt})(Cp^{Me4t})}$_2$(μ-κ^2:κ^2-BH$_4$)][B(C$_6$F$_5$)$_4$] ([3$_{Lu}$][B(C$_6$F$_5$)$_4$])**

Compound [**3$_{Lu}$**][B(C$_6$F$_5$)$_4$] was synthesised via the same procedure as for [**3$_Y$**][B(C$_6$F$_5$)$_4$], using **2$_{Lu}$** (126 mg, 0.20 mmol) and [CPh$_3$][B(C$_6$F$_5$)$_4$] (96 mg, 0.10 mmol), and isolated as a white powder (60 mg, 31%). Colourless crystals of **3$_{Lu}$** were grown from a saturated diethyl ether solution stored at –35 °C for 72 hours.

Elemental analysis (%), found (calculated) for $C_{84}H_{104}B_2F_{20}Lu_2$: C 53.99 (54.09); H 5.66 (5.62).

FTIR ($\tilde{\upsilon}$/cm^{-1}): 3050-2700 (m, br, C-H), 2400-2150 (s, B-H$_B$).

^{1}H NMR (δ/ppm): 1.18 (36 H, s, ^{t}Bu), 1.27 (18H, br s, ^{t}Bu), 1.50 (18H, br s, ^{t}Bu), 1.73 (6H, br s, Me), 2.33; 2.39 (18H, 2 br s, Me), 6.47; 6.72 (4H, 2 br s, CpH). **^{13}C{^{1}H} NMR** (δ/ppm): 12.47 (Me), 13.52 (Me), 16.08 (Me), 17.81 (Me), 32.06; 32.65; 33.31; 33.63; 35.00; 35.43 ($\underline{C}$Me$_3$ and C($\underline{C}$H$_3$)$_3$), 113.38; 117.94; 124.31 (Cp ring carbons), 135.61 (CF), 137.46 (CF), 138.19; 139.17; 139.87 (Cp ring carbons), 147.73 (CF), 150.08 (Cp ring carbon). **^{11}B NMR** (δ/ppm): –17.40 (p, BH$_4$, $^1J_{BH}$ = 82 Hz), –15.55 (s, BAr$_4$). **^{11}B{^{1}H} NMR** (δ/ppm): –17.40 (s, BH$_4$), –15.55 (s, BAr$_4$). **^{19}F{^{1}H} NMR** (δ/ppm): –165.92 (8F, m, *m*-ArF), –162.26 (4F, m, *p*-ArF), –131.20 (8F, m, *o*-ArF).

- **Synthesis of magnetically dilute [3$_{Dy}$][B(C$_6$F$_5$)$_4$] in a matrix of [3$_{Dy}$][B(C$_6$F$_5$)$_4$] (Dy@3$_Y$).**

The magnetically dilute compound **Dy@3$_Y$** was synthesised using the method described above for [**3$_M$**][B(C$_6$F$_5$)$_4$] by adding a solution of **2$_Y$** (248 mg, 0.48 mmol) and **2$_{Dy}$** (50 mg, 0.09 mmol) in hexane (8 mL) to [CPh$_3$][B(C$_6$F$_5$)$_4$] (262 mg, 0.28 mmol). Pale yellow crystals of **Dy@3$_Y$** were isolated (201 mg, 41%) and were shown by ICP-MS to contain a Y:Dy molar ratio of 83.7:16.3.

Elemental analysis (%), found (calculated) for $C_{84}H_{104}B_2Dy_{0.1633}F_{20}Y_{0.8367}$: C 59.01 (58.82); H 6.05 (6.11).

X-Ray Crystallography

Single-crystal X-ray diffraction measurements were carried out on an Agilent Gemini Ultra diffractometer using CuK$_\alpha$ radiation (λ = 1.54184 Å) at 100 K. Structures were solved in Olex2 with SHELXT using intrinsic phasing and were refined with SHELXL using least squares minimisation.[5–7] Anisotropic thermal parameters were used for non-hydrogen atoms and isotropic parameters for hydrogen atoms. Hydrogen atoms on carbons were added geometrically and refined using a riding model. The hydrogen atoms of borohydrides were located based on residual electron density and were refined freely. Solvent masking was used in the refinement of [$\mathbf{3}_{\mathbf{Y}}$][$B(C_6F_5)_4$] and [$\mathbf{3}_{\mathbf{Dy}}$][$B(C_6F_5)_4$] due to the presence of highly disordered solvent molecules in the lattice (toluene and heptane for the yttrium version and diethyl ether and hexane for dysprosium).

Table S1. Crystal data and structure refinement parameters for $\mathbf{1}_{\mathbf{Y}}$, $\mathbf{1}_{\mathbf{Dy}}$, and $\mathbf{1}_{\mathbf{Lu}}$.

	$\mathbf{1}_{\mathbf{Y}}$	$\mathbf{1}_{\mathbf{Dy}}$	$\mathbf{1}_{\mathbf{Lu}}$
CCDC ref. code	2149639	2149640	2149641
Empirical formula	$C_{21}H_{45}B_2OY$	$C_{21}H_{45}B_2DyO$	$C_{21}H_{45}B_2LuO$
Formula weight	424.11	497.69	510.16
Crystal system	monoclinic	monoclinic	monoclinic
Space group	$P2_1/c$	$P2_1/c$	$P2_1/c$
a (Å)	18.1896(9)	18.1666(5)	18.0768(10)
b (Å)	8.9828(4)	8.9870(2)	8.9778(6)
c (Å)	16.4067(6)	16.4031(3)	16.4090(7)
α (°)	90	90	90
β (°)	113.891(5)	113.855(3)	113.876(6)
γ (°)	90	90	90
V (Å^3)	2451.1(2)	2449.24(11)	2435.1(3)
Z	4	4	4
ρ_{calc} (g cm^{-3})	1.149	1.350	1.392
F (000)	912.0	1020.0	1040.0
Reflections collected	7091	17872	16195
Independent reflections	4341	4727	4600
R_{int} (%)	4.07	5.55	5.62
GOF on F^2	1.058	1.038	1.039
R_1[a]	0.0453	0.0379	0.0369
wR_2[b]	0.1101	0.0999	0.0820

[a] $R_1[I>2\sigma(I)] = \sum||F_o|-|F_c||/\sum|F_o|$; [b] $wR_2[\text{all data}] = [\sum\{w(F_o^2-F_c^2)^2\}/\sum\{w(F_o^2)^2\}]^{1/2}$

Table S2. Crystal data and structure refinement parameters for $\mathbf{2_{Y}}$, $\mathbf{2_{Dy}}$, and $\mathbf{2_{Lu}}$.

	$\mathbf{2_{Y}}$	$\mathbf{2_{Dy}}$	$\mathbf{2_{Lu}}$
CCDC ref. code	2149642	2149643	2149644
Empirical formula	$C_{30}H_{54}BY$	$C_{30}H_{54}BDy$	$C_{30}H_{54}BLu$
Formula weight	514.45	588.04	600.51
Crystal system	monoclinic	monoclinic	monoclinic
Space group	*Cc*	$P2_1/c$	*Cc*
a (Å)	8.71106(9)	8.70020(10)	8.70720(10)
b (Å)	21.4885(2)	17.3510(3)	21.4077(3)
c (Å)	15.63677(16)	19.3871(3)	15.6265(3)
α (°)	90	90	90
β (°)	93.8167(10)	98.056(2)	93.807(2)
γ (°)	90	90	90
V (Å^3)	2920.52(5)	2897.74(8)	2906.37(8)
Z	4	4	4
ρ_{calc} (gcm^{-3})	1.170	1.348	1.372
F (000)	1112.0	1220.0	1240.0
Reflections collected	49455	10413	10412
Independent reflections	5041	5500	4251
R_{int} (%)	5.45	2.36	3.57
GOF on F^2	1.023	1.020	1.073
R_1[a]	0.0206	0.0268	0.0248
wR_2[b]	0.0506	0.0680	0.0645

[a] $R_1[I>2\sigma(I)] = \sum||F_o|-|F_c||/\sum|F_o|$; [b] wR_2[all data] = $[\sum\{w(F_o^2-F_c^2)^2\}/\sum\{w(F_o^2)^2\}]^{1/2}$

Table S3. Crystal data and structure refinement parameters for [**3**$_{Y}$][B(C_6F_5)$_4$], [**3**$_{Dy}$][B(C_6F_5)$_4$], and [**3**$_{Lu}$][B(C_6F_5)$_4$].

	[**3**$_{Y}$][B(C_6F_5)$_4$]	[**3**$_{Dy}$][B(C_6F_5)$_4$]	[**3**$_{Lu}$][B(C_6F_5)$_4$]
CCDC ref. code	2149645	2149646	2149647
Empirical formula	$C_{84}H_{104}B_2F_{20}Y_2$	$C_{84}H_{104}B_2Dy_2F_{20}$	$C_{87}H_{110}B_2F_{20}Lu_2$
Formula weight	1693.11	1840.29	1907.30
Crystal system	monoclinic	monoclinic	monoclinic
Space group	$P2_1/c$	$P2_1/c$	$P2_1/c$
a (Å)	15.8093(3)	15.7950(3)	15.7485(2)
b (Å)	36.2308(4)	36.1238(7)	35.8404(6)
c (Å)	17.2075(2)	17.2459(5)	17.2223(2)
α (°)	90	90	90
β (°)	110.082(2)	110.057(3)	109.778(2)
γ (°)	90	90	90
V (Å^3)	9256.9(3)	9243.3(4)	9147.4(2)
Z	4	4	4
ρ_{calc} (gcm^{-3})	1.215	1.322	1.385
F (000)	3504.0	3720.0	3856.0
Reflections collected	69334	36167	48281
Independent reflections	17712	16281	16319
R_{int} (%)	5.24	7.74	9.18
GOF on F^2	1.041	1.006	0.960
R_1 [a]	0.0472	0.0595	0.0533
wR_2 [b]	0.1077	0.1416	0.1299

[a] $R_1[I>2\sigma(I)] = \sum||F_o|-|F_c||/\sum|F_o|$; [b] wR_2[all data] $= [\sum\{w(F_o^2-F_c^2)^2\}/\sum\{w(F_o^2)^2\}]^{1/2}$

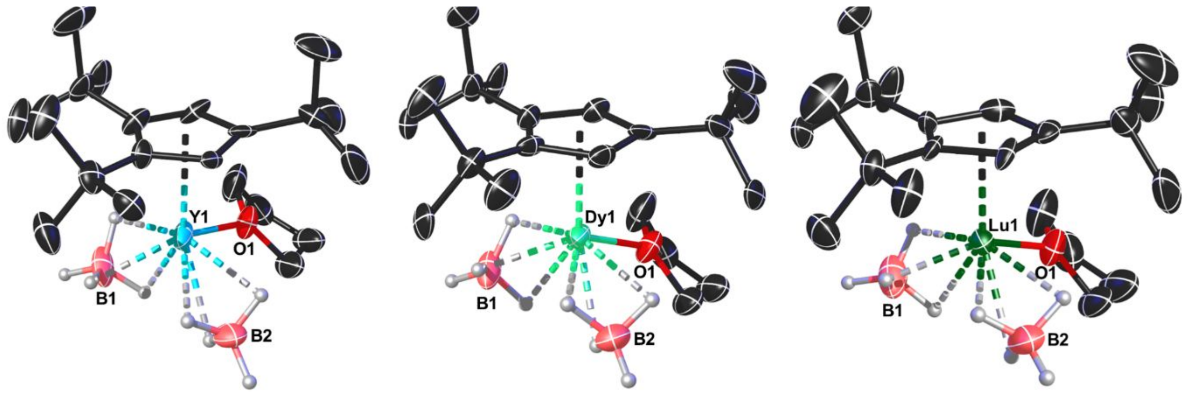

Figure S1. Thermal ellipsoid representations (50% probability) of $\mathbf{1}_{Y}$ (left), $\mathbf{1}_{Dy}$ (middle) and $\mathbf{1}_{Lu}$ (right). For clarity, only hydrogens bonded to boron are shown.

Table S4. Selected bond lengths (Å) for $\mathbf{1}_{Y}$, $\mathbf{1}_{Dy}$, and $\mathbf{1}_{Lu}$.[a]

	$\mathbf{1}_{Y}$	$\mathbf{1}_{Dy}$	$\mathbf{1}_{Lu}$
M-C	Y1-C1: 2.632(12)<br>Y1-C2: 2.667(14)<br>Y1-C3: 2.741(11)<br>Y1-C4: 2.649(10)<br>Y1-C5: 2.587(11)<br>Y1-C1A: 2.678(13)<br>Y1-C2A: 2.551(12)<br>Y1-C3A: 2.571(13)<br>Y1-C4A: 2.683(12)<br>Y1-C5A: 2.697(13)	Dy1-C1: 2.660(9)<br>Dy1-C2: 2.668(12)<br>Dy1-C3: 2.720(12)<br>Dy1-C4: 2.662(11)<br>Dy1-C5: 2.598(10)<br>Dy1-C1A: 2.663(11)<br>Dy1-C2A: 2.562(10)<br>Dy1-C3A: 2.631(11)<br>Dy1-C4A: 2.706(12)<br>Dy1-C5A: 2.674(11)	Lu1-C1: 2.448(15)<br>Lu1-C2: 2.439(19)<br>Lu1-C3: 2.610(18)<br>Lu1-C4: 2.63(2)<br>Lu1-C5: 2.554(16)<br>Lu1A-C1A: 2.616(16)<br>Lu1A-C2A: 2.603(16)<br>Lu1A-C3A: 2.583(16)<br>Lu1A-C4A: 2.625(16)<br>Lu1A-C5A: 2.618(16)
M-Cp^{ttt}_{cent}	Part A: 2.365(6)<br>Part B: 2.348(6)	A: 2.369(6)<br>B: 2.357(6)	A: 2.248(10)<br>B: 2.315(8)
M-B	Y1-B1: 2.500(7)<br>Y1-B2: 2.512(5)	Dy1-B1: 2.507(6)<br>Dy1-B2: 2.504(5)	Lu1-B1: 2.661(12)<br>Lu1-B2: 2.437(7)<br>Lu1A-B1: 2.348(11)<br>Lu1A-B2: 2.462(7)
M-O	2.316(4)	2.334(4)	Lu1-O1: 2.004(9)<br>Lu1A-O1: 2.385(7)

[a] The Cp^{ttt} ligand is disordered over two positions in all three complexes, with relative occupancies 0.53:0.47 in $\mathbf{1}_{Y}$, 0.50:0.50 in $\mathbf{1}_{Dy}$, and 0.51:0.49 in $\mathbf{1}_{Lu}$; the two associated centroid positions are referred as parts A and B in the table. In $\mathbf{1}_{Lu}$, the lutetium atom is also disordered over two positions with relative occupancies 0.69:0.31.

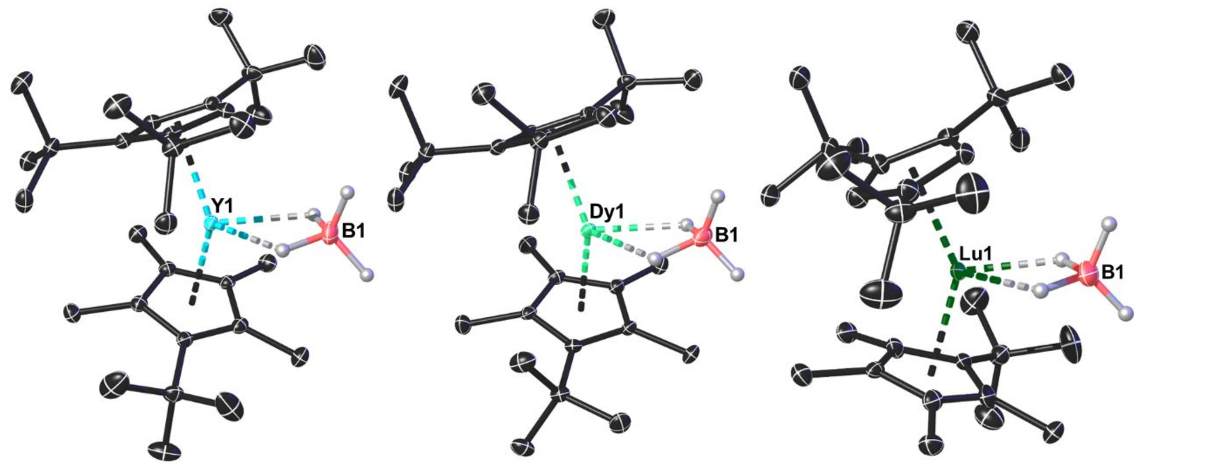

Figure S2. Thermal ellipsoid representations (50% probability) of $\mathbf{2_Y}$ (left), $\mathbf{2_{Dy}}$ (middle) and $\mathbf{2_{Lu}}$ (right). For clarity, only hydrogens bonded to boron are shown.

Table S5. Selected bond lengths (Å) and angles (°) for $\mathbf{2_Y}$, $\mathbf{2_{Dy}}$, and $\mathbf{2_{Lu}}$.

	$\mathbf{2_Y}$	$\mathbf{2_{Dy}}$	$\mathbf{2_{Lu}}$
M-C (Cp^{ttt})	Y1-C1: 2.630(3)<br>Y1-C2: 2.676(3)<br>Y1-C3: 2.674(3)<br>Y1-C4: 2.611(3)<br>Y1-C5: 2.632(3)	Dy1-C1: 2.629(2)<br>Dy1-C2: 2.638(3)<br>Dy1-C3: 2.704(2)<br>Dy1-C4: 2.709(3)<br>Dy1-C5: 2.626(3)	Lu1-C1: 2.634(6)<br>Lu1-C2: 2.635(6)<br>Lu1-C3: 2.582(6)<br>Lu1-C4: 2.588(6)<br>Lu1-C5: 2.560(6)
M-C (Cp^{Me4t})	Y1-C18: 2.657(3)<br>Y1-C19: 2.666(3)<br>Y1-C20: 2.675(3)<br>Y1-C21: 2.641(3)<br>Y1-C22: 2.628(3)	Dy1-C18: 2.655(3)<br>Dy1-C19: 2.646(3)<br>Dy1-C20: 2.651(3)<br>Dy1-C21: 2.650(3)<br>Dy1-C22: 2.653(3)	Lu1-C18: 2.625(6)<br>Lu1-C19: 2.625(6)<br>Lu1-C20: 2.571(6)<br>Lu1-C21: 2.574(6)<br>Lu1-C22: 2.628(6)
M-Cp^{ttt}_{cent}	2.3522(14)	2.3692(11)	2.300(3)
M-Cp^{Me4t}_{cent}	2.3609(14)	2.3578(12)	2.307(3)
M-B	2.700(4)	2.713(3)	2.627(8)
Cp^{ttt}_{cent}-M-Cp^{Me4t}_{cent}	142.68(5)	143.44(4)	143.33(10)

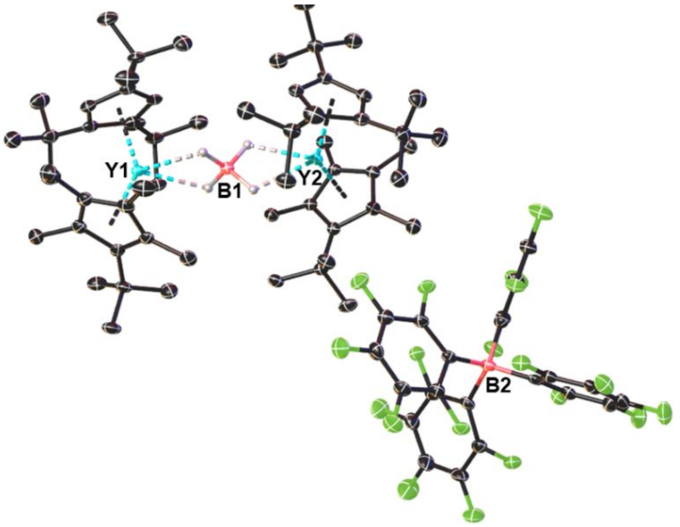

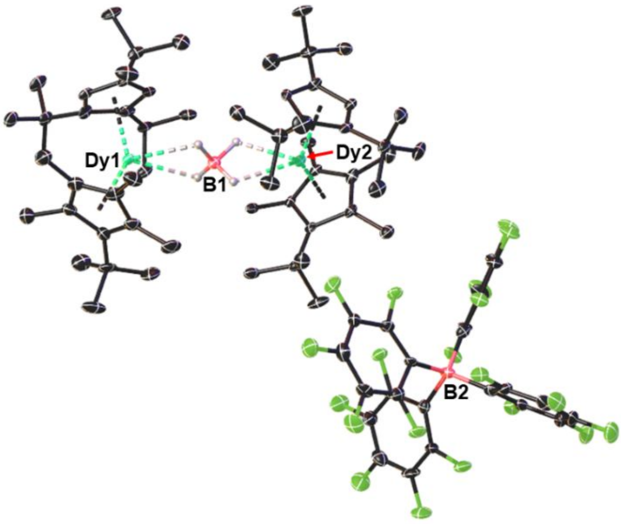

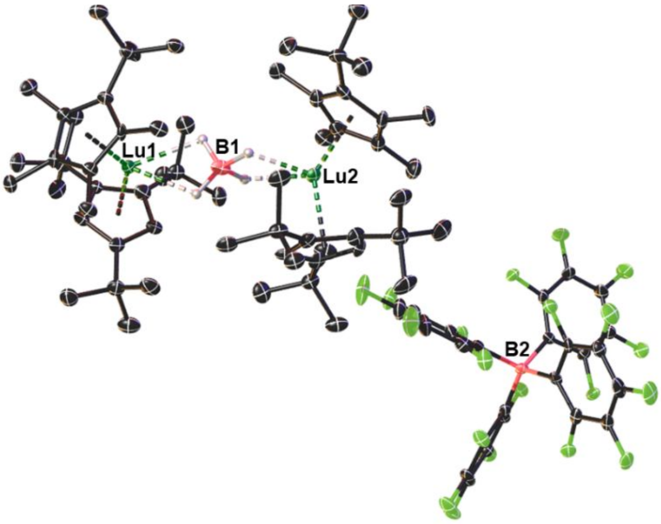

Figure S3. Thermal ellipsoid representations (50% probability) of [$\mathbf{3}_{\mathbf{Y}}$][$B(C_6F_5)_4$] (top), [$\mathbf{3}_{\mathbf{Dy}}$][$B(C_6F_5)_4$] (middle) and [$\mathbf{3}_{\mathbf{Lu}}$][$B(C_6F_5)_4$] (bottom). For clarity, only hydrogens bonded to boron are shown.

Table S6. Selected bond lengths (Å) and angles (°) for $\mathbf{3_{Y}}$, $\mathbf{3_{Dy}}$, and $\mathbf{3_{Lu}}$.

	$\mathbf{3_{Y}}$	$\mathbf{3_{Dy}}$	$\mathbf{3_{Lu}}$
**M-C (Cp^{ttt})**	Y1-C1: 2.691(3)<br>Y1-C2: 2.616(3)<br>Y1-C3: 2.614(3)<br>Y1-C4: 2.605(3)<br>Y1-C5: 2.676(3)<br>Y2-C31: 2.595(3)<br>Y2-C32: 2.639(3)<br>Y2-C33: 2.625(3)<br>Y2-C34: 2.656(3)<br>Y2-C35: 2.641(3)	Dy1-C1: 2.684(6)<br>Dy1-C2: 2.688(6)<br>Dy1-C3: 2.622(6)<br>Dy1-C4: 2.617(6)<br>Dy1-C5: 2.610(6)<br>Dy2-C31: 2.672(6)<br>Dy2-C32: 2.645(6)<br>Dy2-C33: 2.598(6)<br>Dy2-C34: 2.627(6)<br>Dy2-C35: 2.621(6)	Lu1-C1: 2.661(7)<br>Lu1-C2: 2.642(6)<br>Lu1-C3: 2.547(6)<br>Lu1-C4: 2.572(6)<br>Lu1-C5: 2.566(7)<br>Lu2-C31: 2.590(6)<br>Lu2-C32: 2.619(6)<br>Lu2-C33: 2.550(6)<br>Lu2-C34: 2.587(7)<br>Lu2-C35: 2.550(6)
**M-C (Cp^{Me4t})**	Y1-C18: 2.636(3)<br>Y1-C19: 2.662(3)<br>Y1-C20: 2.647(3)<br>Y1-C21: 2.613(3)<br>Y1-C22: 2.624(3)<br>Y2-C48: 2.652(3)<br>Y2-C49: 2.664(3)<br>Y2-C50: 2.621(3)<br>Y2-C51: 2.625(3)<br>Y2-C52: 2.640(3)	Dy1-C18: 2.624(6)<br>Dy1-C19: 2.657(6)<br>Dy1-C20: 2.659(6)<br>Dy1-C21: 2.635(6)<br>Dy1-C22: 2.627(6)<br>Dy2-C48: 2.644(7)<br>Dy2-C49: 2.658(6)<br>Dy2-C50: 2.636(7)<br>Dy2-C51: 2.616(6)<br>Dy2-C52: 2.616(7)	Lu1-C18: 2.580(7)<br>Lu1-C19: 2.569(6)<br>Lu1-C20: 2.579(6)<br>Lu1-C21: 2.613(6)<br>Lu1-C22: 2.597(6)<br>Lu2-C48: 2.616(6)<br>Lu2-C49: 2.571(6)<br>Lu2-C50: 2.564(6)<br>Lu2-C51: 2.587(6)<br>Lu2-C52: 2.608(6)
**M-Cp$^{ttt}_{cent}$**	2.3468(16) (Y1)<br>2.3367(13) (Y2)	2.351(3) (Dy1)<br>2.340(3) (Dy2)	2.298(3) (Lu1)<br>2.277(3) (Lu2)
**M-Cp$^{Me4t}_{cent}$**	2.3399(15) (Y1)<br>2.3452(13) (Y2)	2.346(3) (Dy1)<br>2.343(3) (Dy2)	2.284(3) (Lu1)<br>2.288(3) (Lu2)
**M···M**	5.6877(6)	5.7097(6)	5.6048(6)
**M(1)-B(1)**<br>**M(2)-B(1)**	2.853(4)<br>2.839(4)	2.855(7)<br>2.857(7)	2.801(9)<br>2.808(9)
**Cp$^{ttt}_{cent}$-M-Cp$^{Me4t}_{cent}$**	139.12(6) (Y1)<br>141.156(6) (Y2)	138.94(11) (Dy1)<br>141.23(12) (Dy2)	139.00(11) (Lu1)<br>140.71(12) (Lu2)
**M-B-M**	175.43(14)	176.5(3)	175.7(3)
**Torsion Angle**[a]	67.95(5)	68.70(10)	70.65(10)

[a] The angle of intersection between the two Cp$^{ttt}_{cent}$-M-Cp$^{Me4t}_{cent}$ planes.

IR Spectra and NMR Spectra

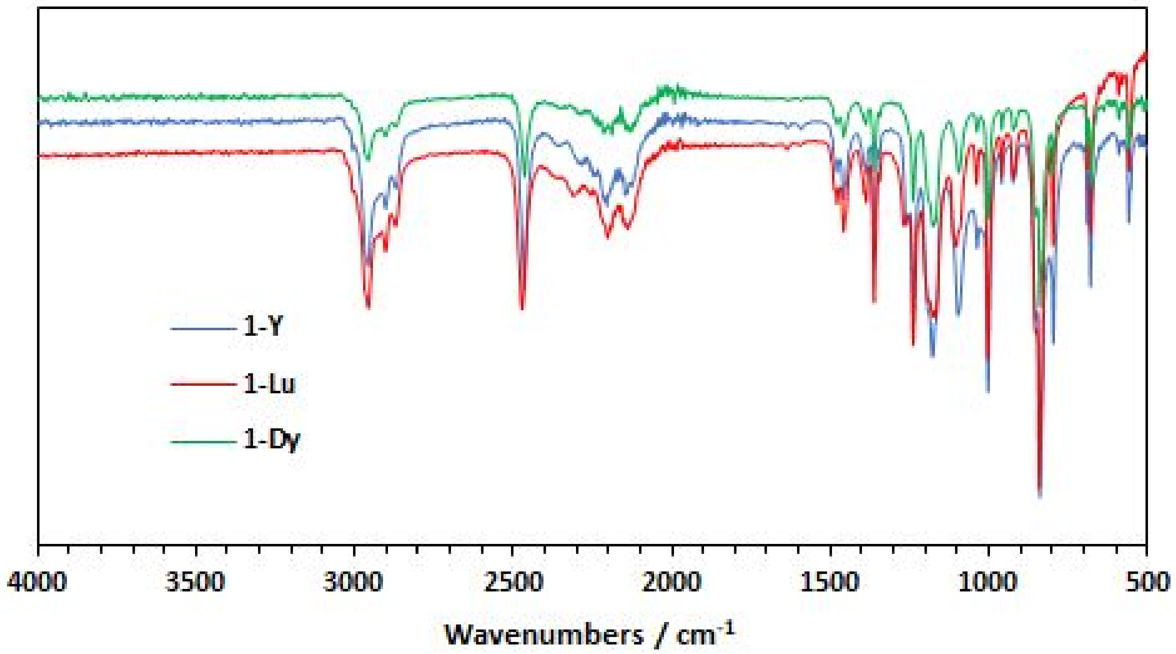

Figure S4. FTIR spectra of $\mathbf{1_M}$.

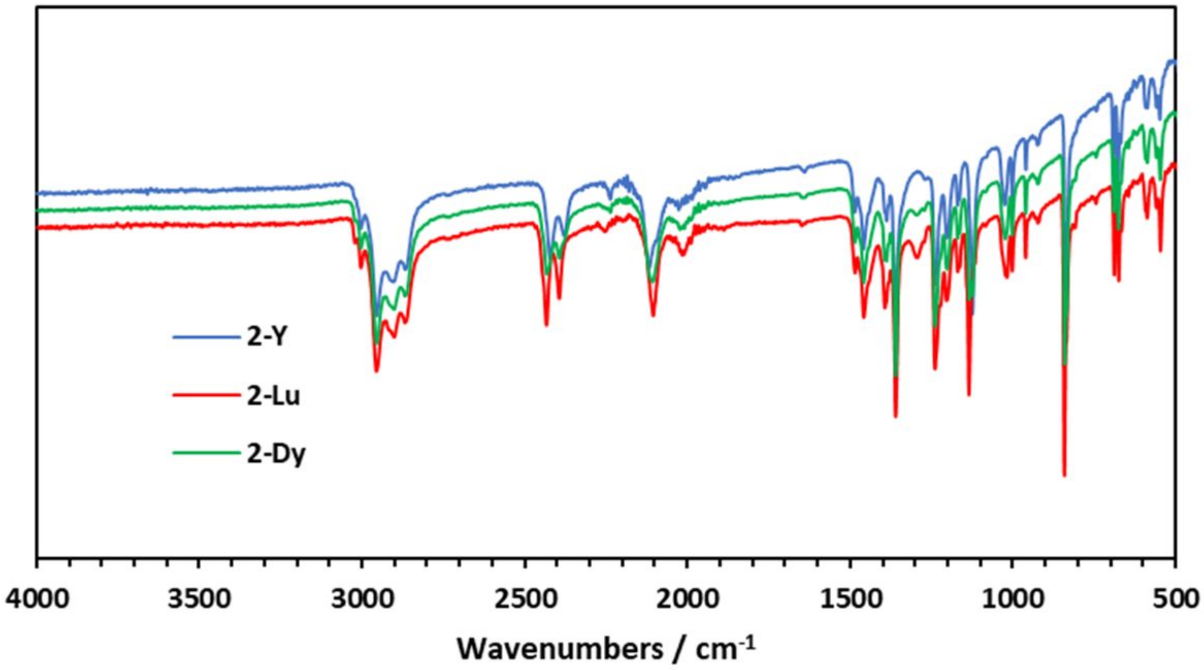

Figure S5. FTIR spectra of $\mathbf{2_M}$.

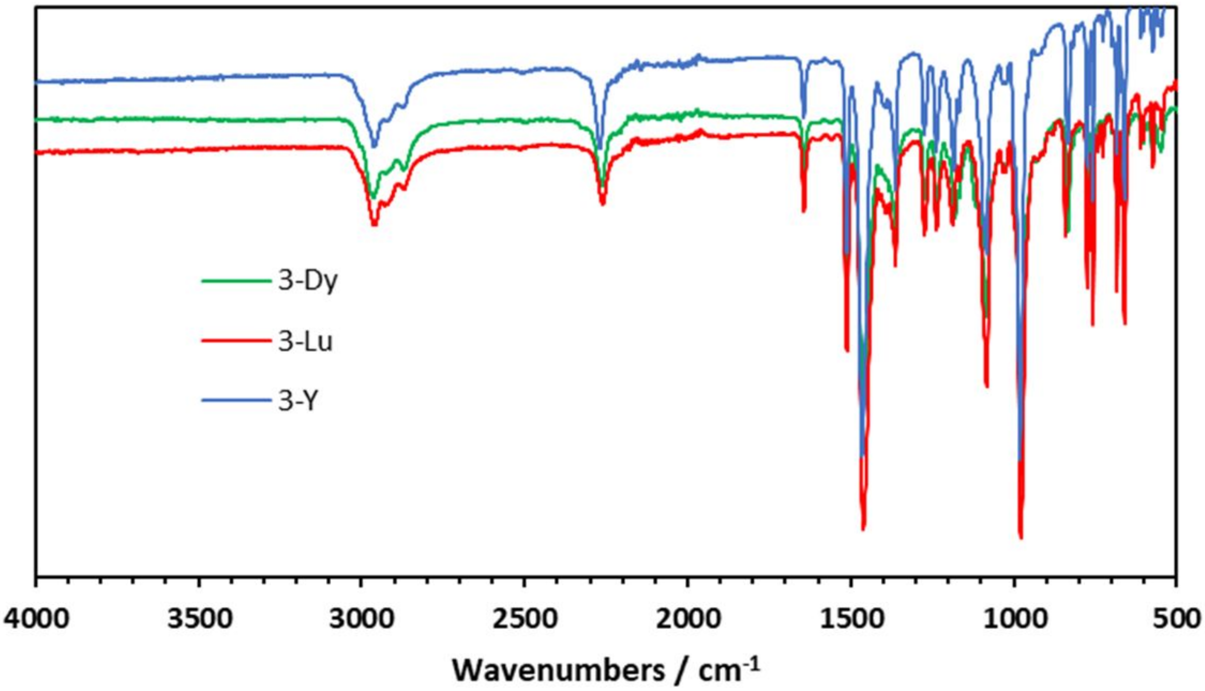

Figure S6. FTIR spectra of [$\mathbf{3_M}$][$B(C_6F_5)_4$].

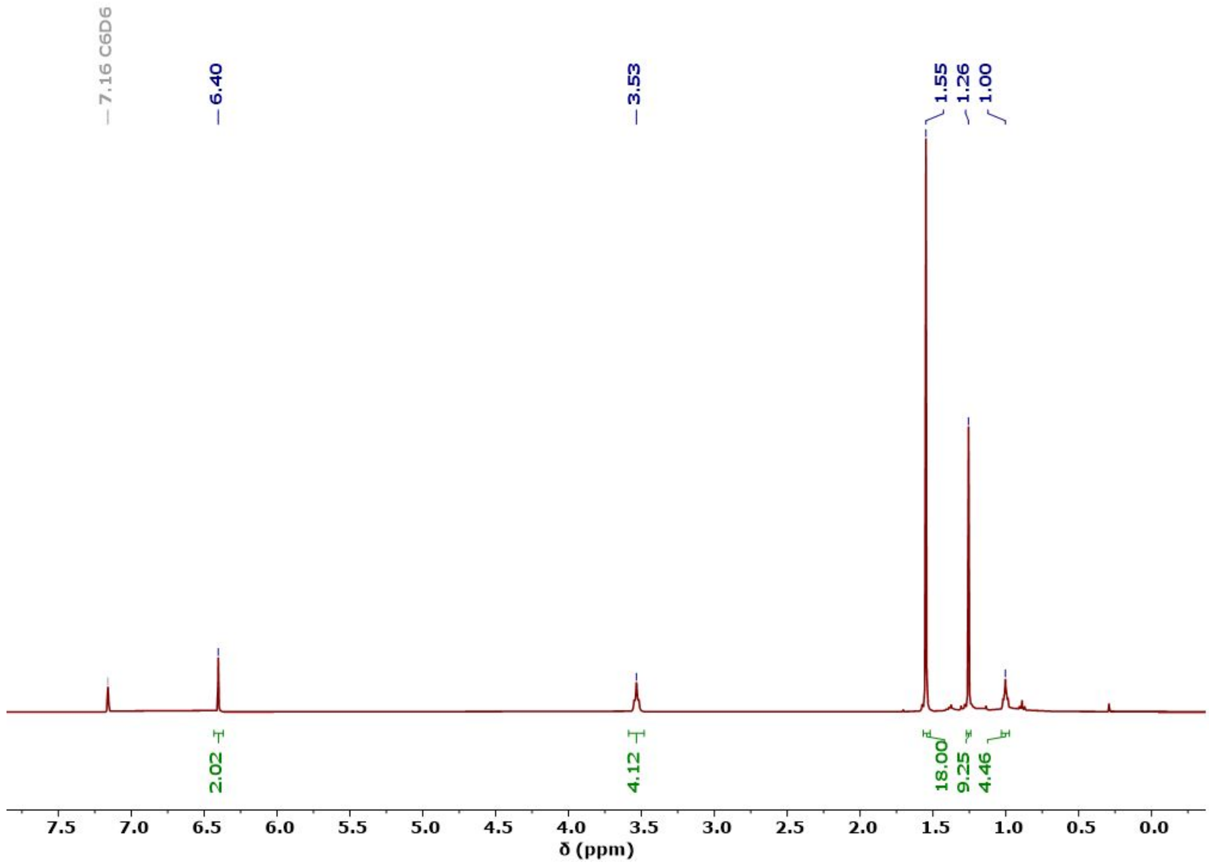

Figure S7. ^{1}H NMR spectrum of $\mathbf{1_Y}$ in benzene-D_6.

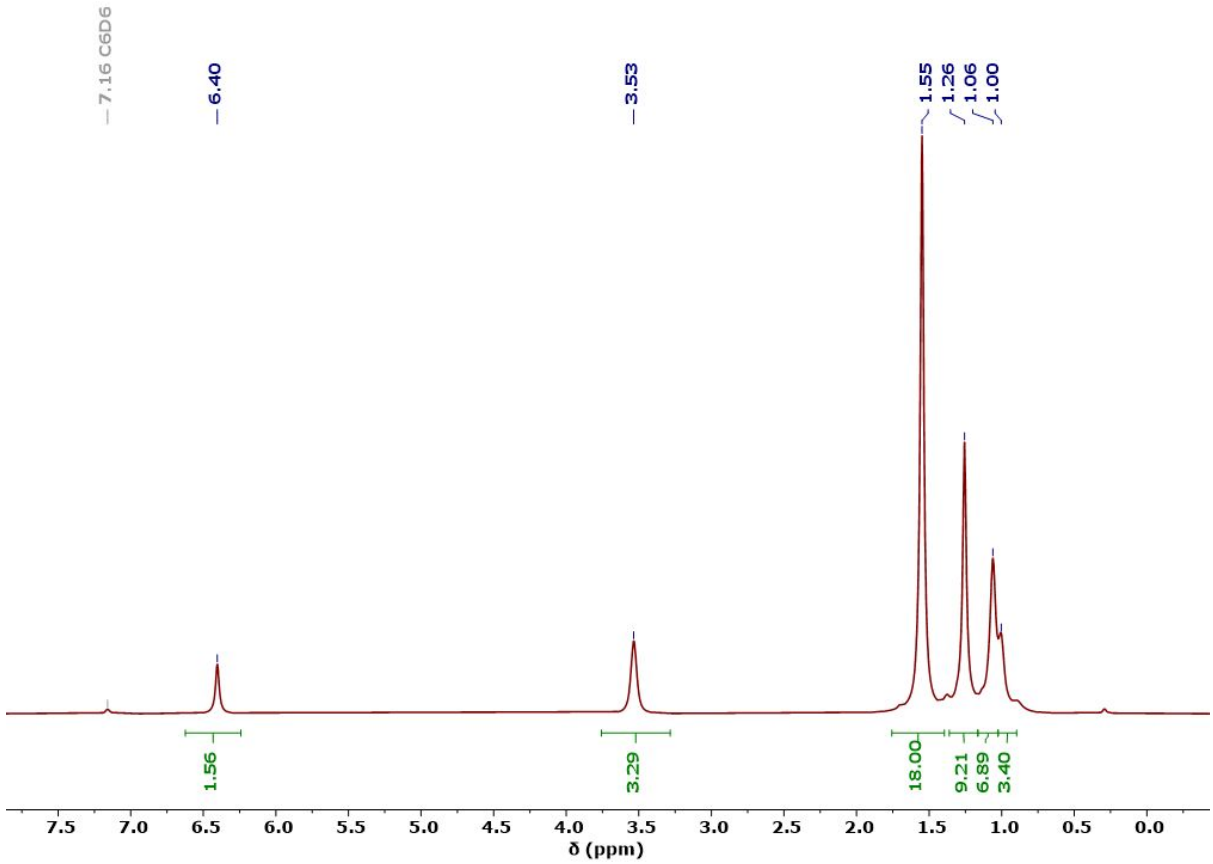

Figure S8. $^{1}H\{^{11}B\}$ NMR spectrum of $\mathbf{1_Y}$ in benzene-D_6.

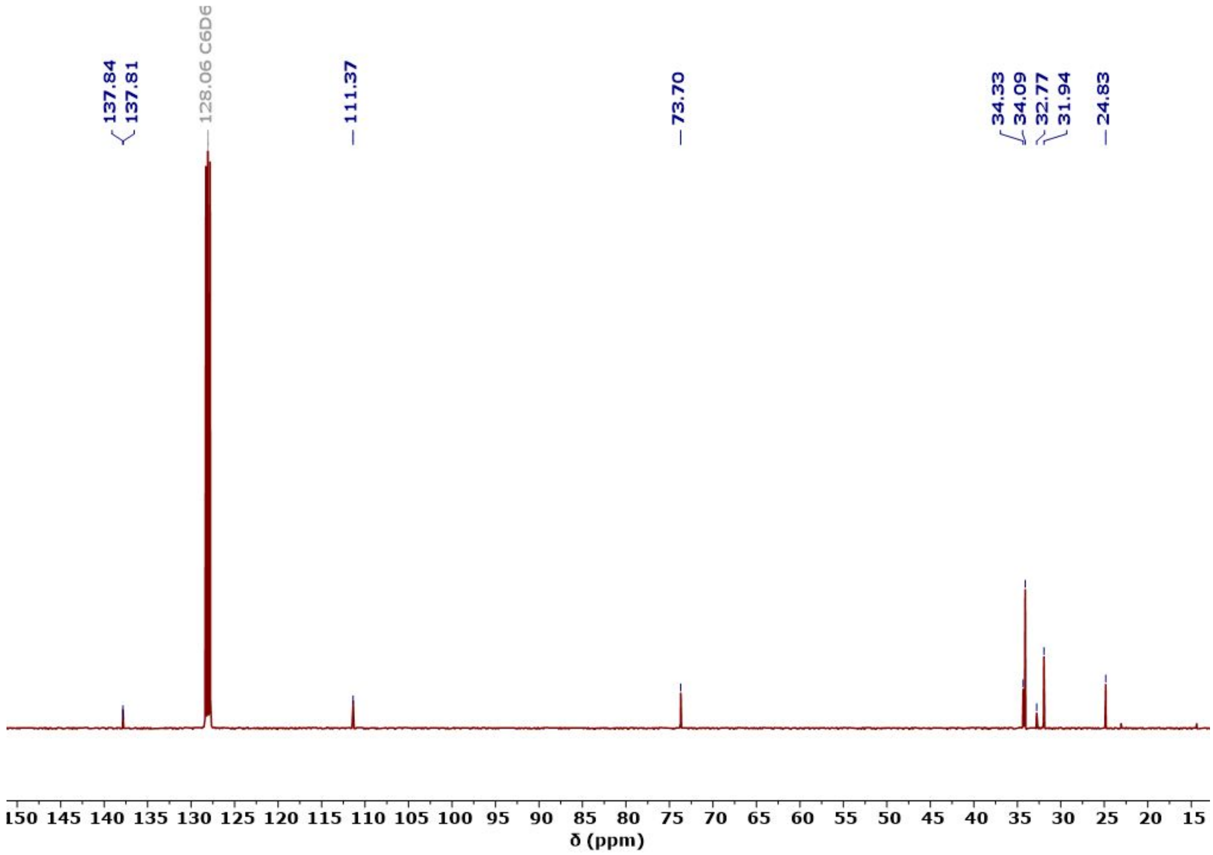

Figure S9. $^{13}C\{^1H\}$ NMR spectrum of $\mathbf{1_Y}$ in benzene-D_6.

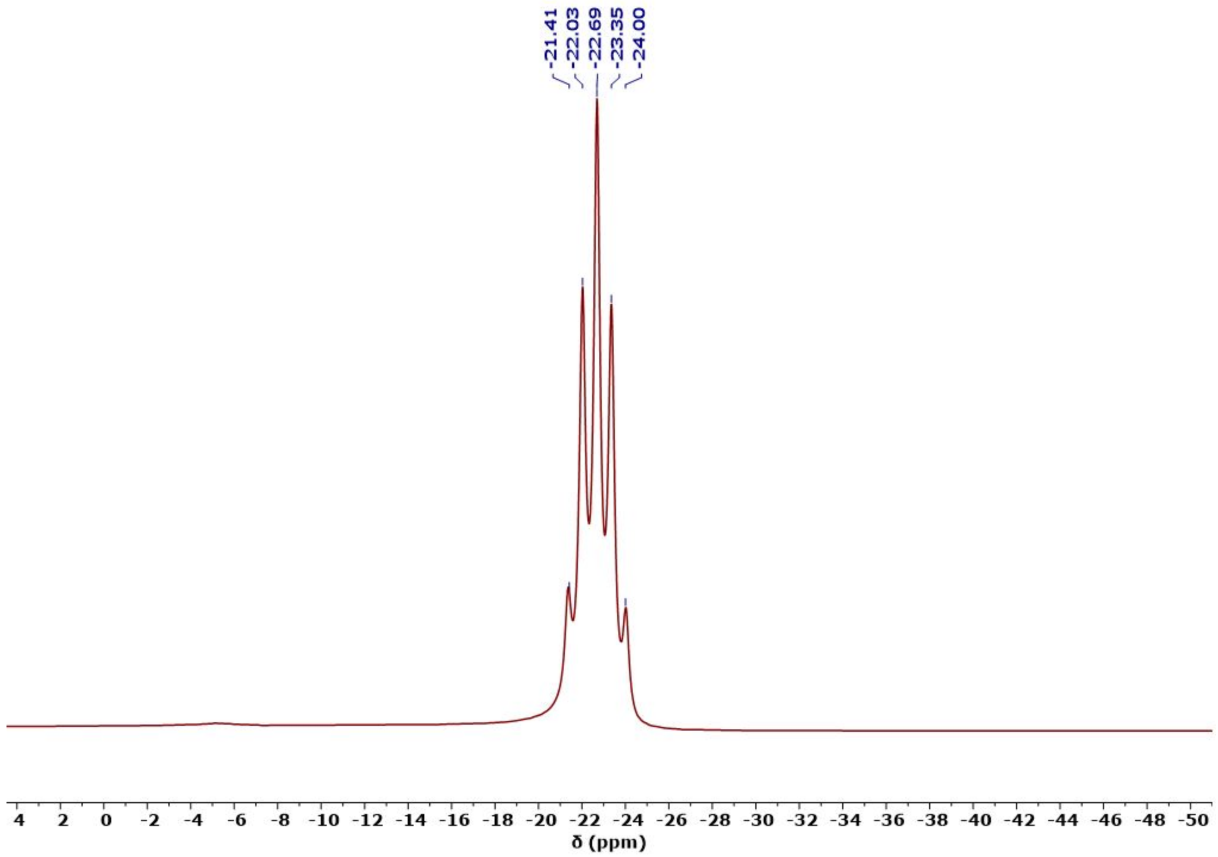

Figure S10. ^{11}B NMR spectrum of $\mathbf{1_{Y}}$ in benzene-D_6.

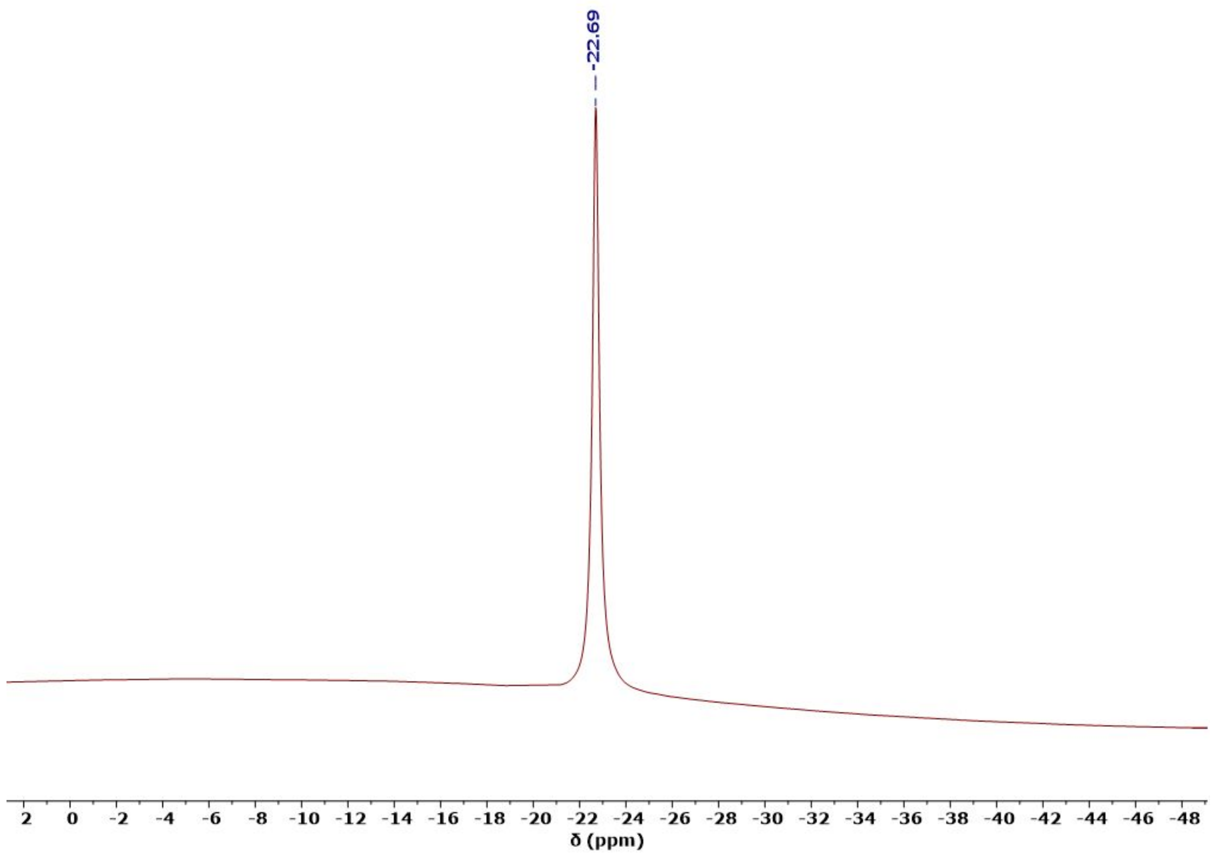

Figure S11. $^{11}B\{^{1}H\}$ NMR spectrum of $\mathbf{1_{Y}}$ in benzene-D_6.

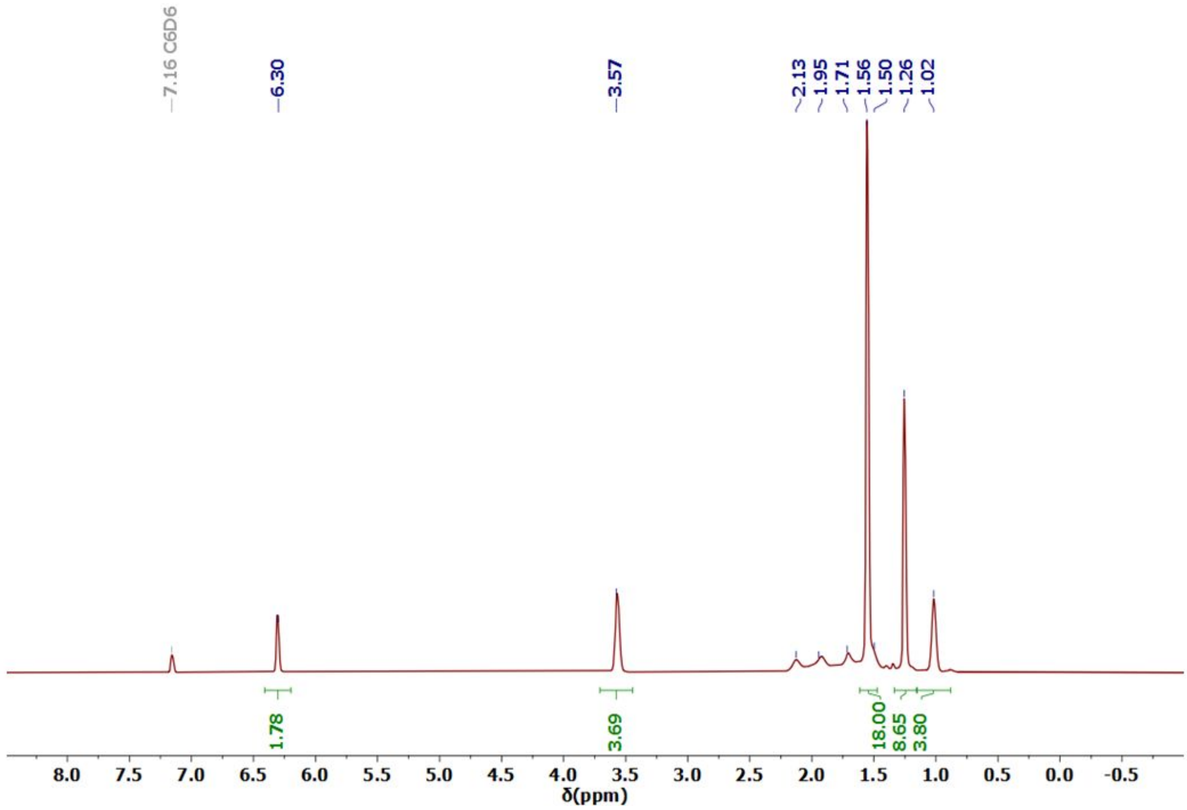

Figure S12. ^{1}H NMR spectrum of $\mathbf{1}_{\mathbf{Lu}}$ in benzene-D_6.

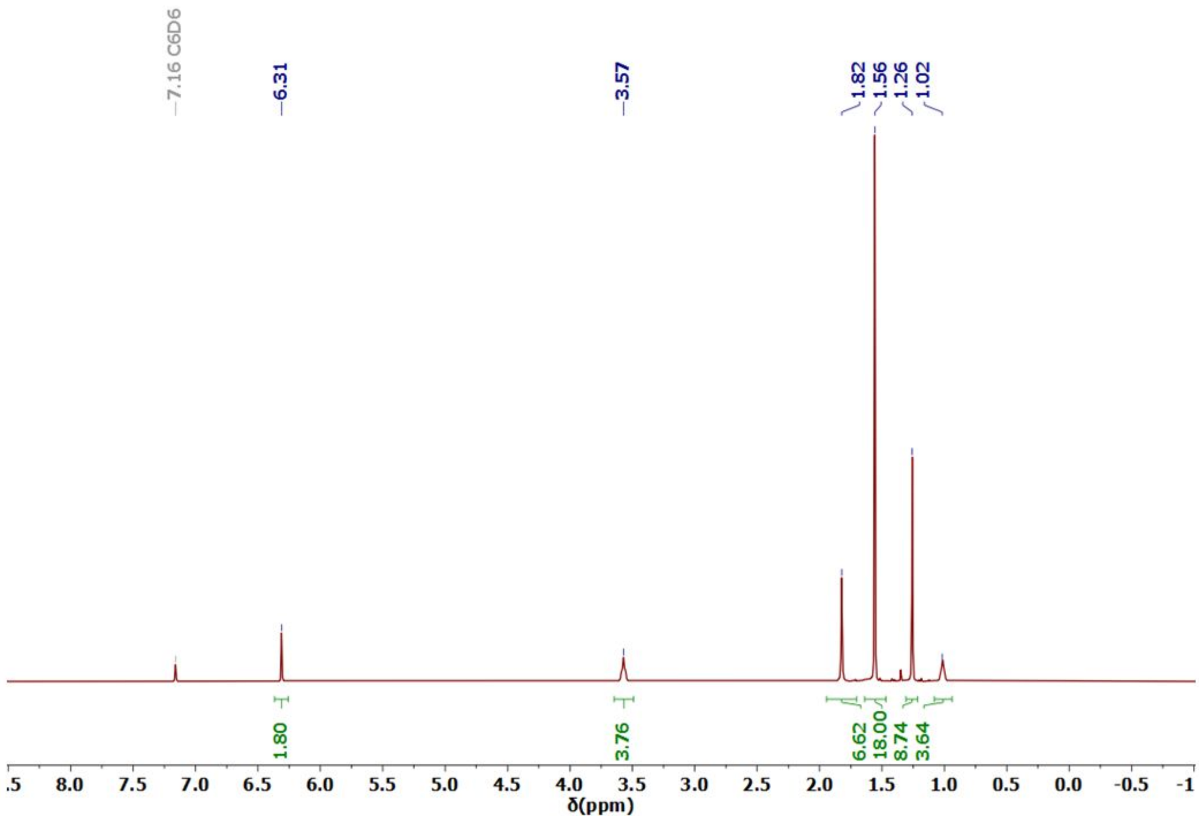

Figure S13. $^{1}H\{^{11}B\}$ NMR spectrum of $\mathbf{1_{Lu}}$ in benzene-D_6.

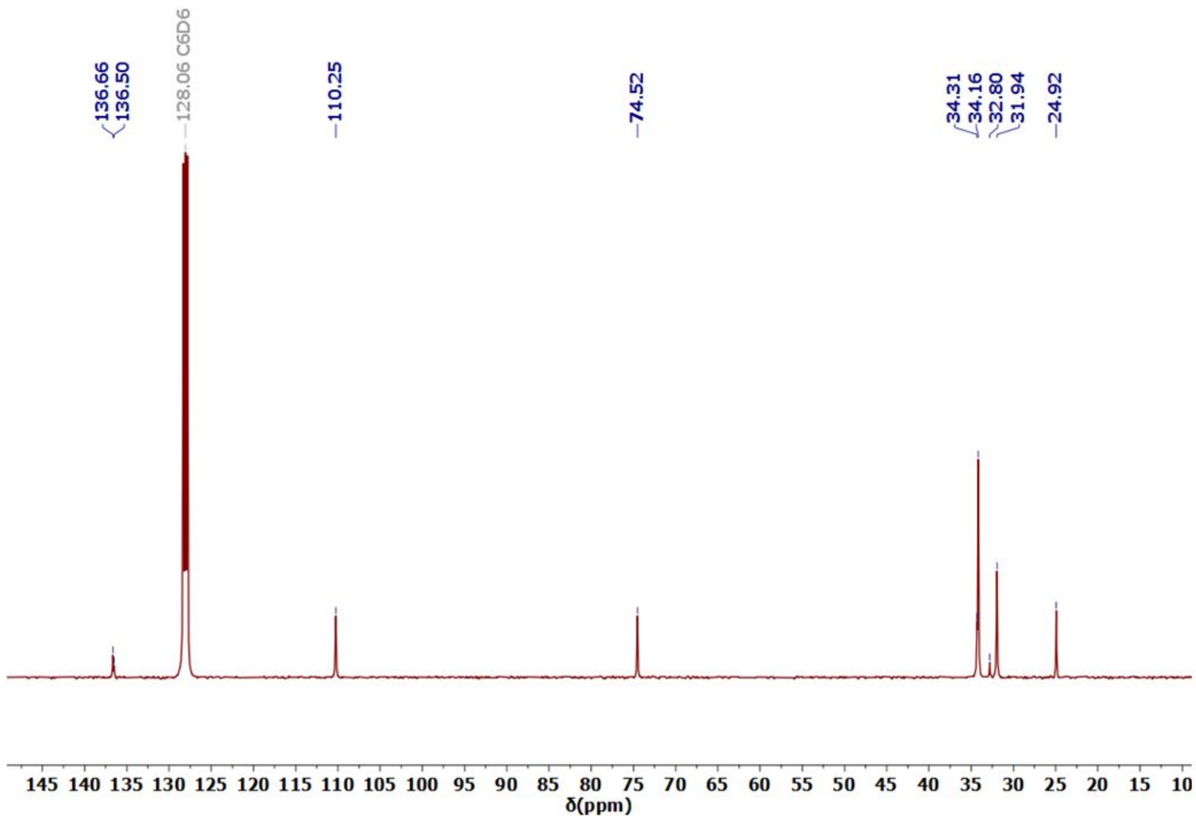

Figure S14. $^{13}C\{^{1}H\}$ NMR spectrum of $\mathbf{1}_{\mathbf{Lu}}$ in benzene-D_6.

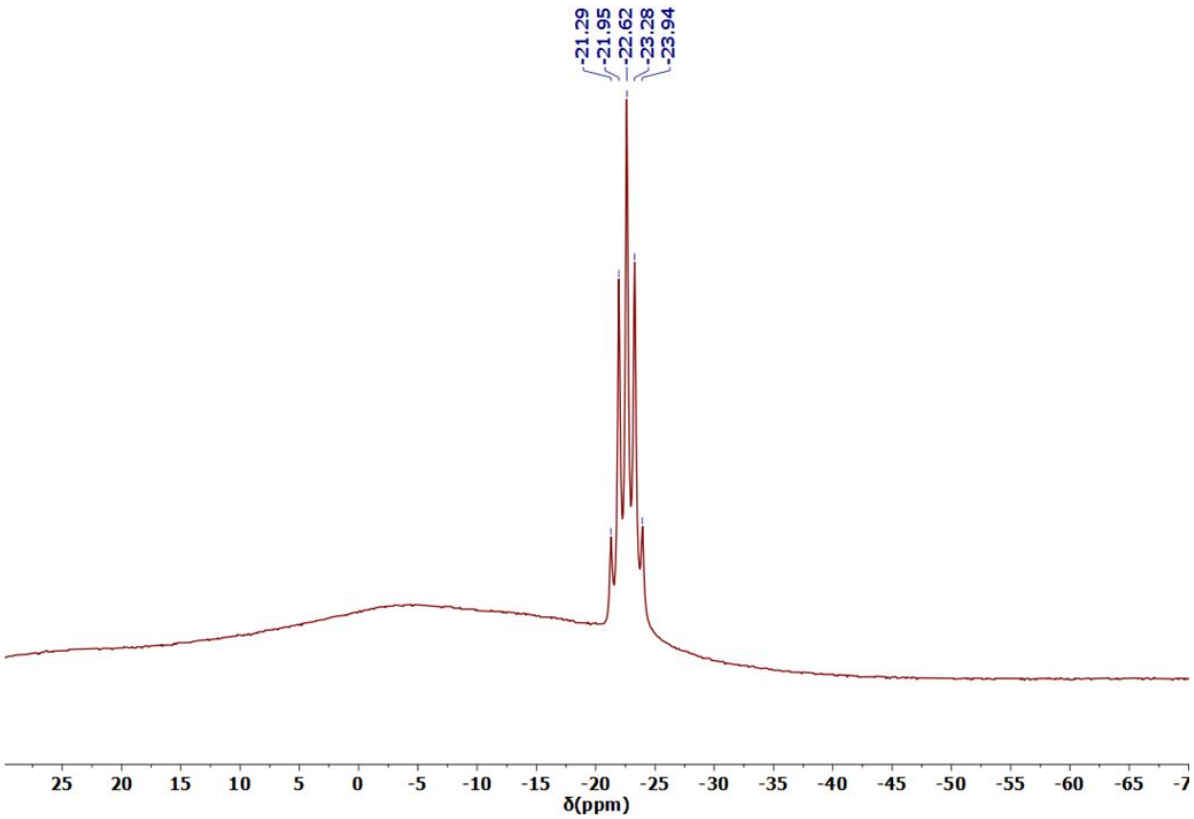

Figure S15. ^{11}B NMR spectrum of $\mathbf{1}_{\mathbf{Lu}}$ in benzene-D_6.

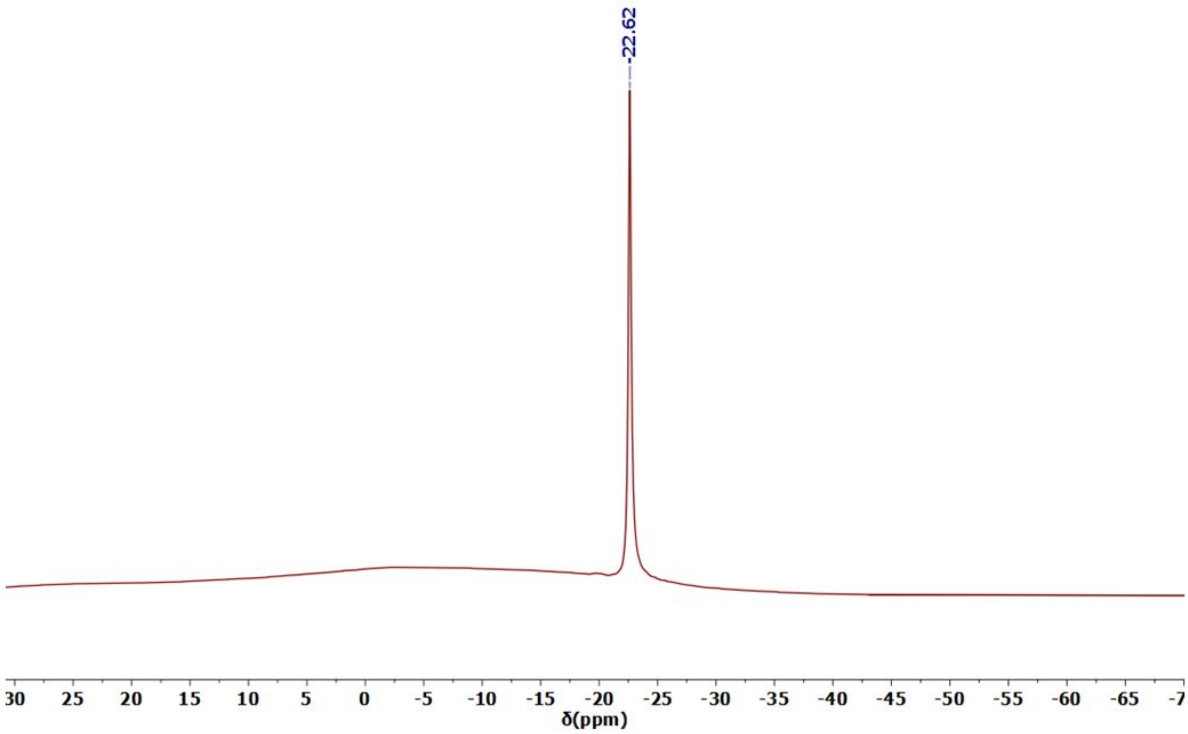

Figure S16. $^{11}B\{^{1}H\}$ NMR spectrum of $\mathbf{1_{Lu}}$ in benzene-D_6.

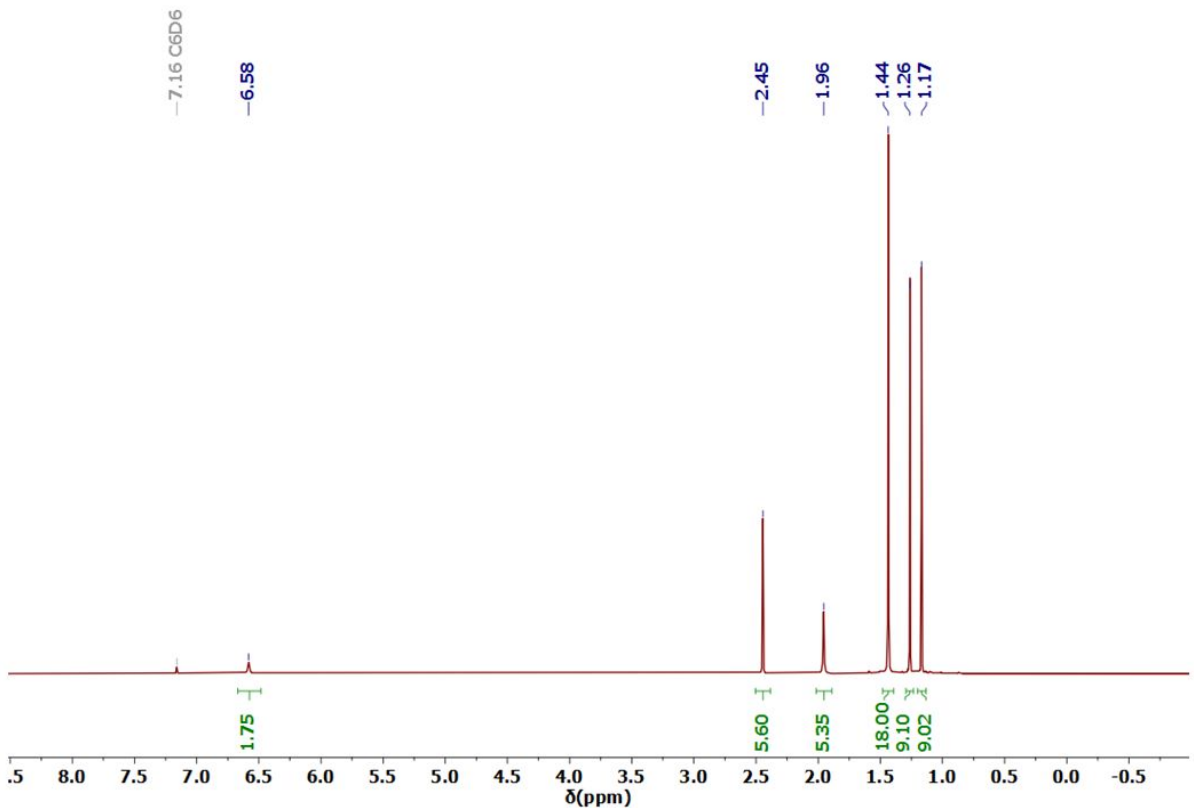

Figure S17. ^{1}H NMR spectrum of $\mathbf{2_Y}$ in benzene-D_6.

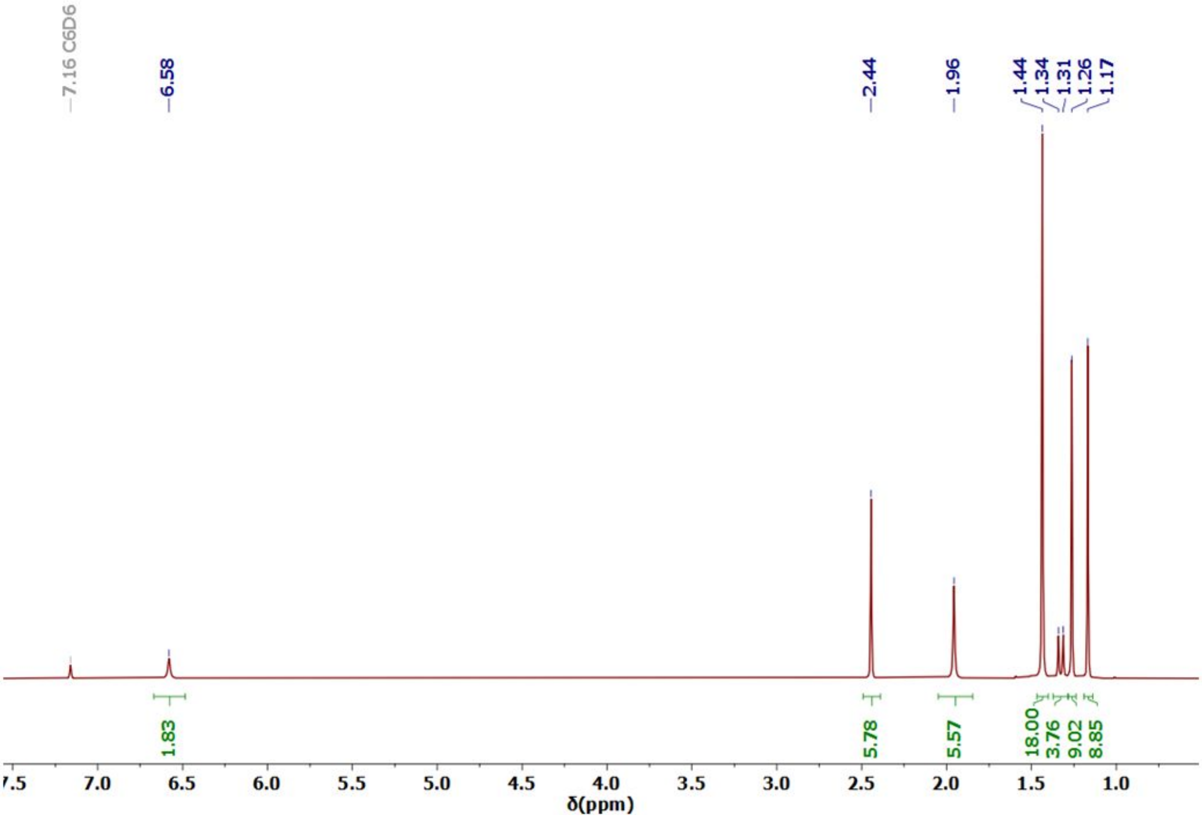

Figure S18. $^1H\{^{11}B\}$ NMR spectrum of $\mathbf{2_Y}$ in benzene-D_6.

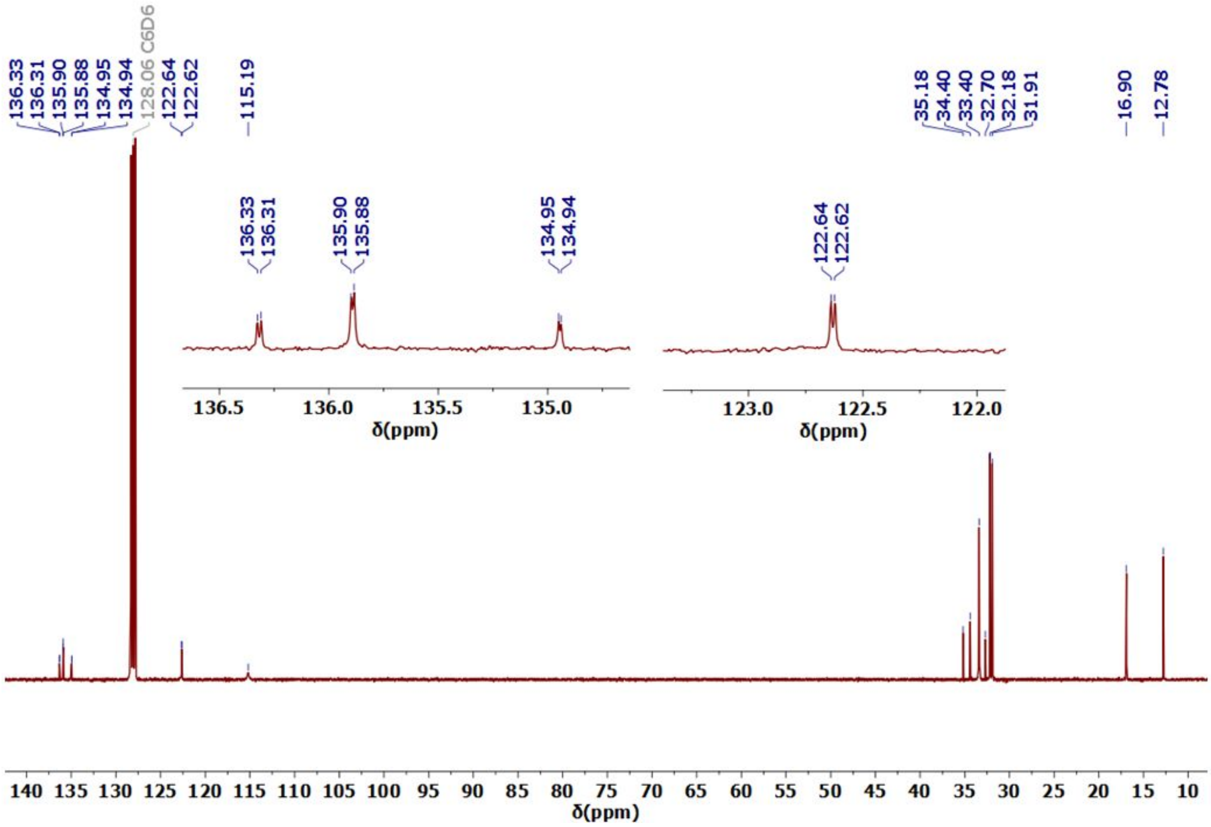

Figure S19. $^{13}C\{^{1}H\}$ NMR spectrum of $\mathbf{2_Y}$ in benzene-D_6. Inset: expansion of peaks corresponding to Cp ring carbons coupled to ^{89}Y.

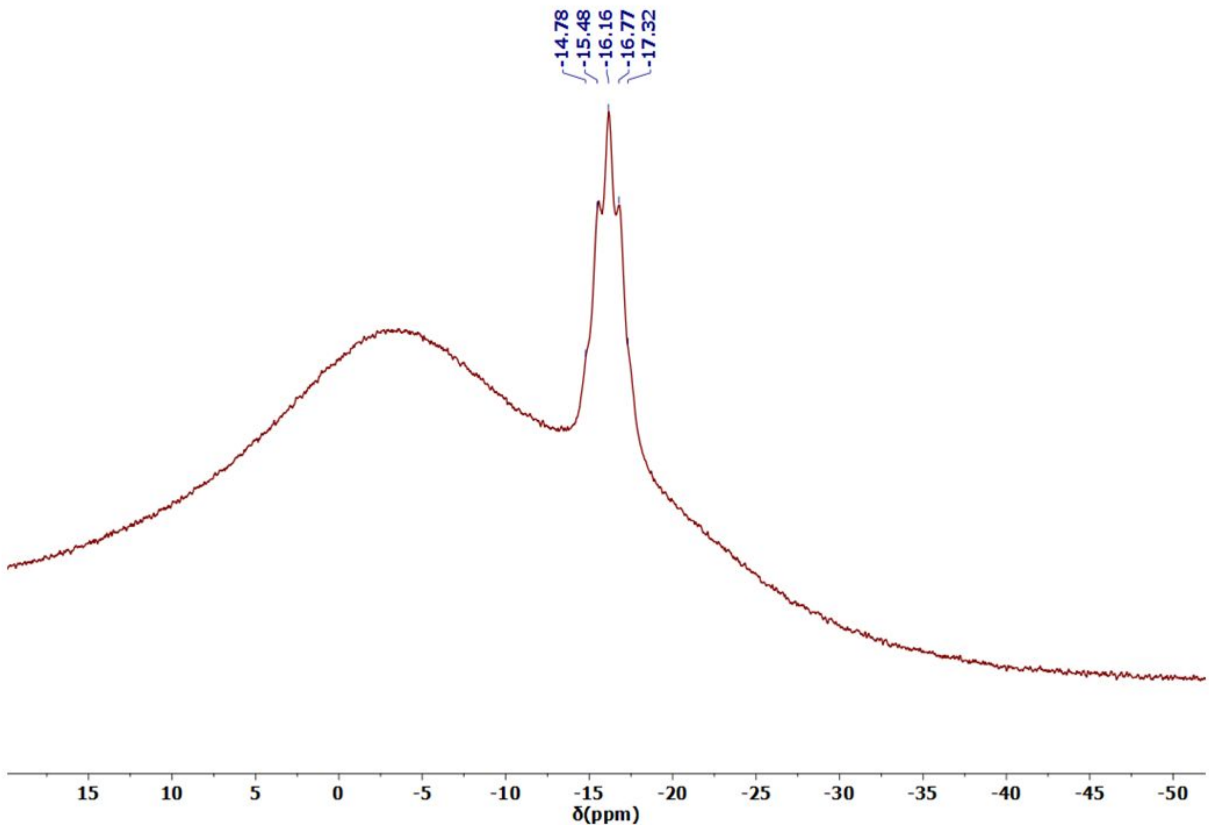

Figure S20. ^{11}B NMR spectrum of $\mathbf{2_Y}$ in benzene-D_6.

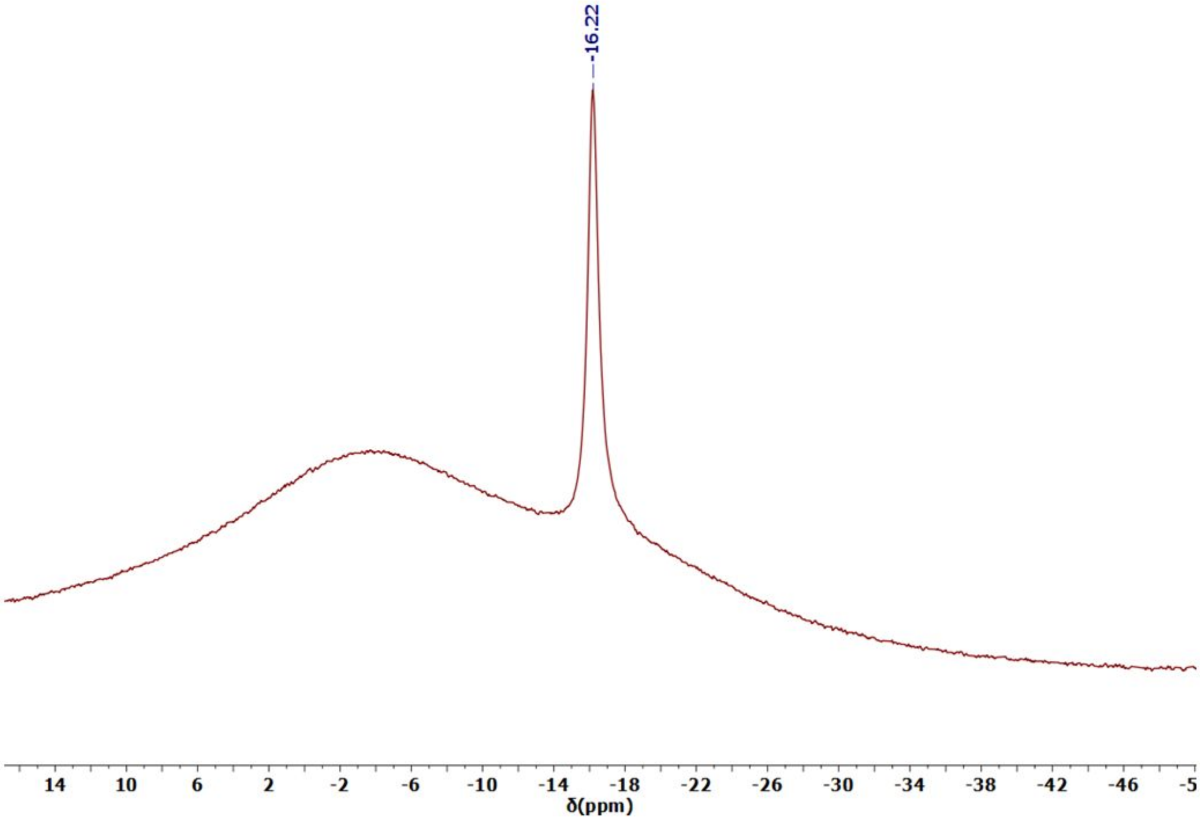

Figure S21. $^{11}B\{^1H\}$ NMR spectrum of $\mathbf{2_Y}$ in benzene-D_6.

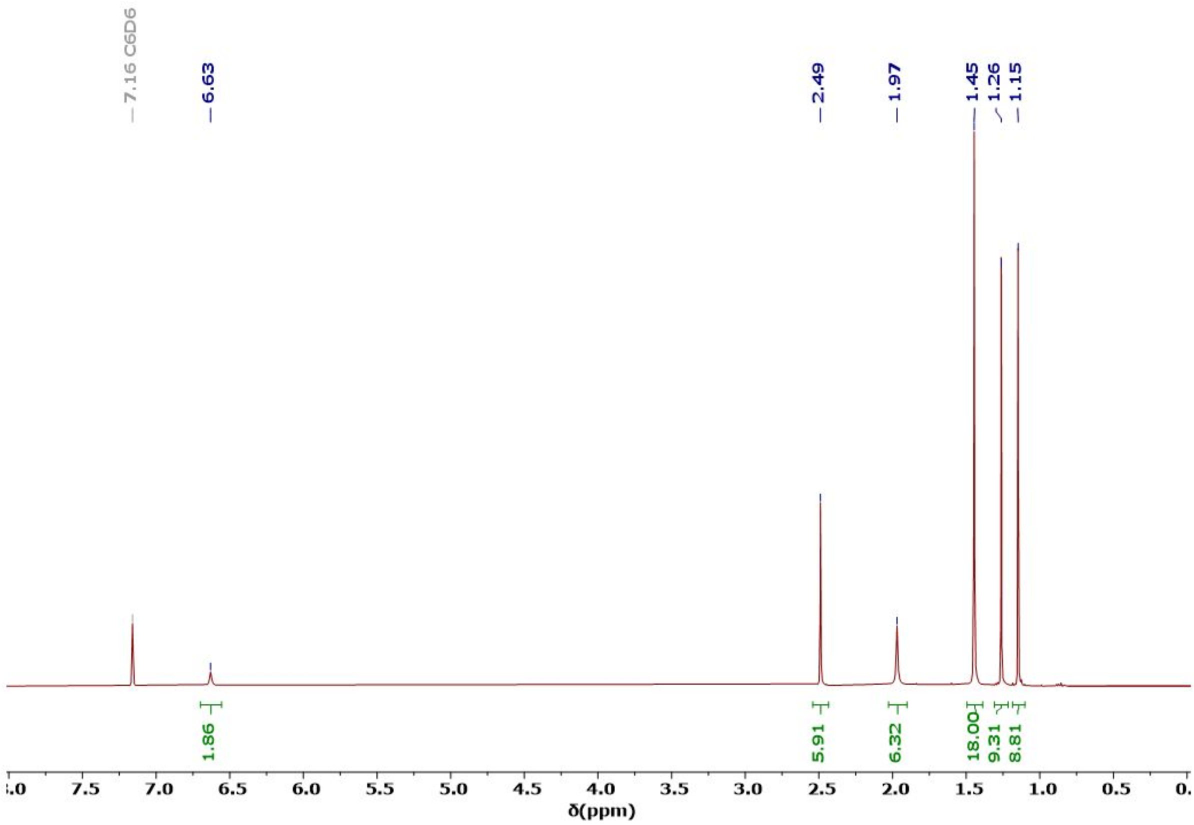

Figure S22. ^{1}H NMR spectrum of $\mathbf{2_{Lu}}$ in benzene-D$_6$.

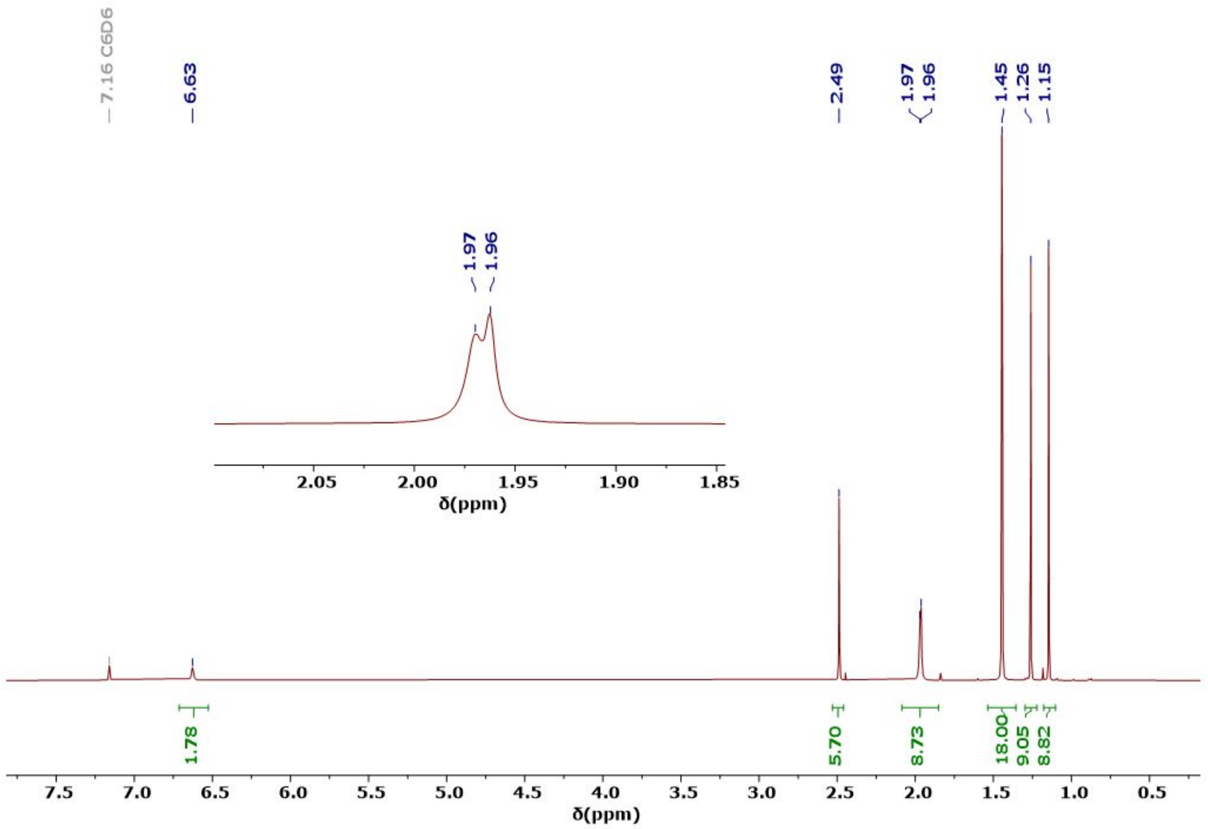

Figure S23. $^1H\{^{11}B\}$ NMR spectrum of $\mathbf{2_{Lu}}$ in benzene-D_6. Inset: expansion of overlapped borohydride and methyl proton signals.

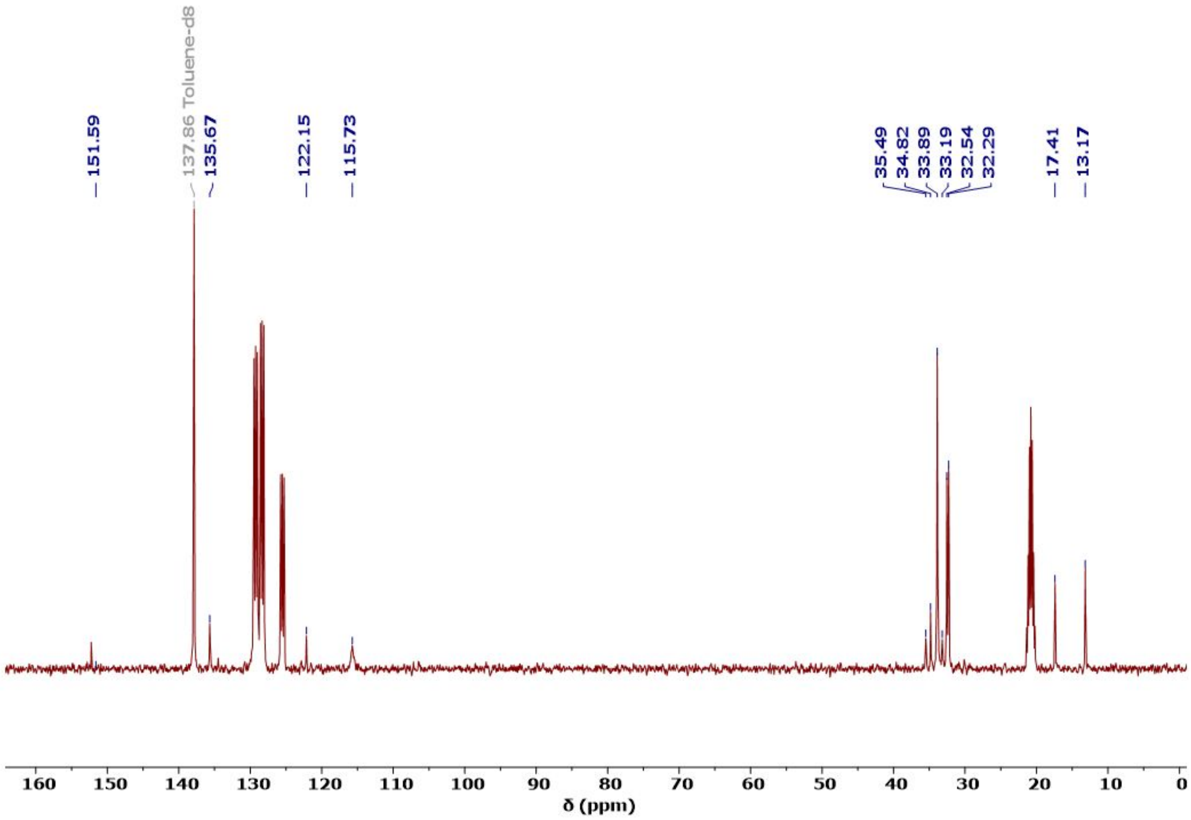

Figure S24. $^{13}C\{^{1}H\}$ NMR spectrum of $\mathbf{2_{Lu}}$ in toluene-D_8.

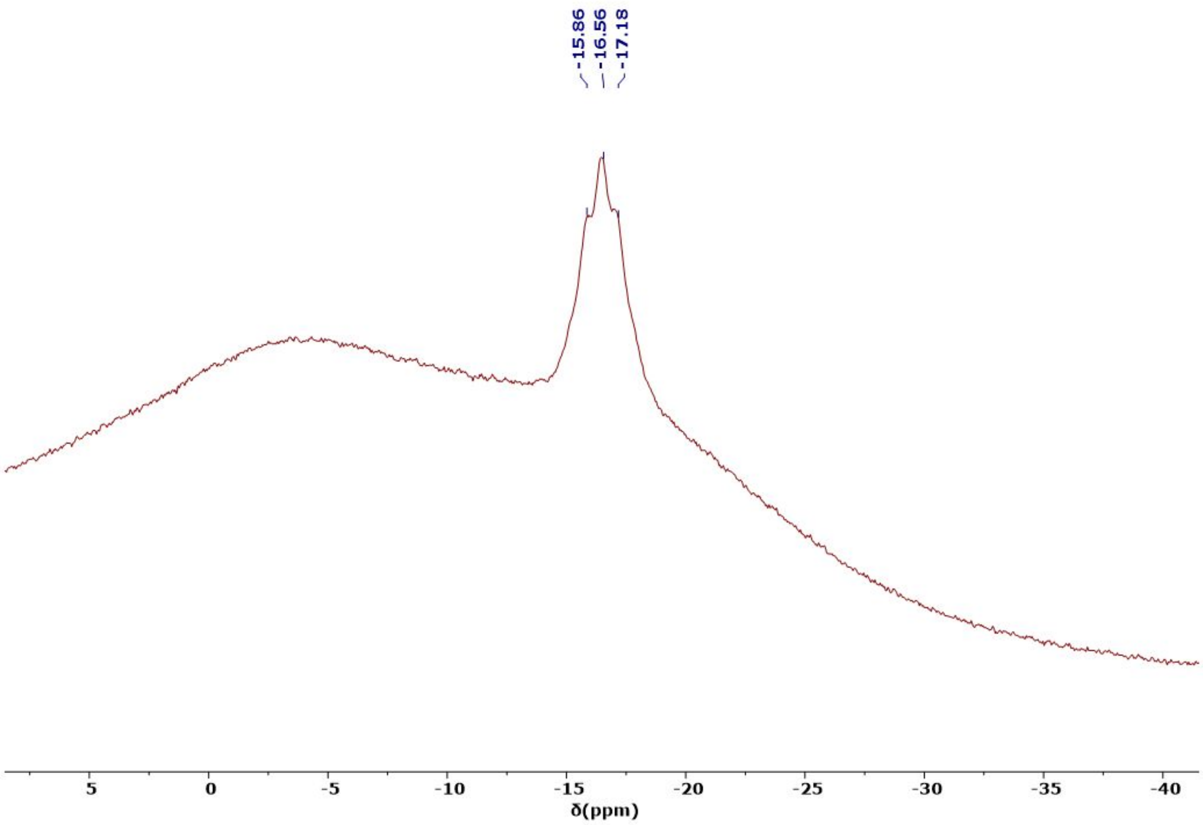

Figure S25. ^{11}B NMR spectrum of $\mathbf{2_{Lu}}$ in benzene-D_6.

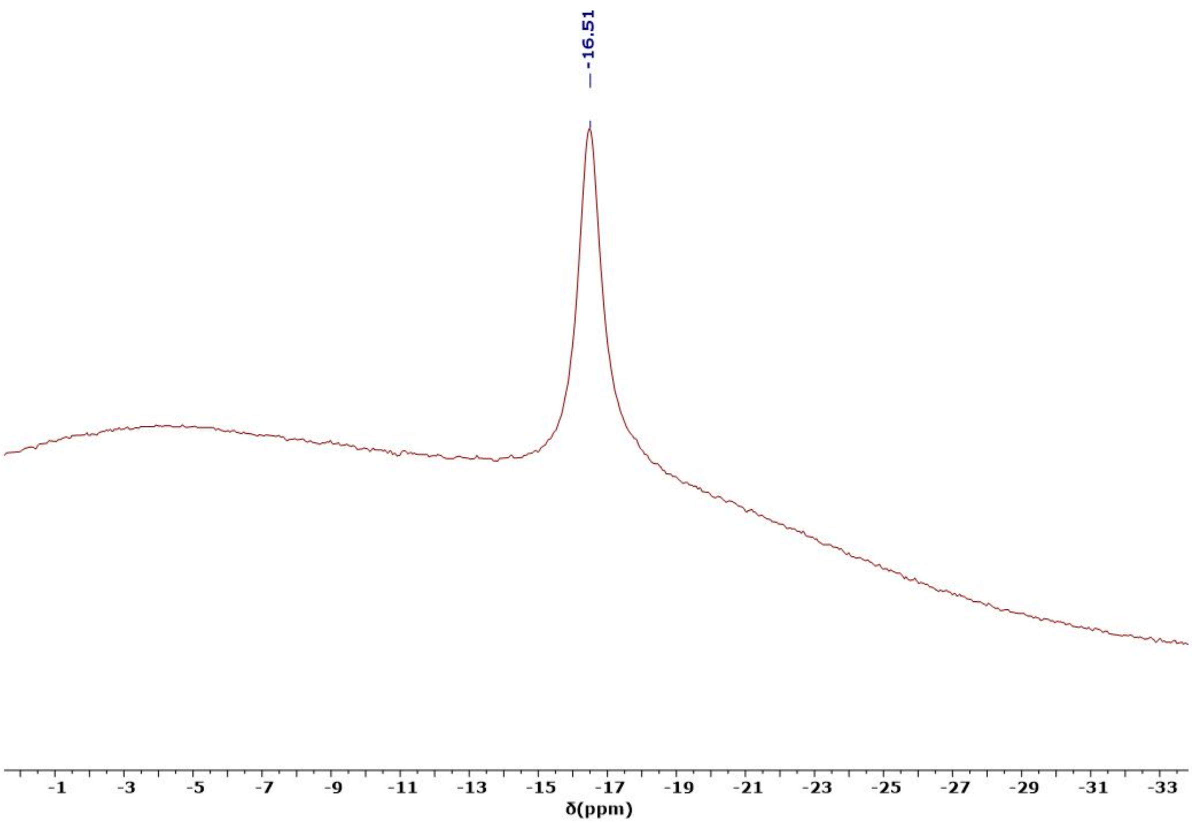

Figure S26. $^{11}B\{^{1}H\}$ NMR spectrum of $\mathbf{2_{Lu}}$ in benzene-D_6.

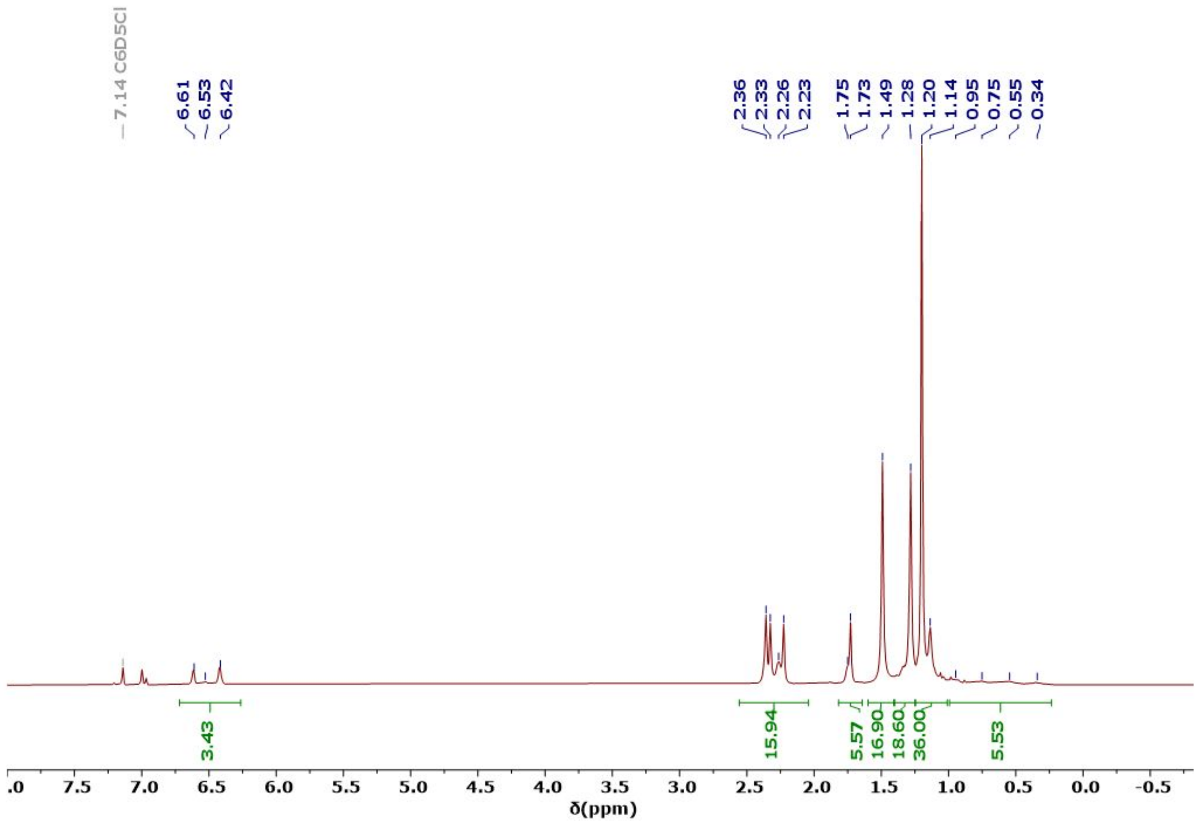

Figure S27. ^{1}H NMR spectrum of $\mathbf{3_Y}$ in chlorobenzene-D_5 at 30 °C.

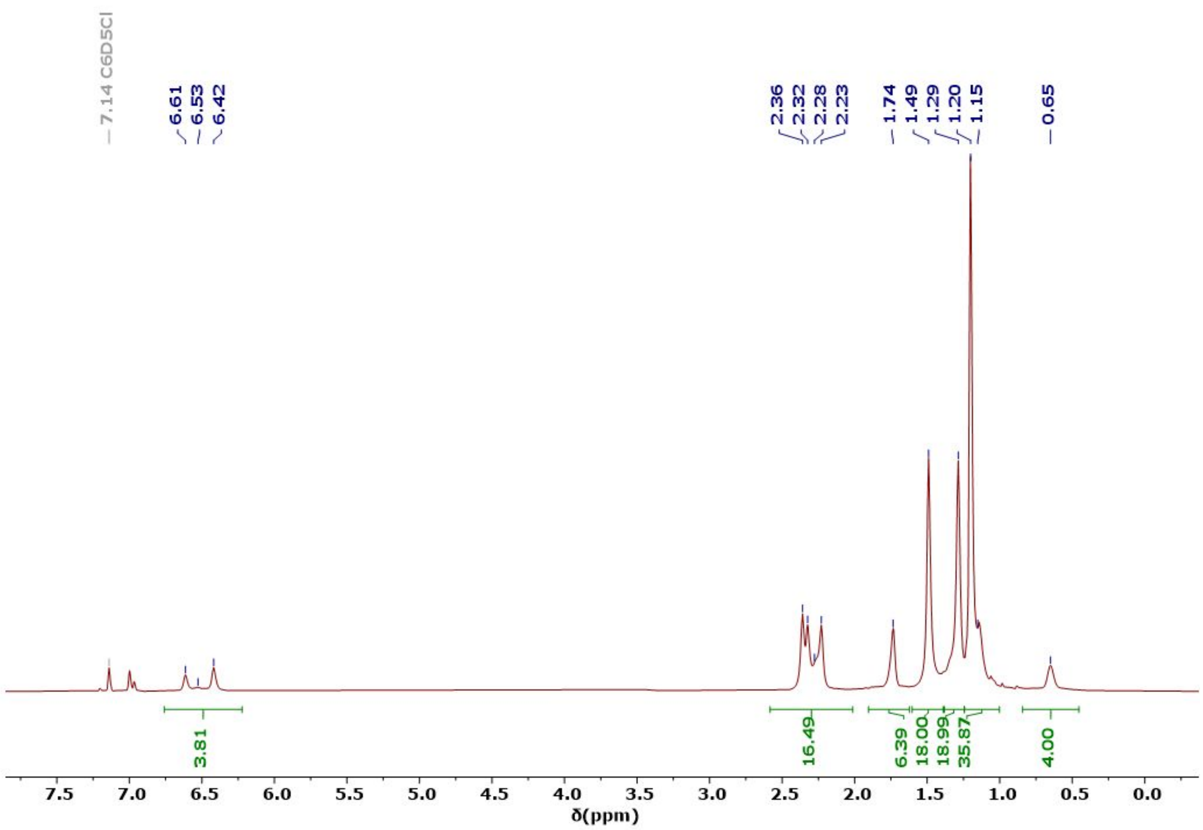

Figure S28. $^{1}H\{^{11}B\}$ NMR spectrum of $\mathbf{3_Y}$ in chlorobenzene-D_5 at 30 °C.

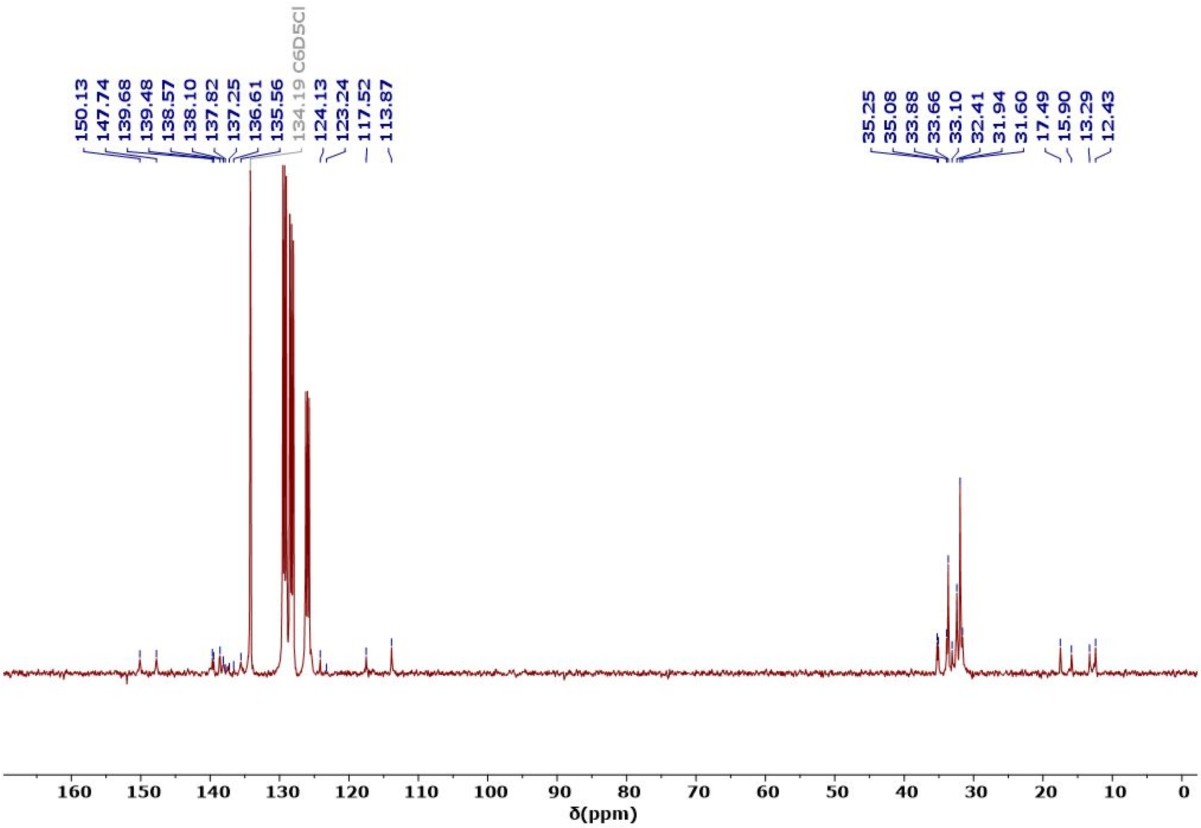

Figure S29. $^{13}C\{^{1}H\}$ NMR spectrum of $\mathbf{3_Y}$ in chlorobenzene-D_5 at 30 °C.

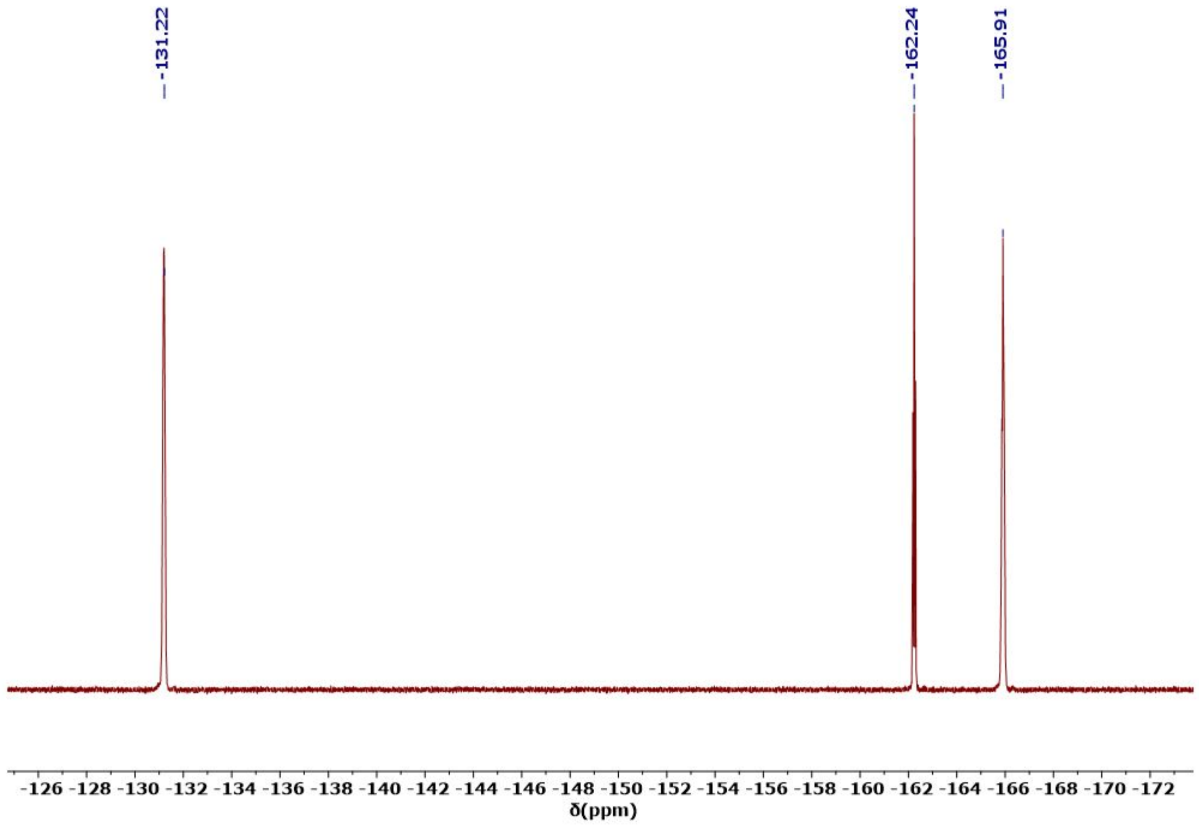

Figure S30. ^{19}F NMR spectrum of $\mathbf{3_Y}$ in chlorobenzene-D_5 at 30 °C.

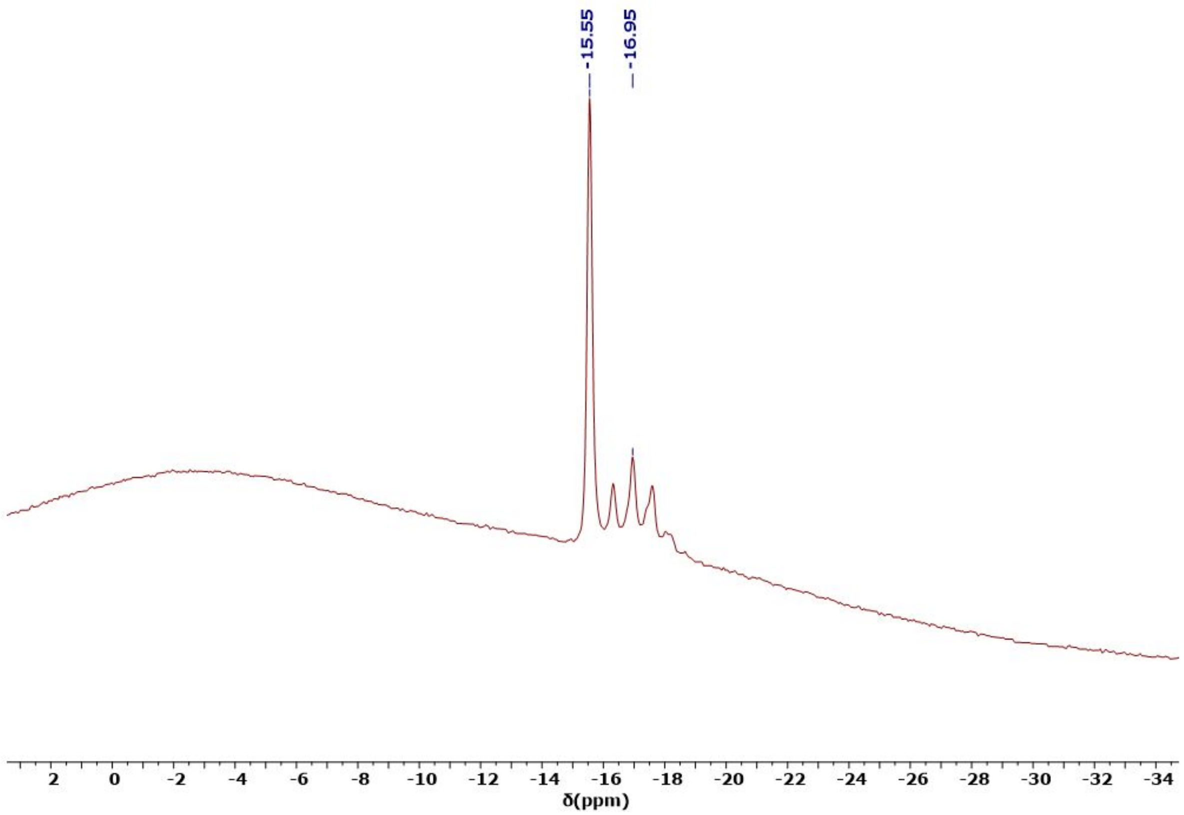

Figure S31. ^{11}B NMR spectrum of $\mathbf{3_Y}$ in chlorobenzene-D_5 at 30 °C.

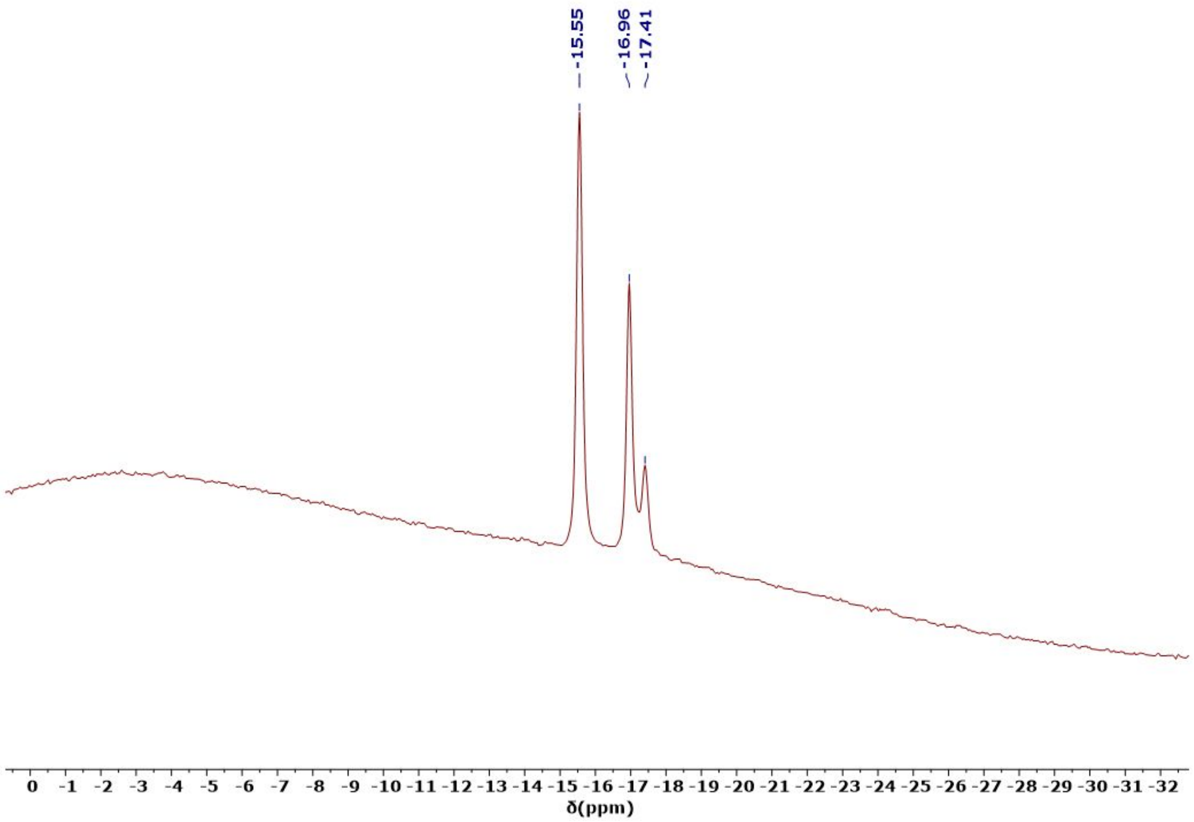

Figure S32. $^{11}B\{^{1}H\}$ NMR spectrum of $\mathbf{3_Y}$ in chlorobenzene-D_5 at 30 °C.

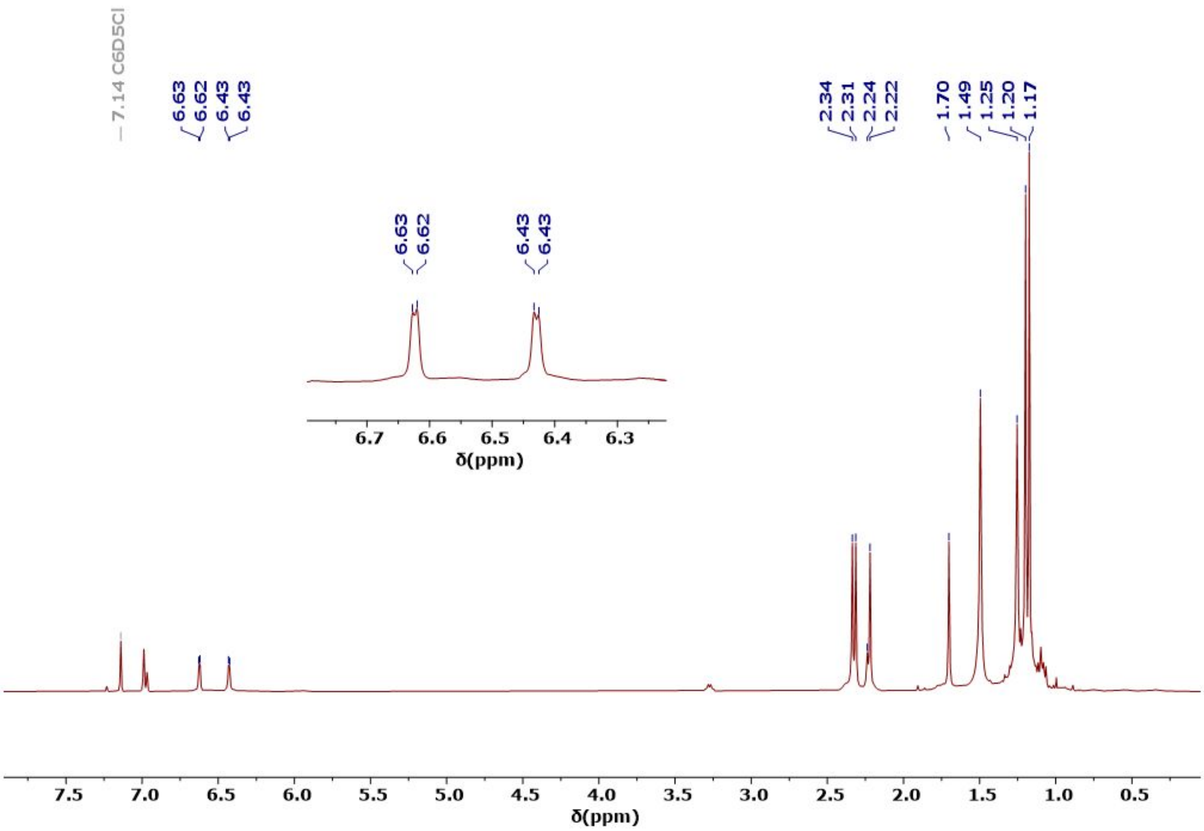

Figure S33. ^{1}H NMR spectrum of $\mathbf{3_Y}$ in chlorobenzene-D_5 at –30 °C. Inset: expansion of the signals corresponding to the Cp^{ttt} ring protons, revealing the $^4J_{HH}$ coupling.

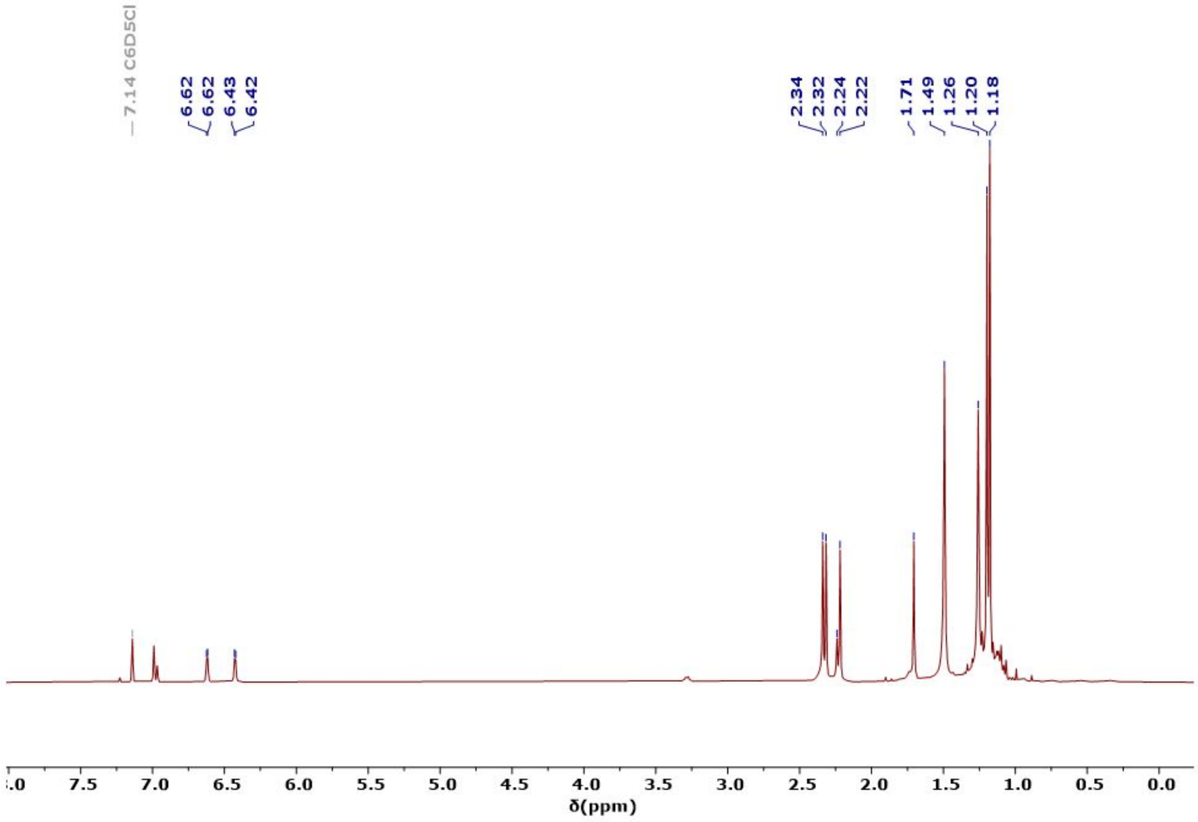

Figure S34. ^{1}H NMR spectrum of $\mathbf{3_Y}$ in chlorobenzene-D_5 at –20 °C.

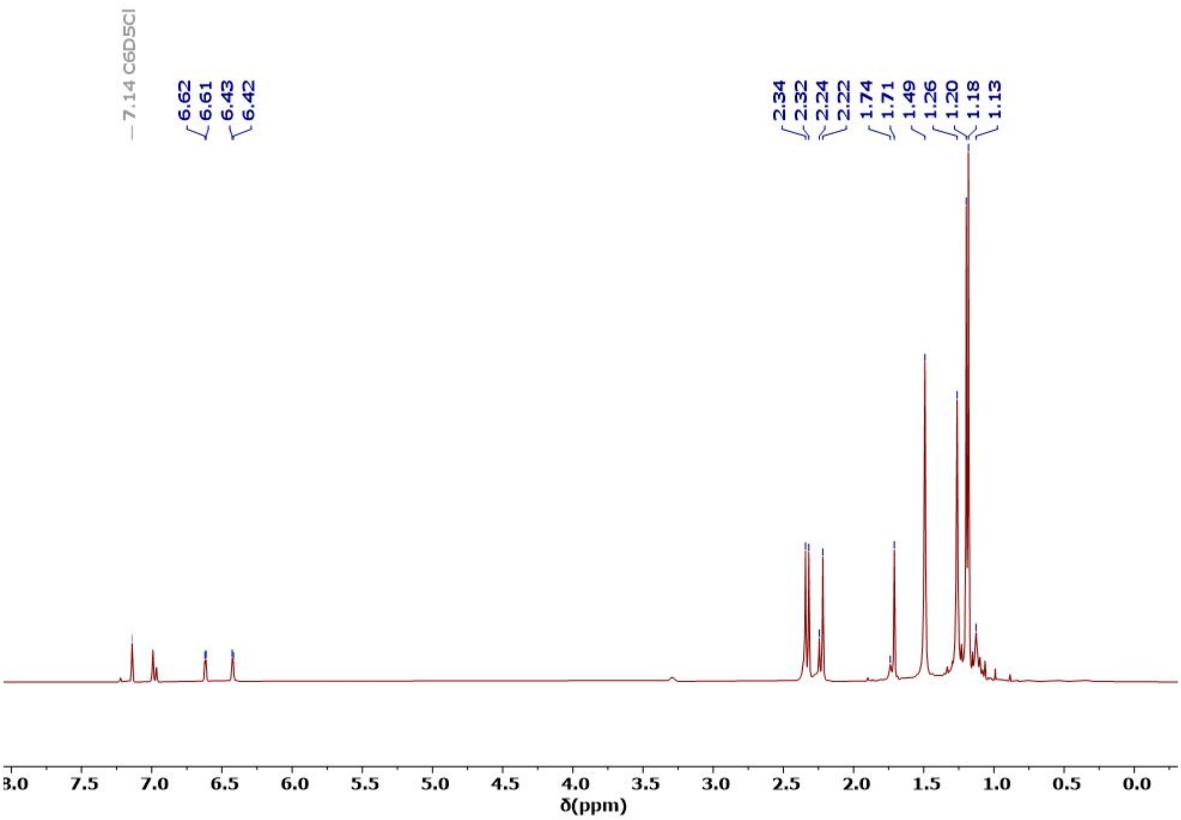

Figure S35. ^{1}H NMR spectrum of $\mathbf{3_Y}$ in chlorobenzene-D_5 at –10 °C.

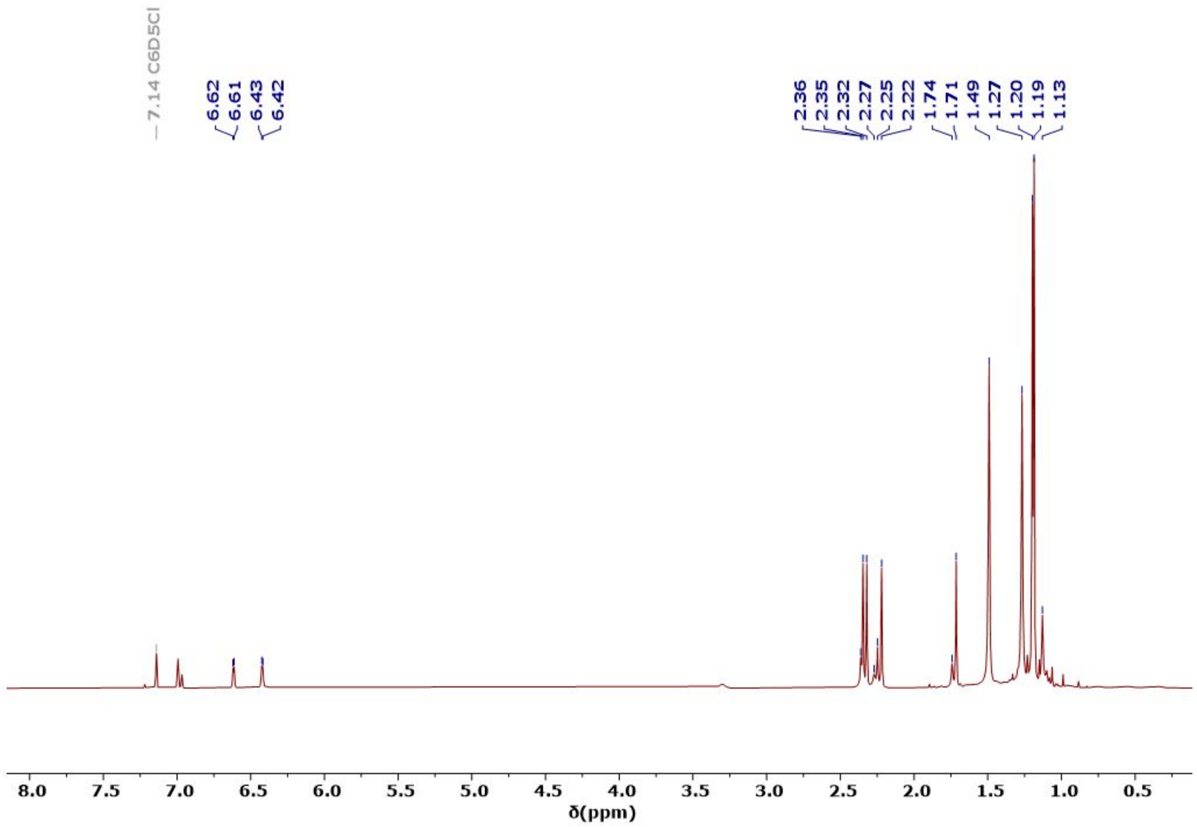

Figure S36. ^{1}H NMR spectrum of $\mathbf{3_Y}$ in chlorobenzene-D_5 at 0 °C.

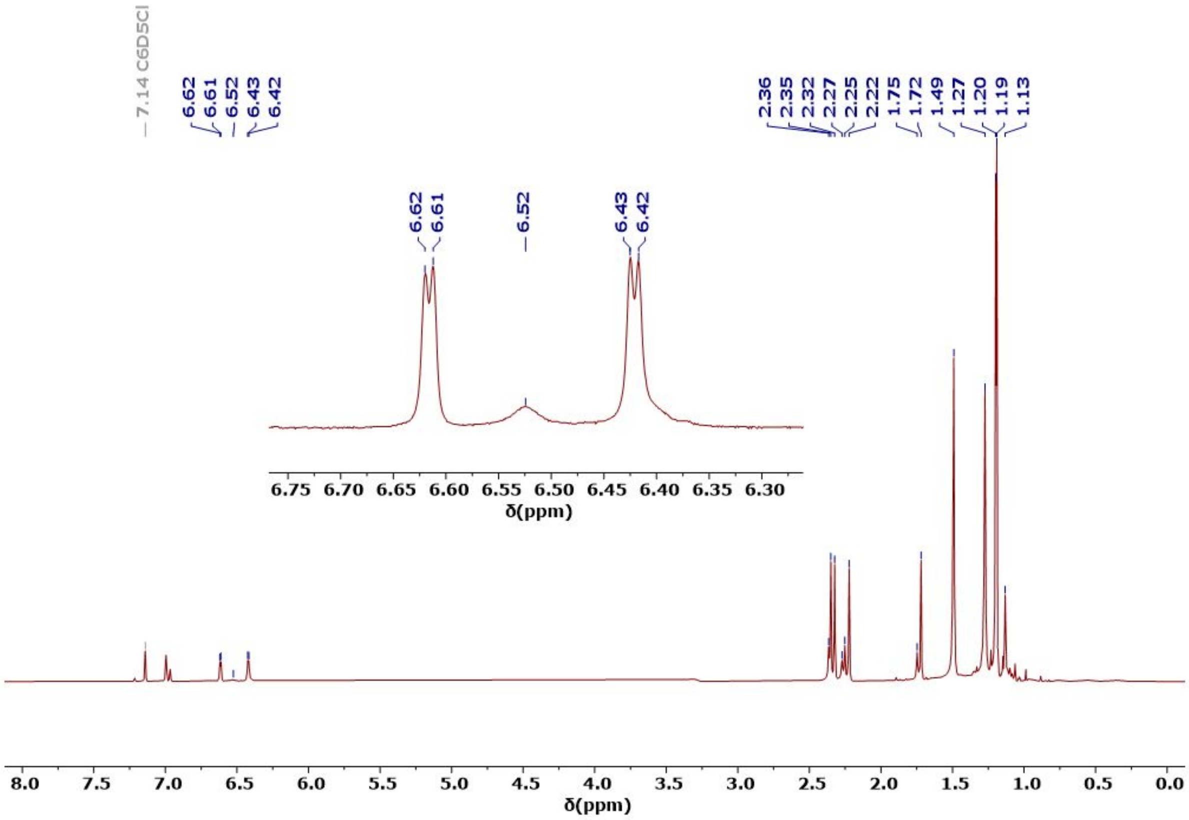

Figure S37. ^{1}H NMR spectrum of $\mathbf{3_Y}$ in chlorobenzene-D_5 at 10 °C. Inset: expansion of the signals corresponding to the Cp^{ttt} ring protons, showing the emergence of a broad signal at 6.52 ppm.

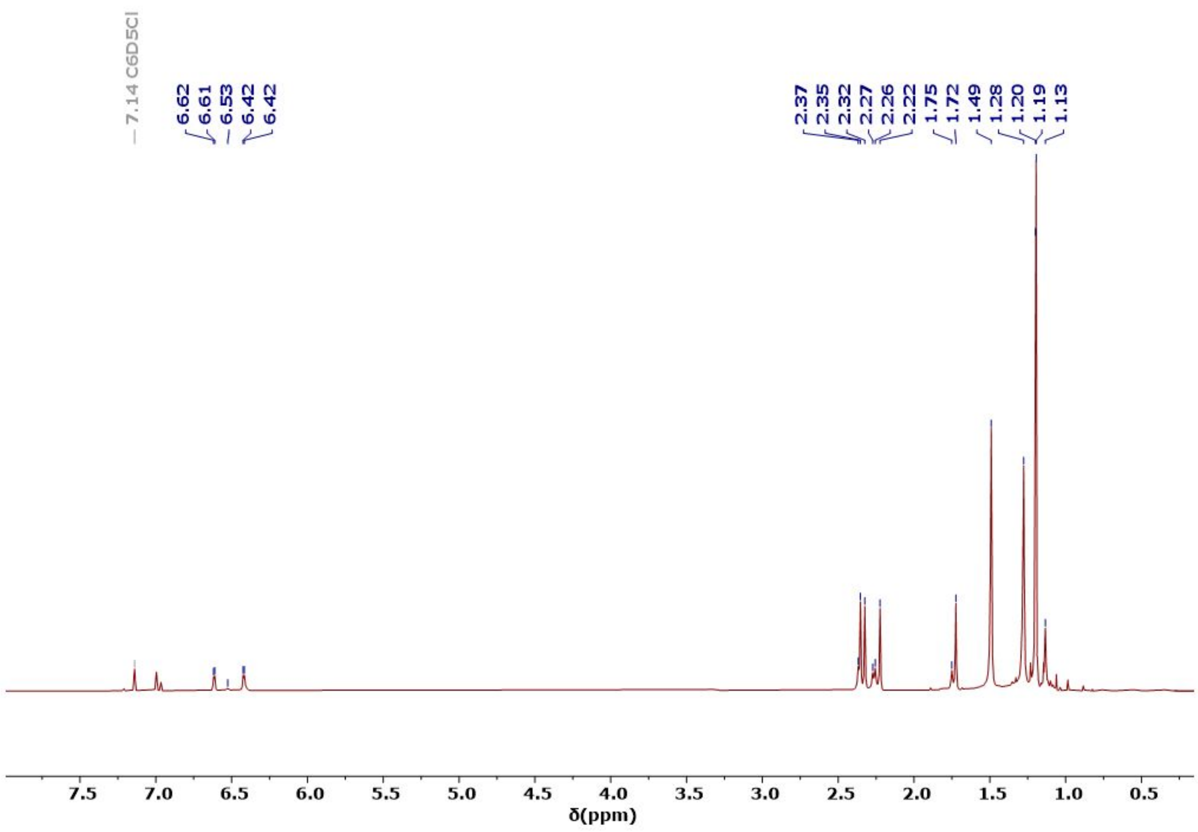

Figure S38. ^{1}H NMR spectrum of $\mathbf{3_{Y}}$ in chlorobenzene-D_5 at 20 °C.

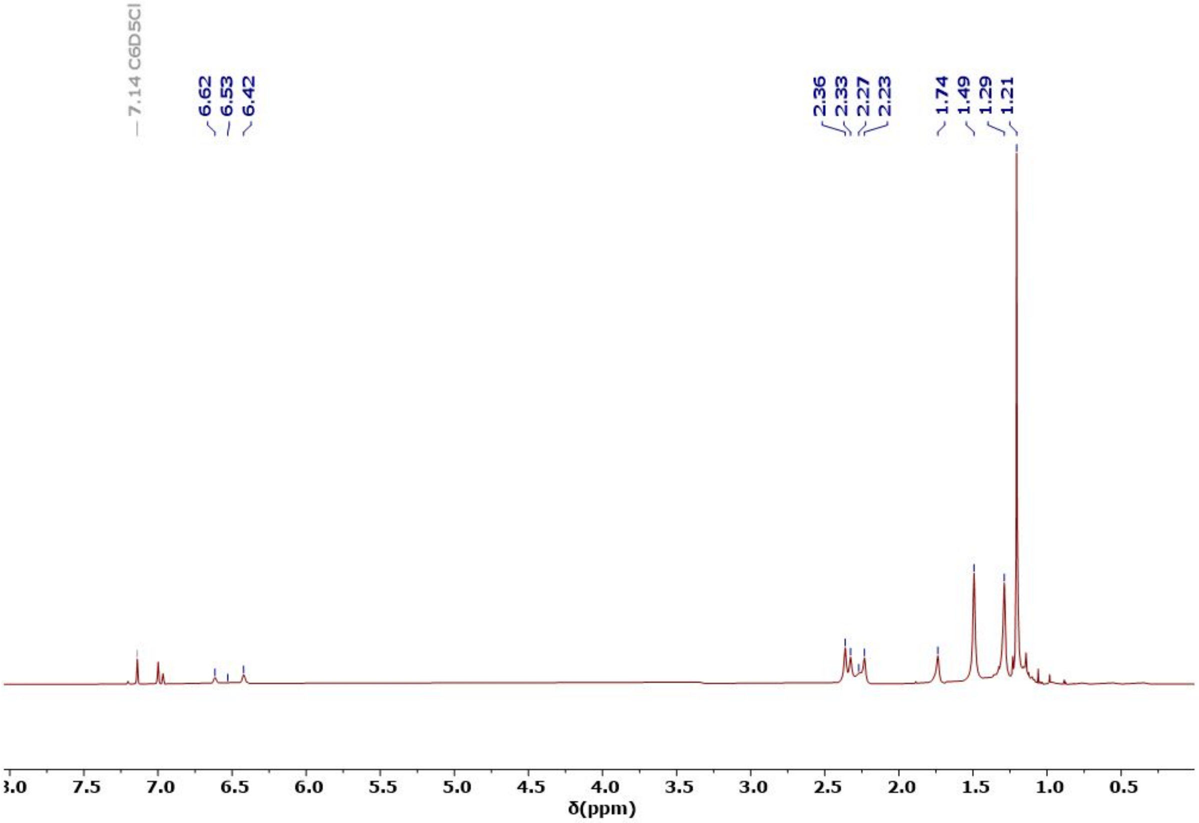

Figure S39. ^{1}H NMR spectrum of $\mathbf{3_Y}$ in chlorobenzene-D_5 at 40 °C.

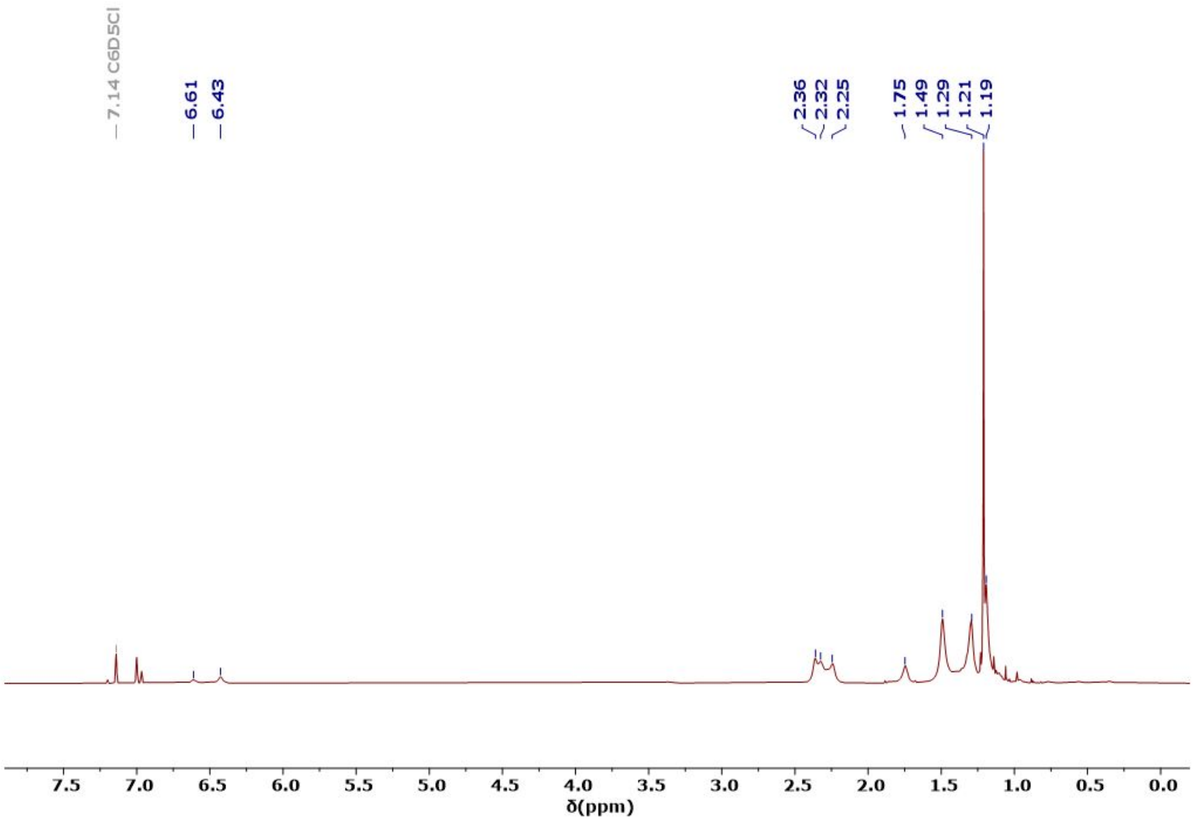

Figure S40. ^{1}H NMR spectrum of $\mathbf{3_Y}$ in chlorobenzene-D_5 at 50 °C.

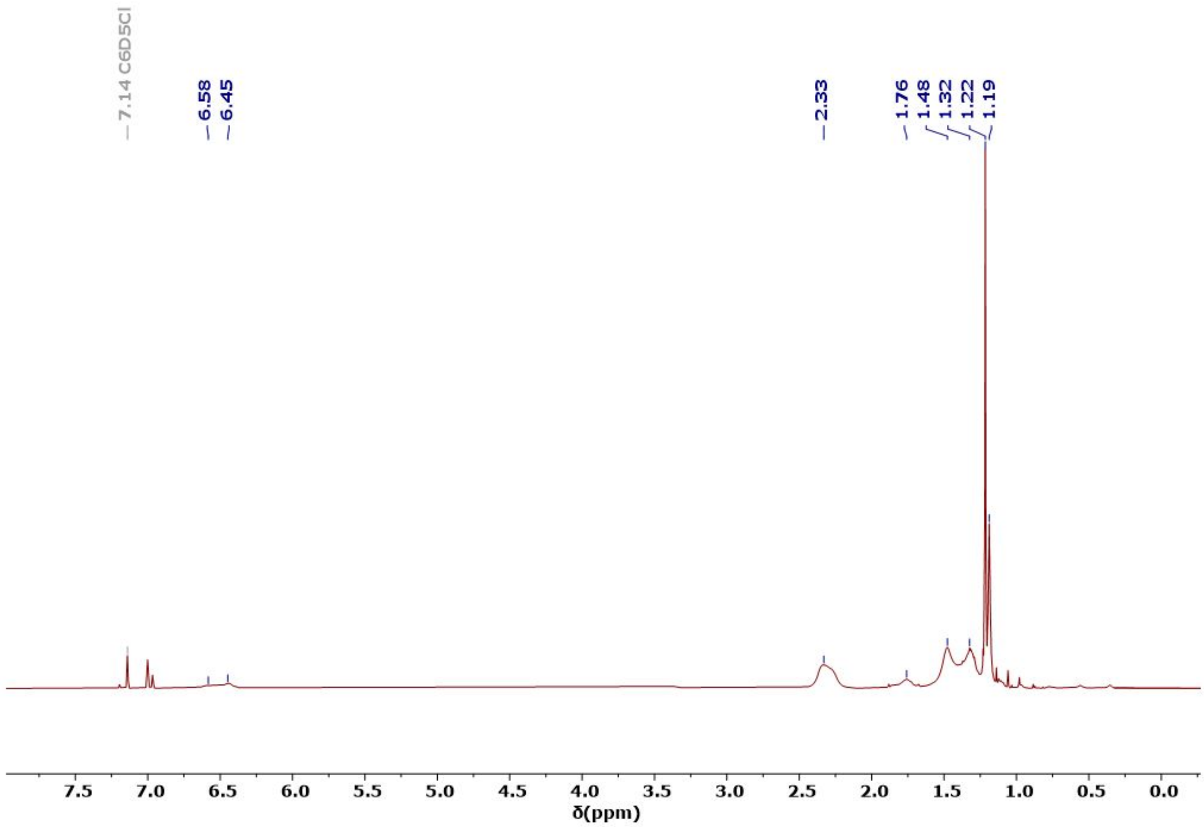

Figure S41. ^{1}H NMR spectrum of $\mathbf{3_Y}$ in chlorobenzene-D_5 at 60 °C.

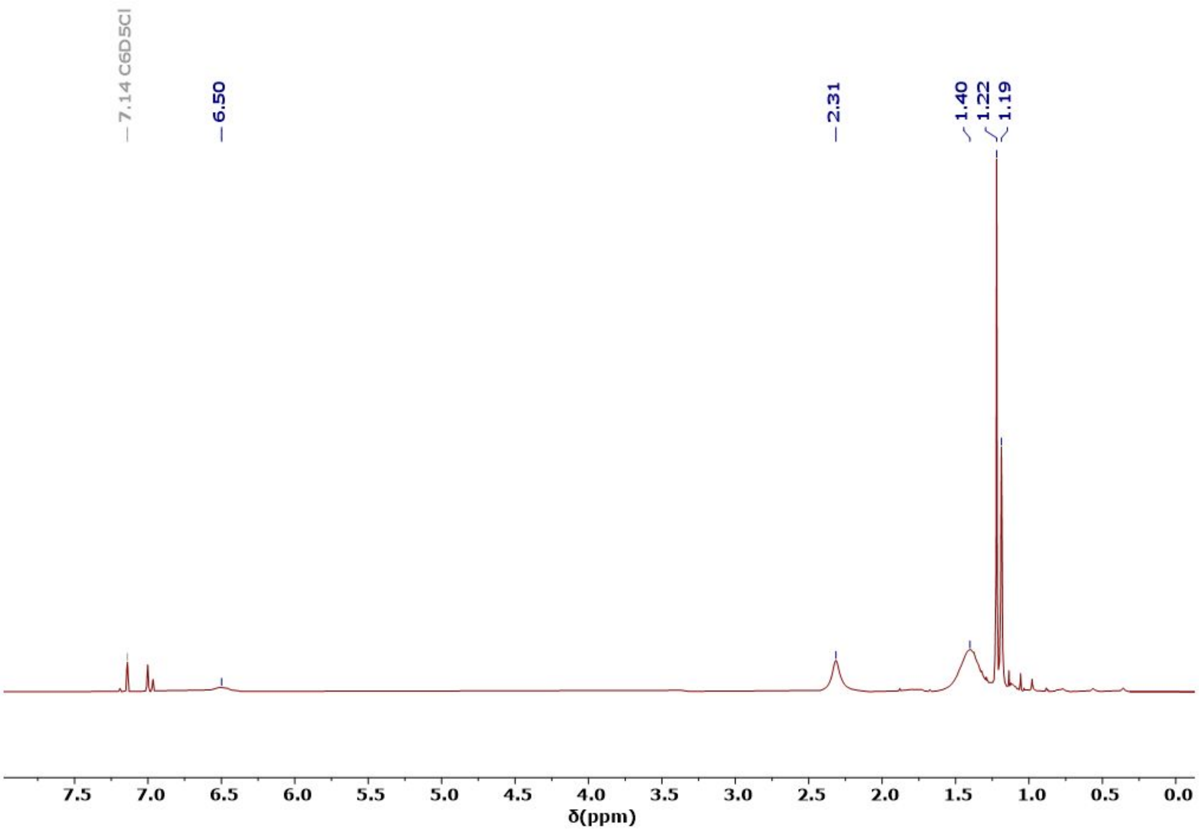

Figure S42. ^{1}H NMR spectrum of $\mathbf{3_Y}$ in chlorobenzene-D_5 at 70 °C.

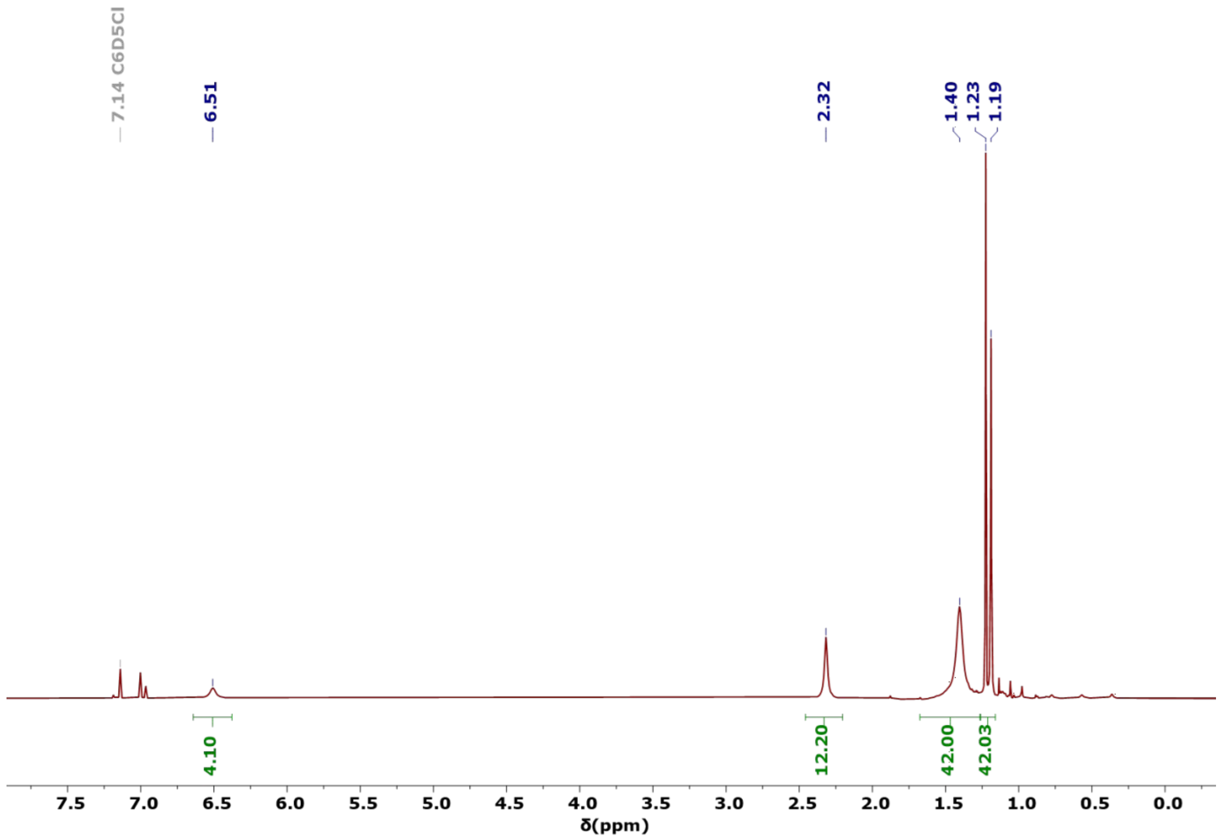

Figure S43. ^{1}H NMR spectrum of $\mathbf{3_{\gamma}}$ in chlorobenzene-D_5 at 80 °C.

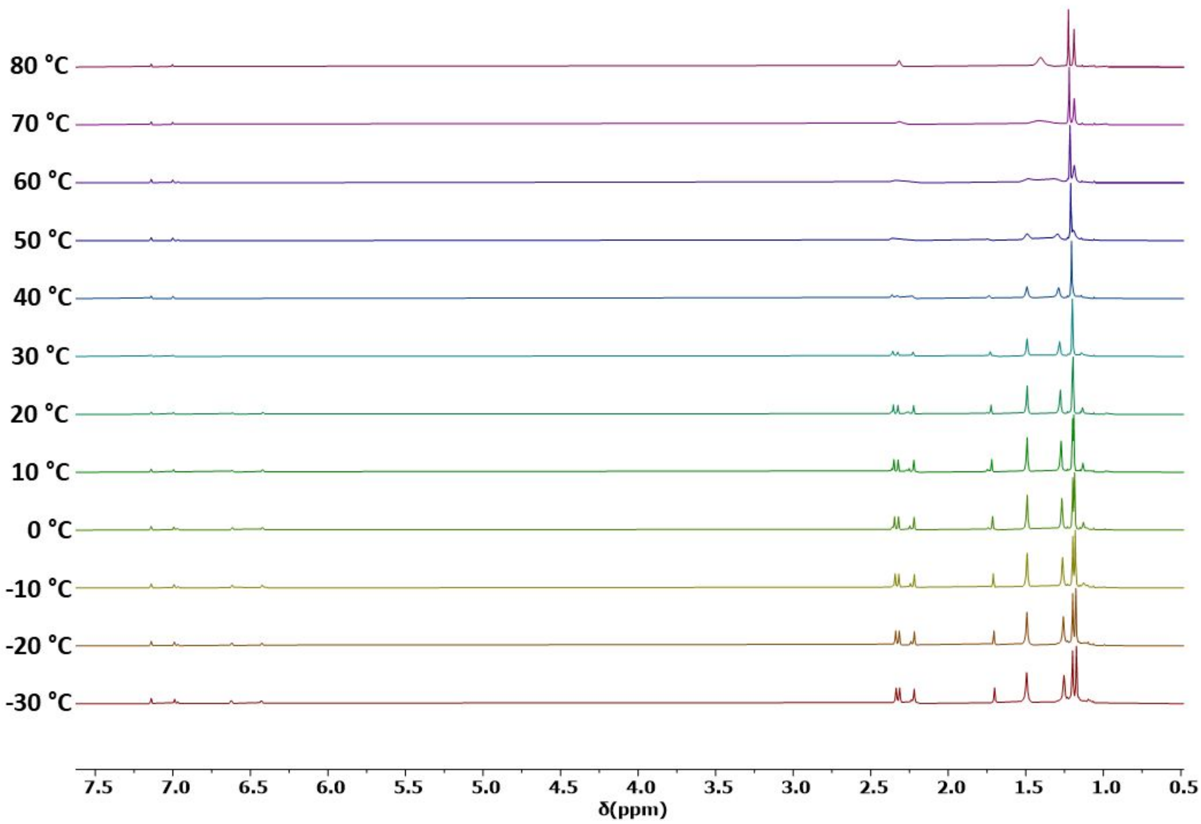

Figure S44. Variable-temperature ^{1}H NMR spectra of **3**$_{\mathbf{Y}}$ from –30 °C to 80 °C in chlorobenzene-D_5.

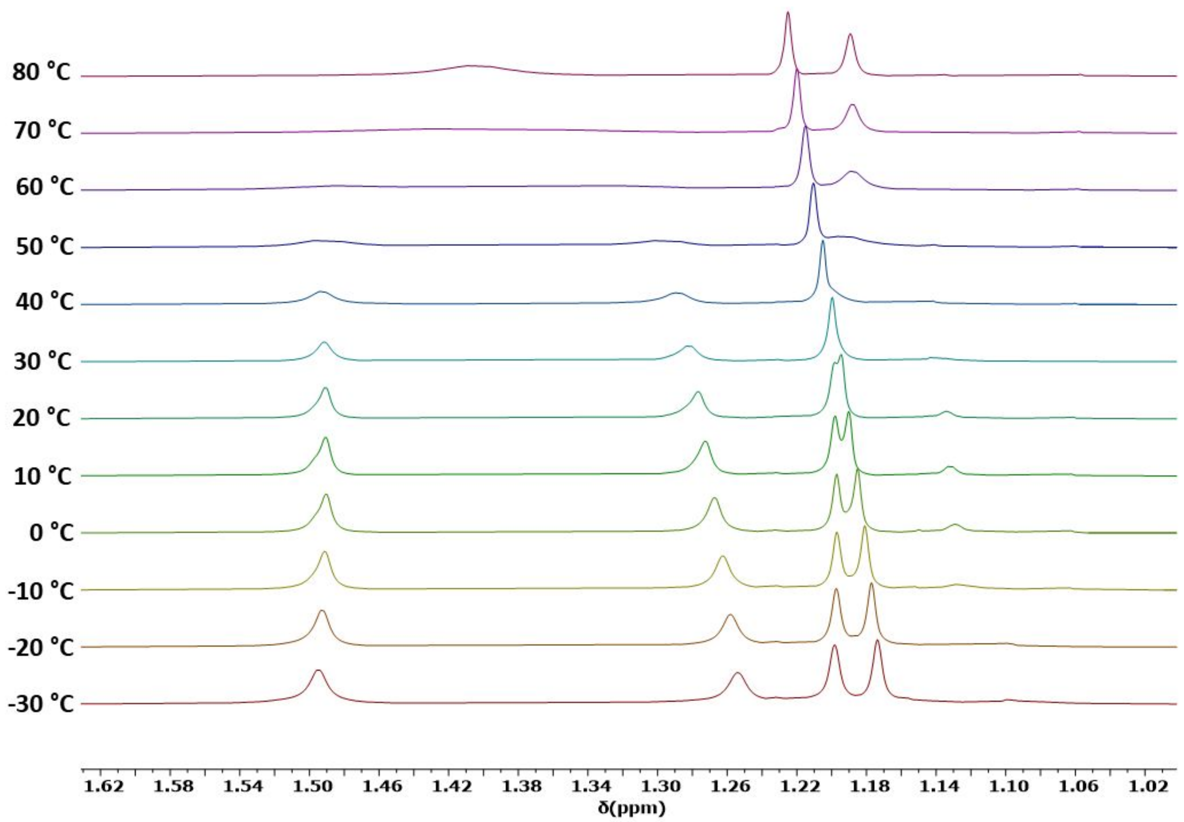

Figure S45. Variable-temperature ^{1}H NMR spectra of $\mathbf{3_Y}$ in the 1.0-1.6 ppm range from –30 °C to 80 °C in chlorobenzene-D_5.

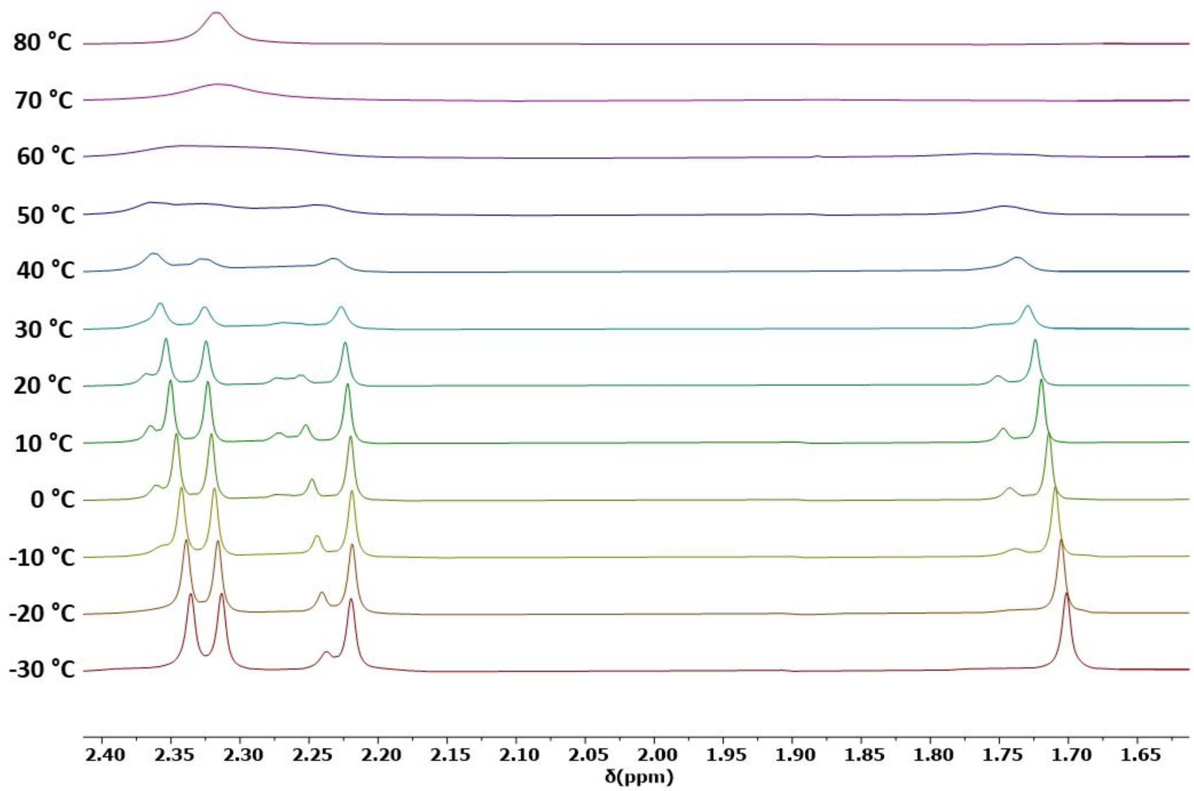

Figure S46. Variable-temperature ^{1}H NMR spectra of $\mathbf{3_Y}$ in the 1.6-2.4 ppm range from –30 °C to 80 °C in chlorobenzene-D_5.

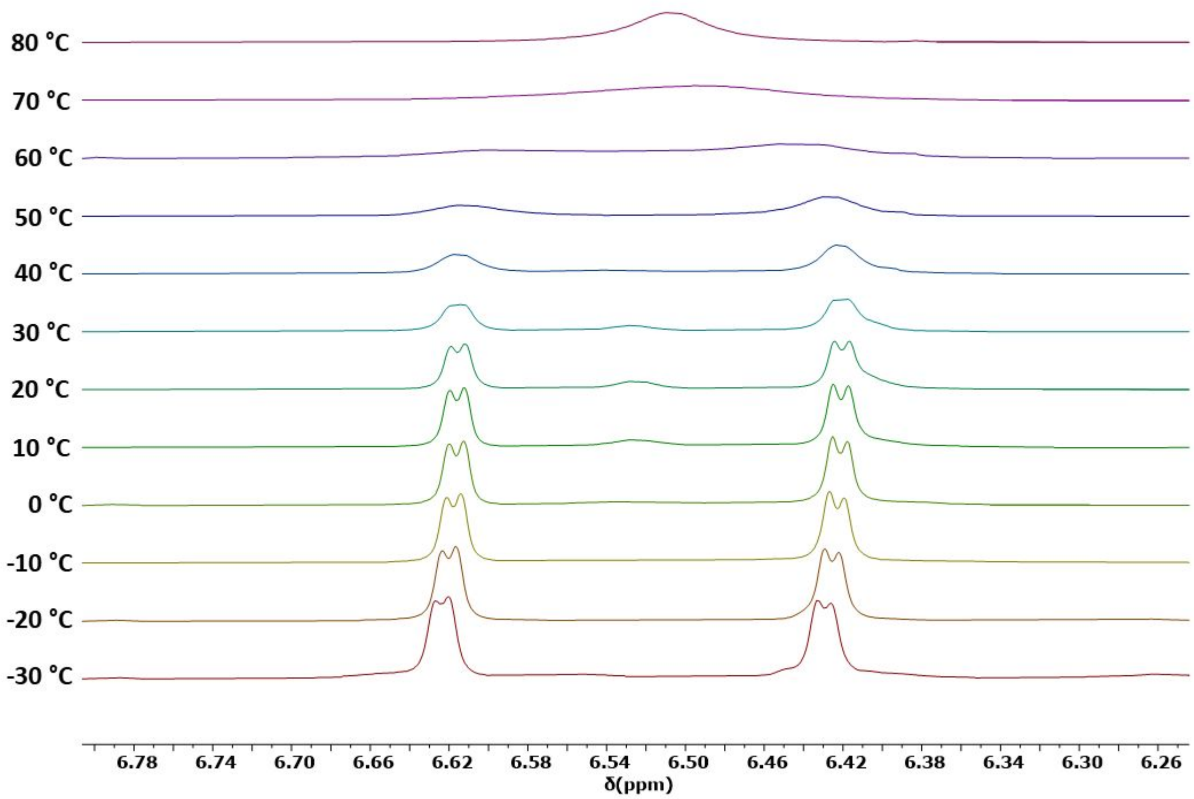

Figure S47. Variable-temperature ^{1}H NMR spectra of $\mathbf{3_Y}$ in the 6.2-6.8 ppm range from –30 °C to 80 °C in chlorobenzene-D_5.

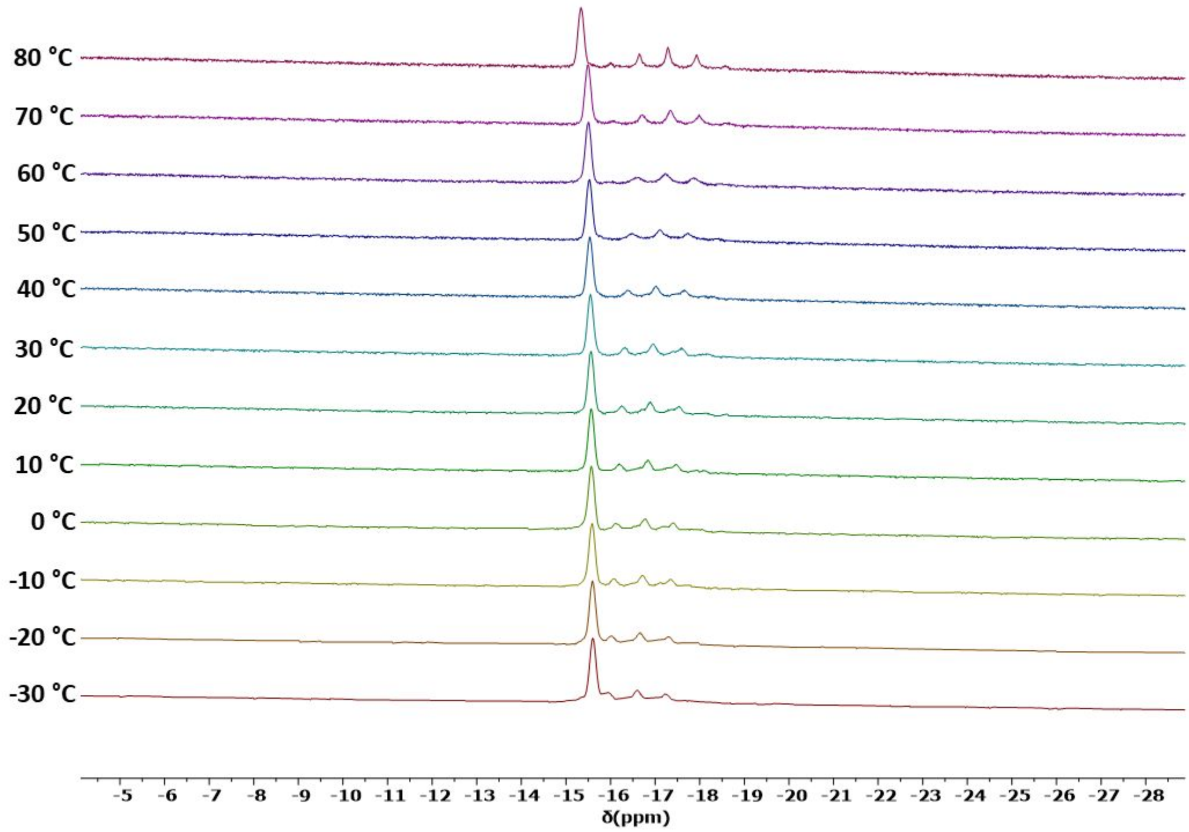

Figure S48. Variable-temperature ^{11}B NMR spectra of $\mathbf{3_Y}$ from –30 °C to 80 °C in chlorobenzene-D_5.

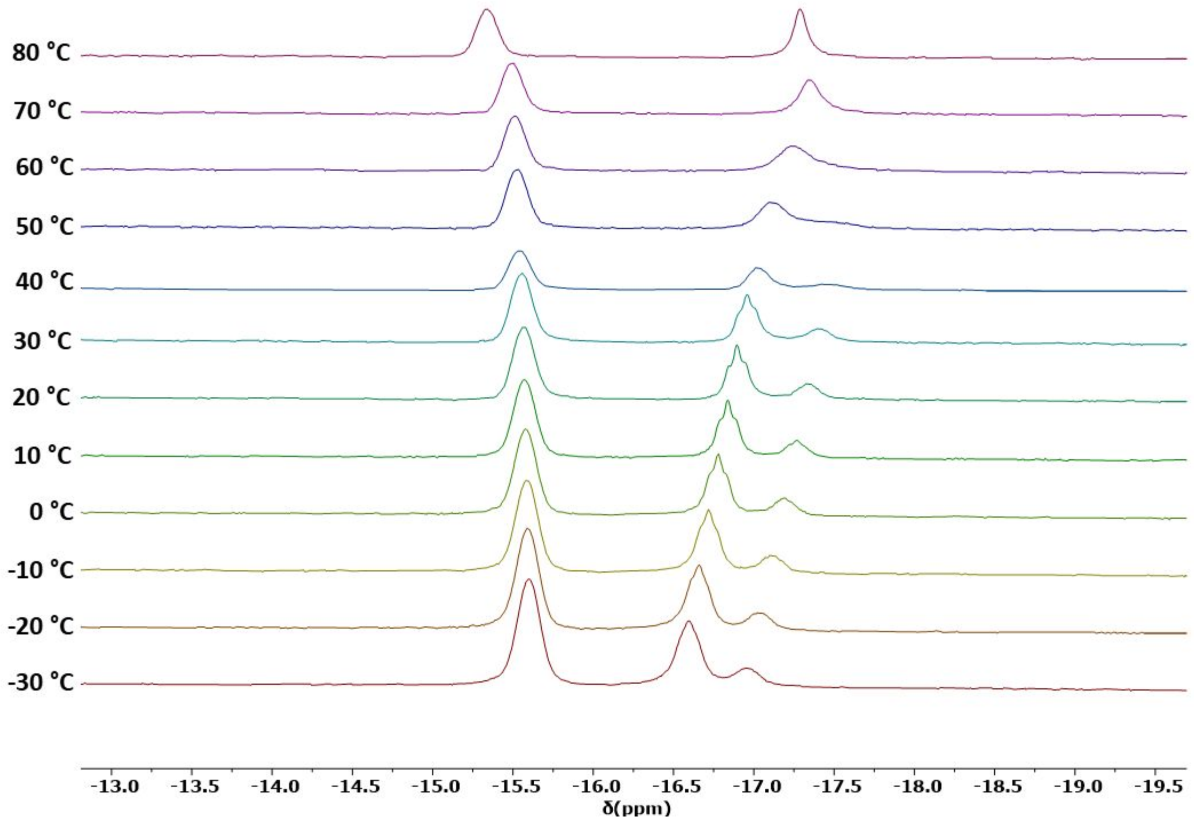

Figure S49. Variable-temperature $^{11}B\{^{1}H\}$ NMR spectra of $\mathbf{3_Y}$ from –30 °C to 80 °C in chlorobenzene-D_5.

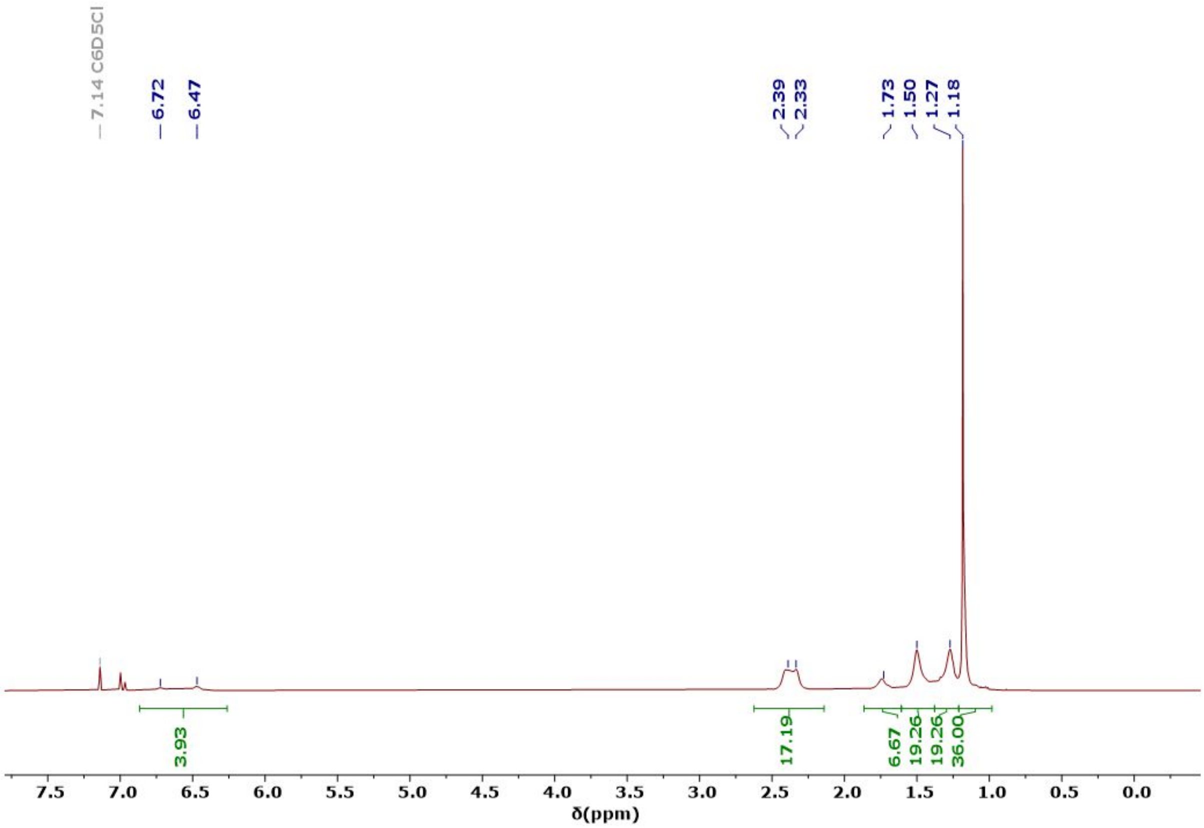

Figure S50. ^{1}H NMR spectrum of $\mathbf{3_{Lu}}$ in chlorobenzene-D_5 at 30 °C.

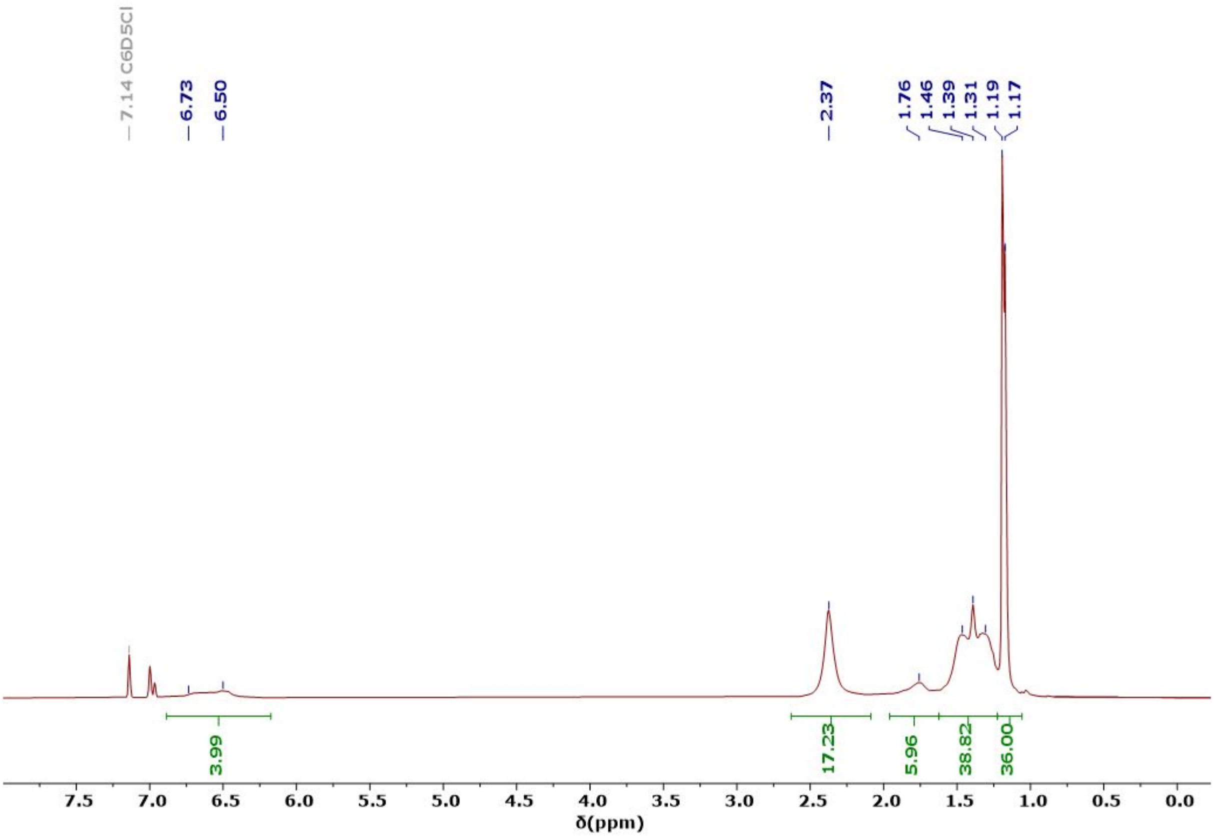

Figure S51. $^{1}H\{^{11}B\}$ NMR spectrum of $\mathbf{3}_{\mathbf{Lu}}$ in chlorobenzene-D_5 at 30 °C.

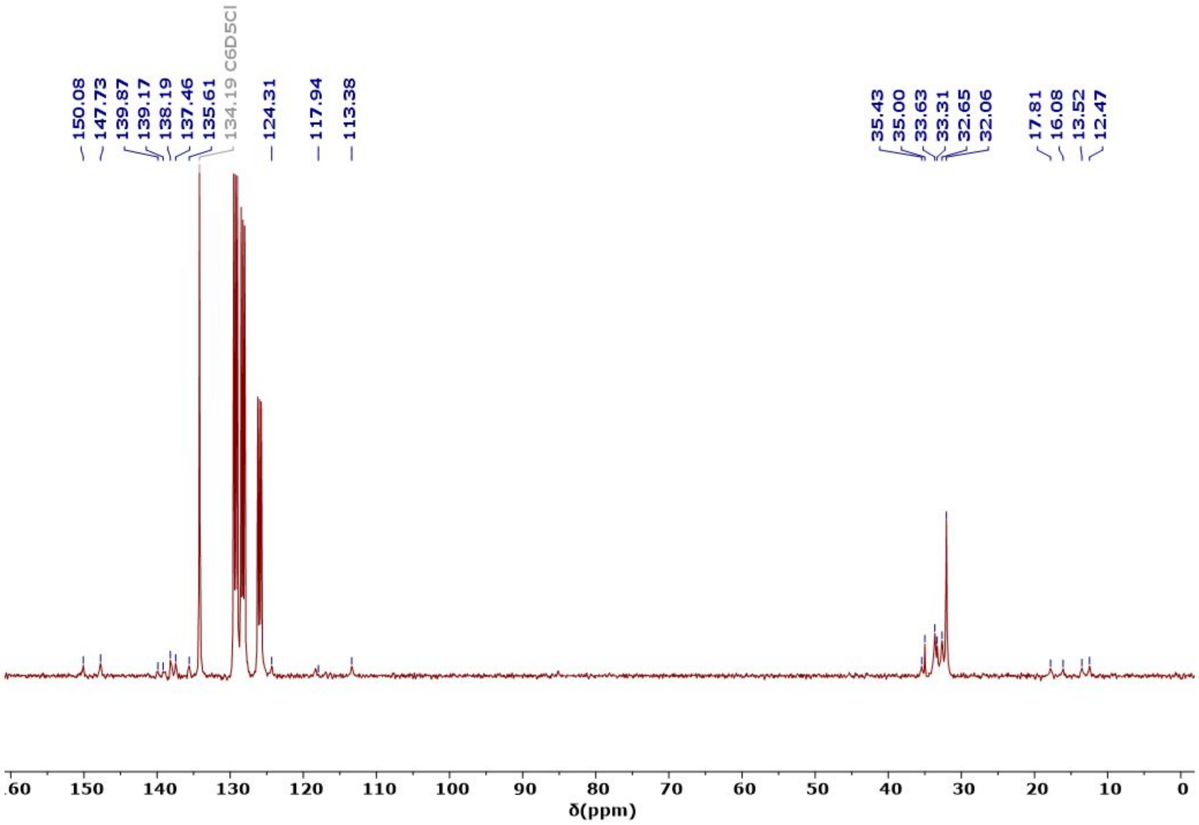

Figure S52. $^{13}C\{^{1}H\}$ NMR spectrum of $\mathbf{3_{Lu}}$ in chlorobenzene-D_5 at 30 °C.

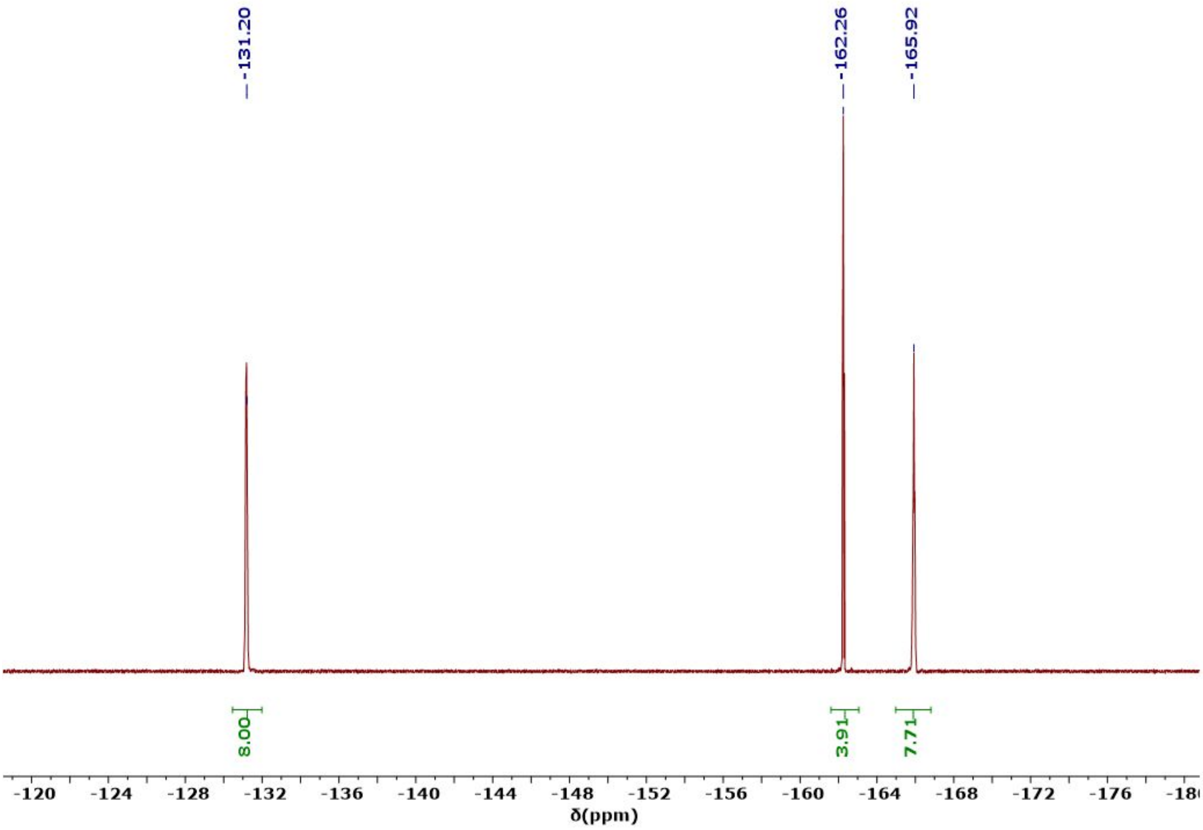

Figure S53. ^{19}F NMR spectrum of $\mathbf{3_{Lu}}$ in chlorobenzene-D_5 at 30 °C.

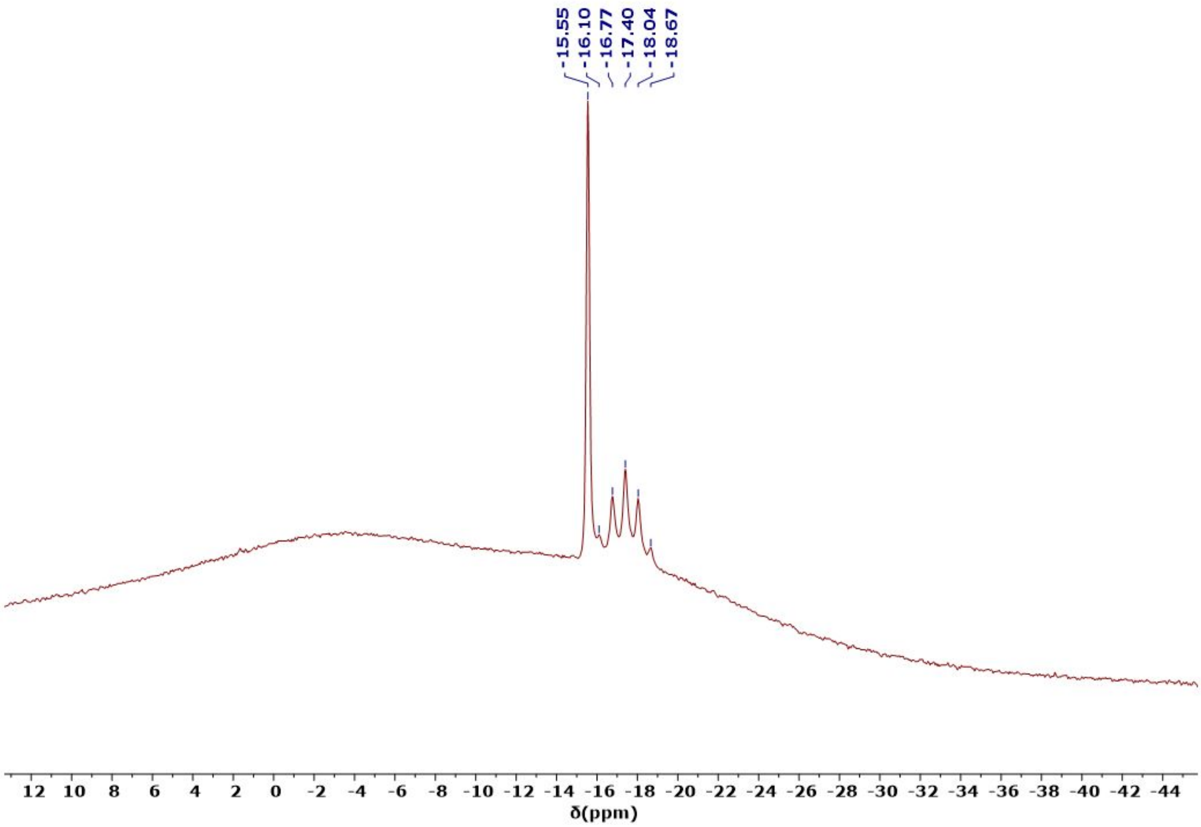

Figure S54. ^{11}B NMR spectrum of $\mathbf{3_{Lu}}$ in chlorobenzene-D$_5$ at 30 °C.

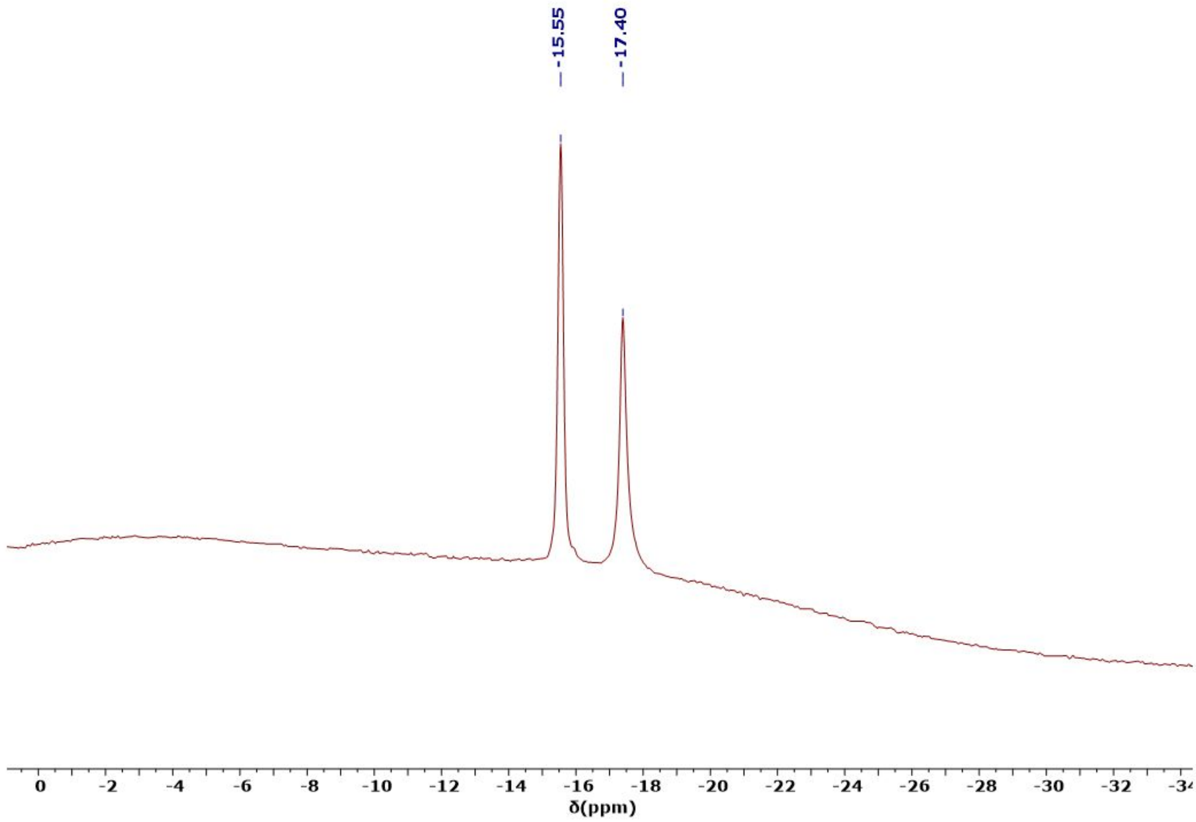

Figure S55. $^{11}B\{^{1}H\}$ NMR spectrum of $\mathbf{3_{Lu}}$ in chlorobenzene-D_5 at 30 °C.

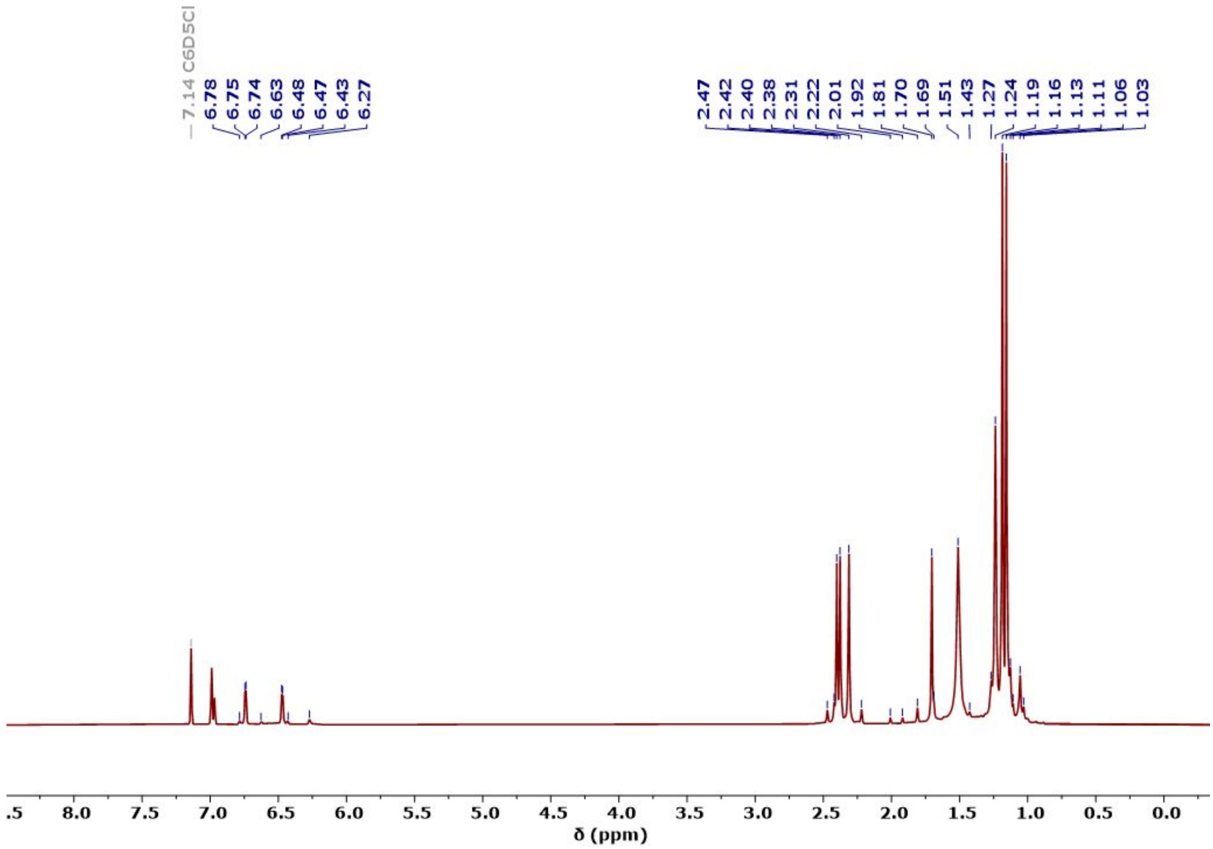

Figure S56. ^{1}H NMR spectrum of $\mathbf{3_{Lu}}$ in chlorobenzene-D$_5$ at –30 °C.

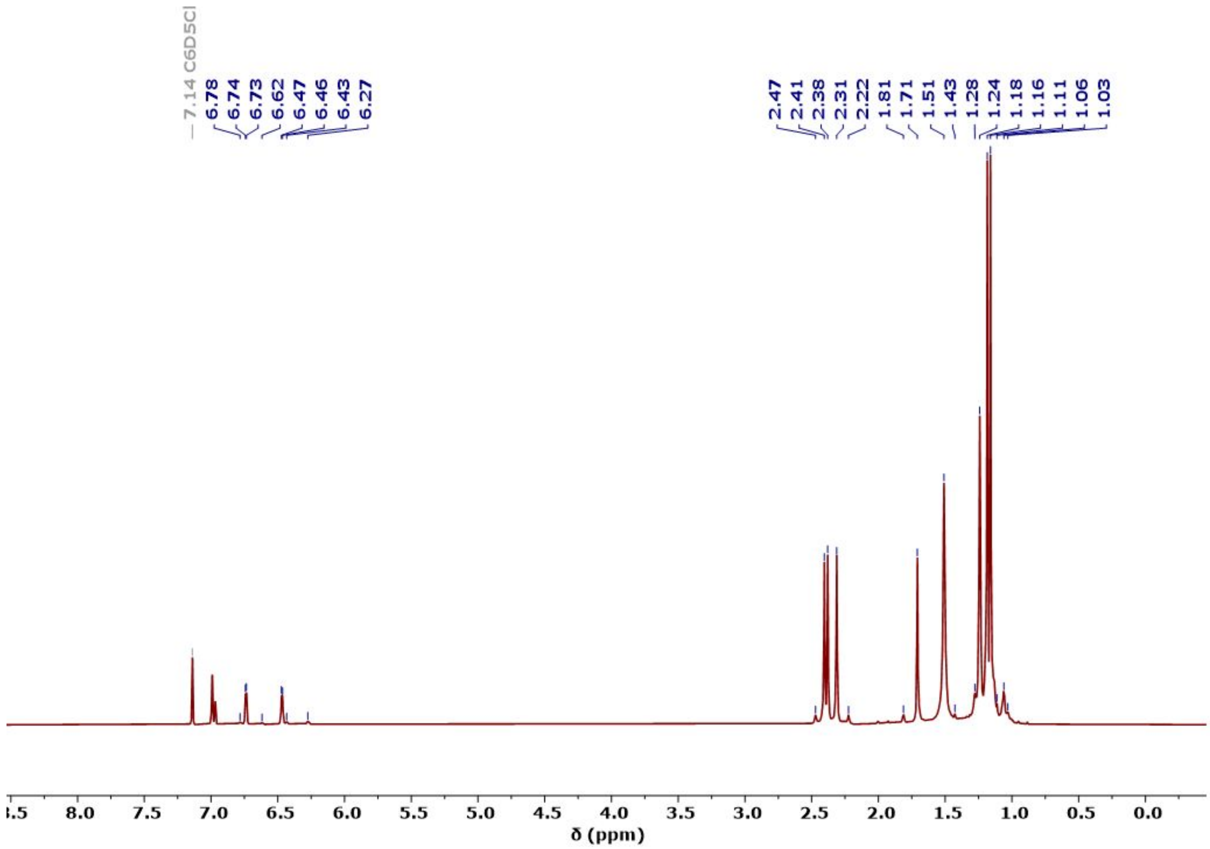

Figure S57. ^{1}H NMR spectrum of $\mathbf{3_{Lu}}$ in chlorobenzene-D$_5$ at –20 °C.

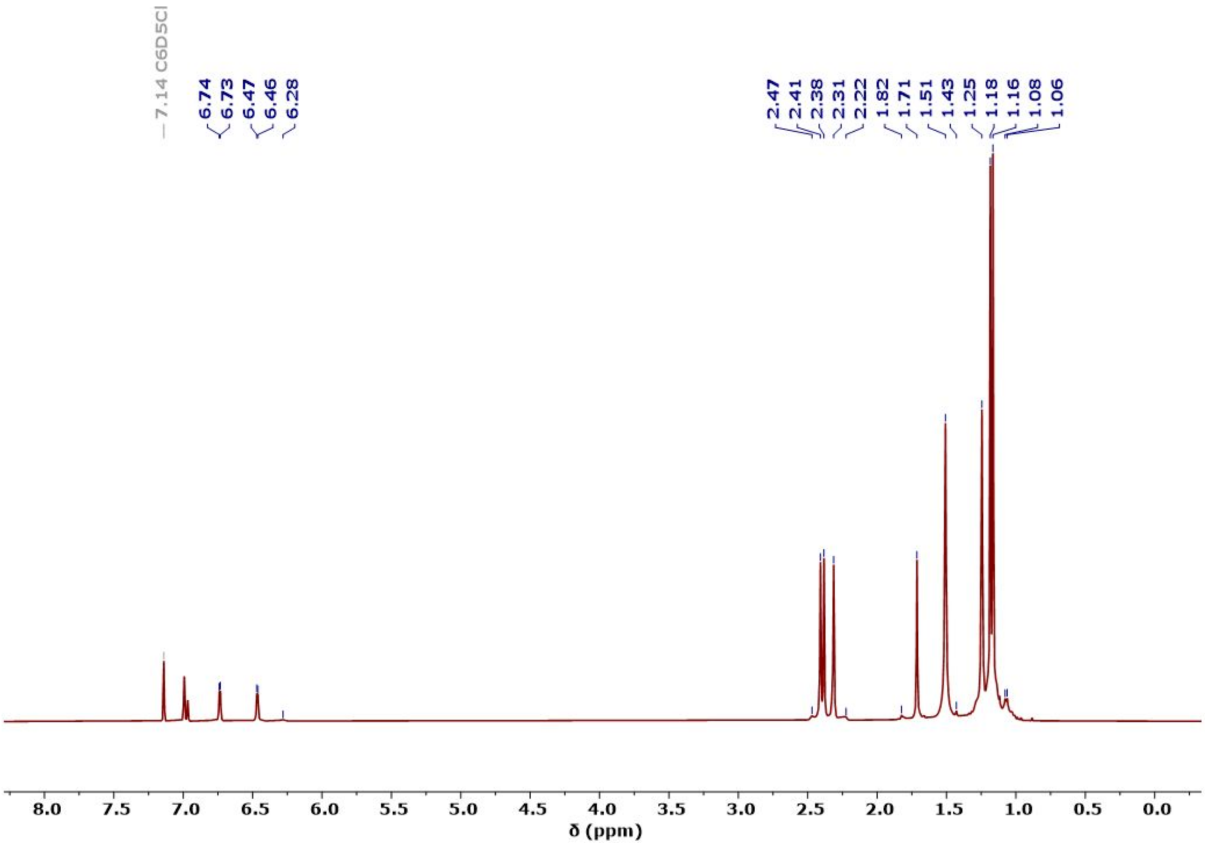

Figure S58. ^{1}H NMR spectrum of $\mathbf{3_{Lu}}$ in chlorobenzene-D$_5$ at –10 °C.

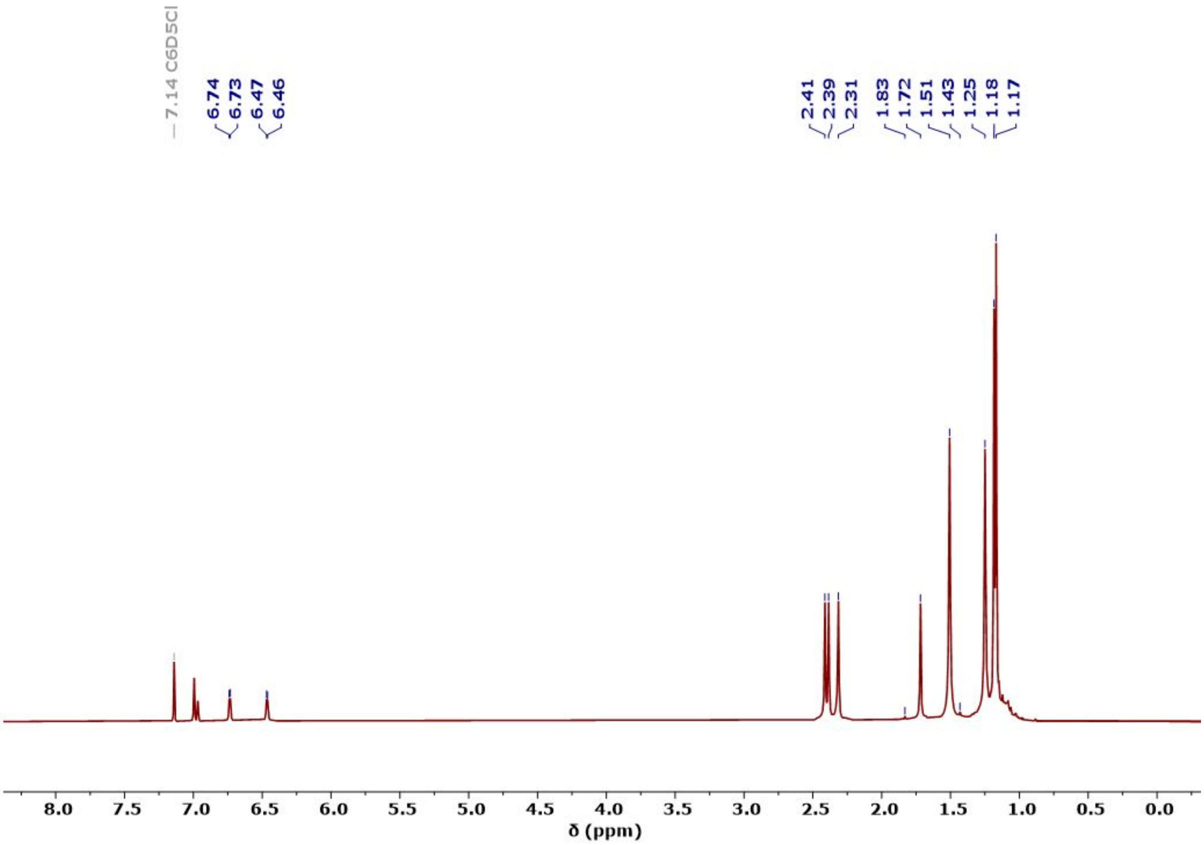

Figure S59. ^{1}H NMR spectrum of $\mathbf{3_{Lu}}$ in chlorobenzene-D_5 at 0 °C.

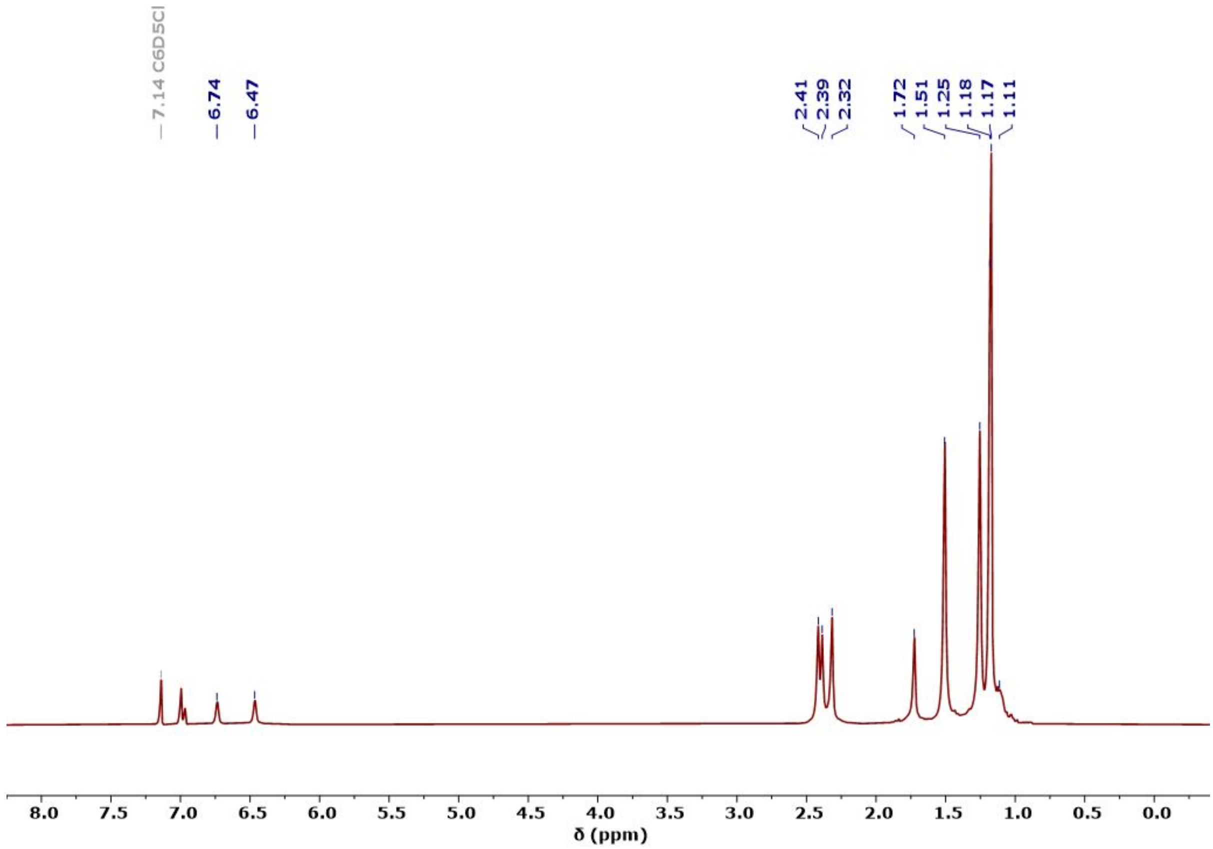

Figure S60. ^1H NMR spectrum of $\mathbf{3_{Lu}}$ in chlorobenzene-D_5 at 10 °C.

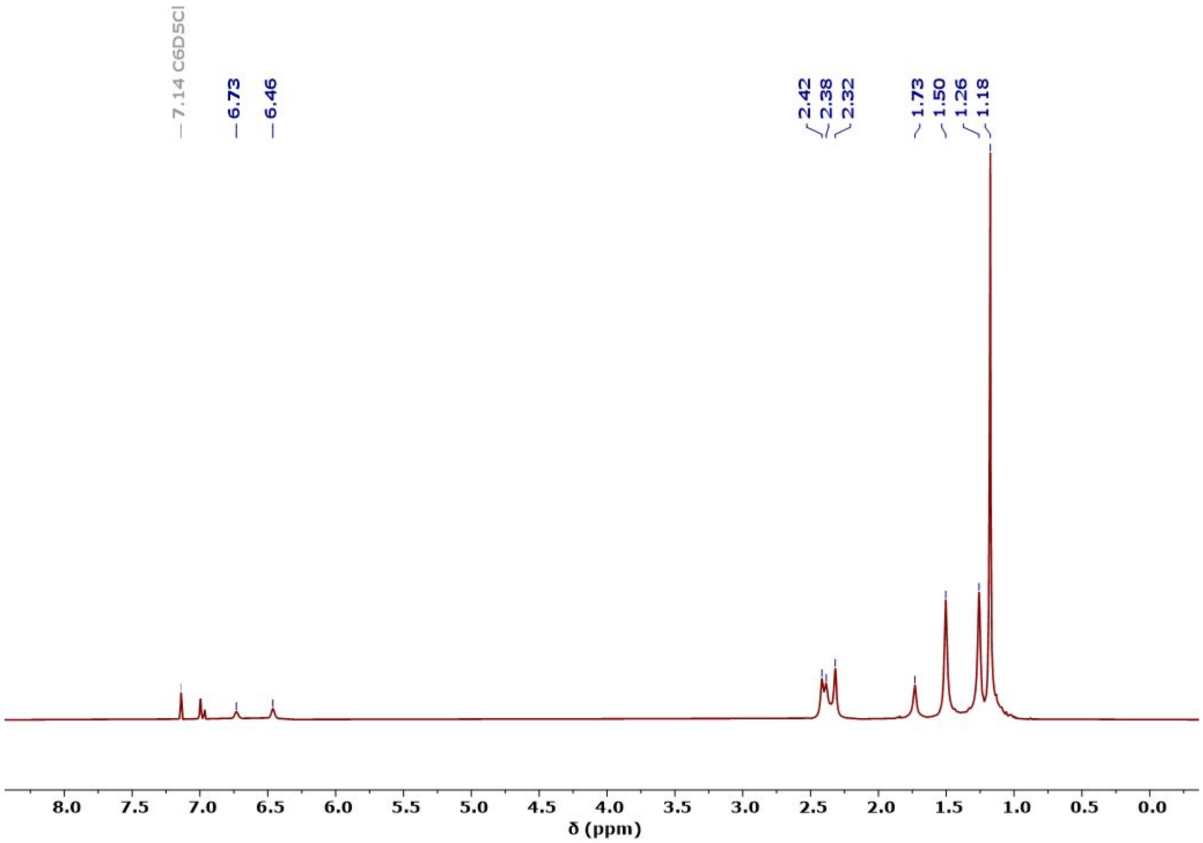

Figure S61. ^{1}H NMR spectrum of $\mathbf{3_{Lu}}$ in chlorobenzene-D_5 at 20 °C.

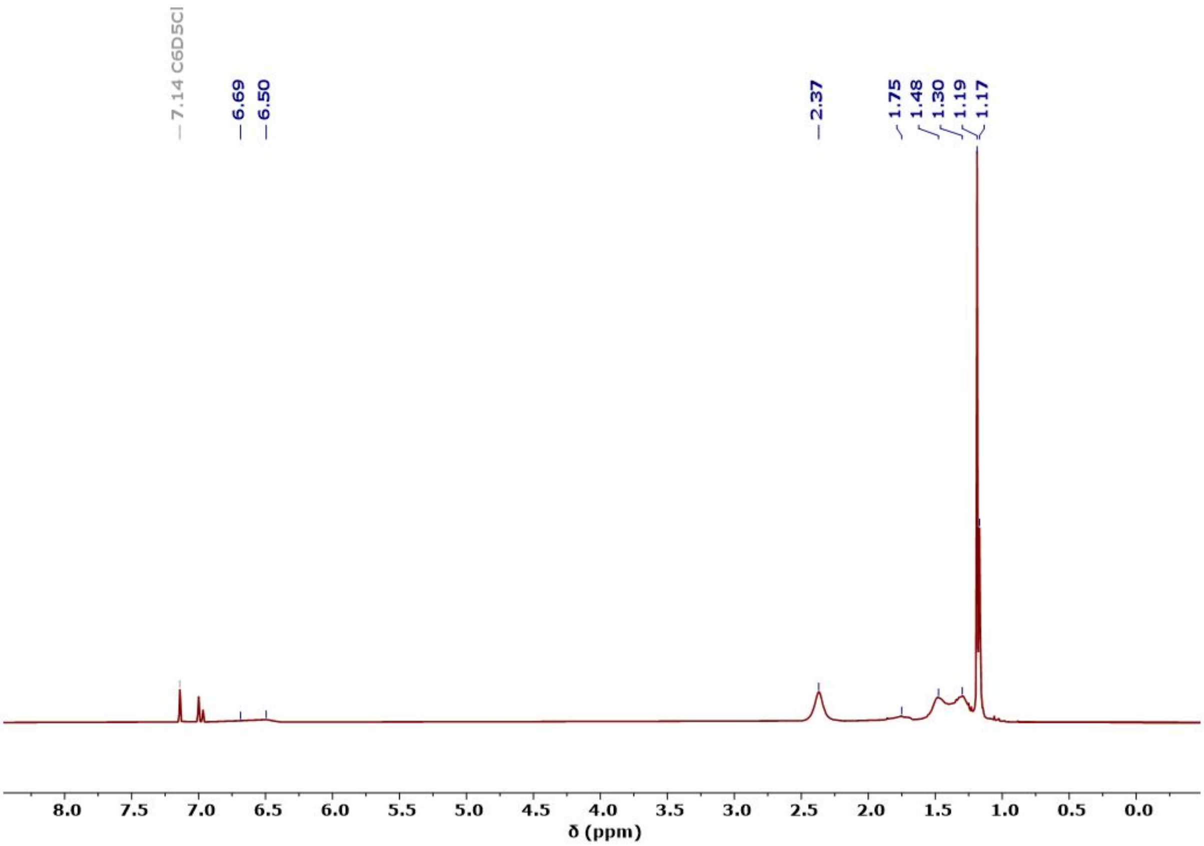

Figure S62. ^{1}H NMR spectrum of $\mathbf{3_{Lu}}$ in chlorobenzene-D_5 at 40 °C.

Fi

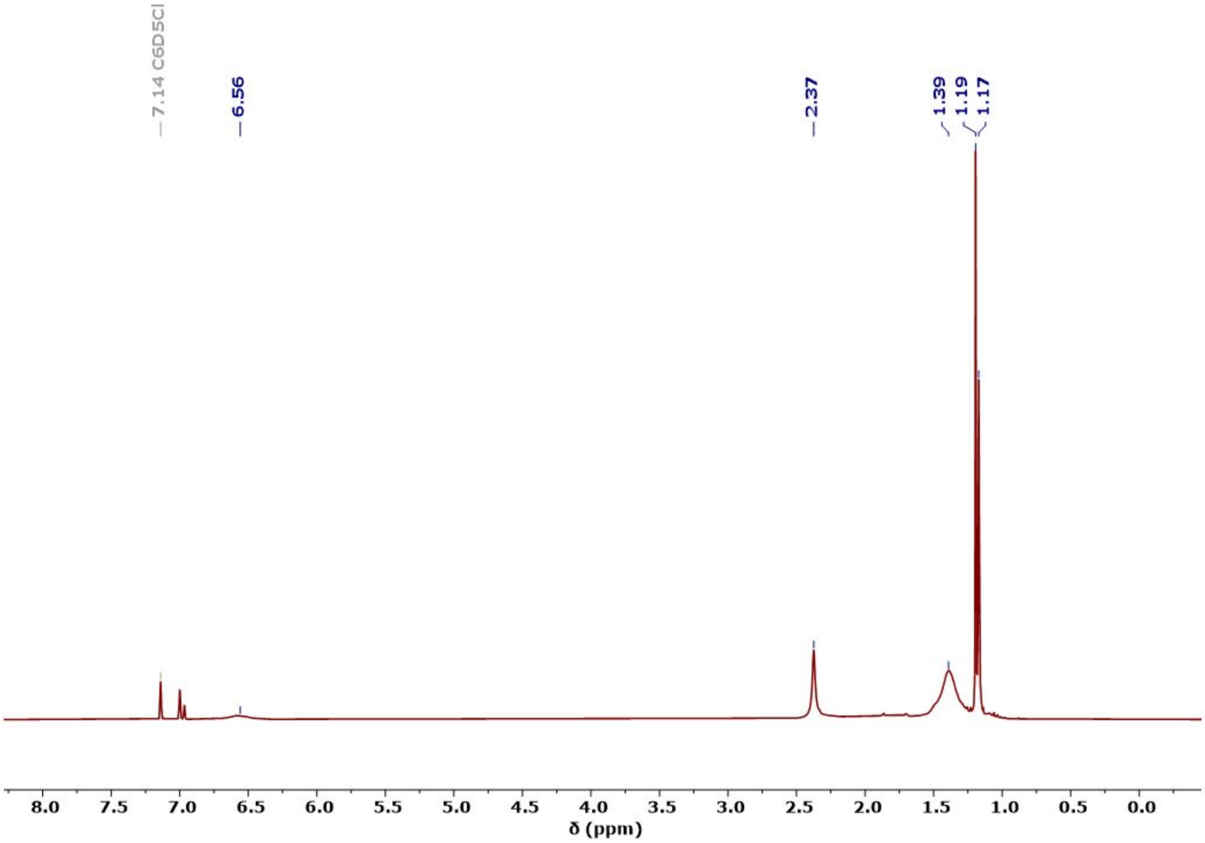

Figure S63. ^{1}H NMR spectrum of $\mathbf{3_{Lu}}$ in chlorobenzene-D_5 at 50 °C.

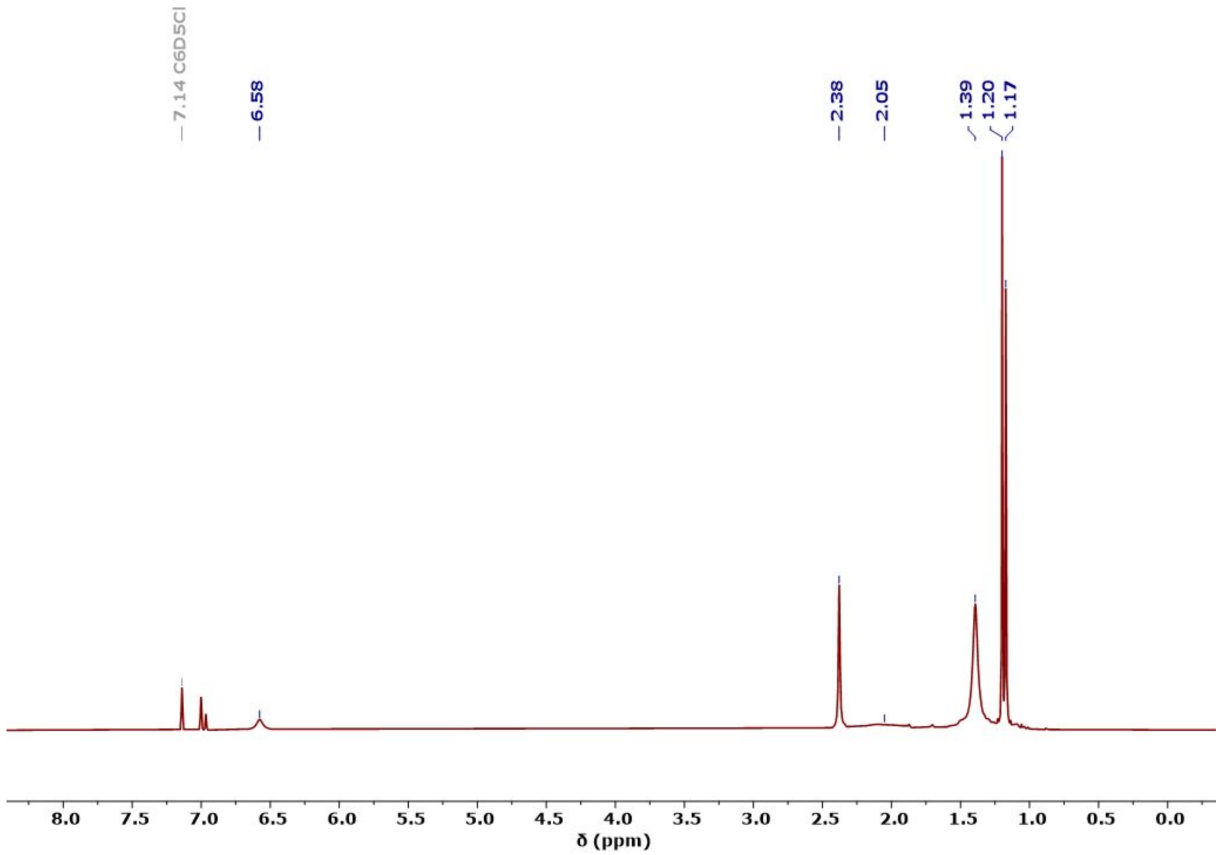

Figure S64. ^{1}H NMR spectrum of $\mathbf{3_{Lu}}$ in chlorobenzene-D_5 at 60 °C.

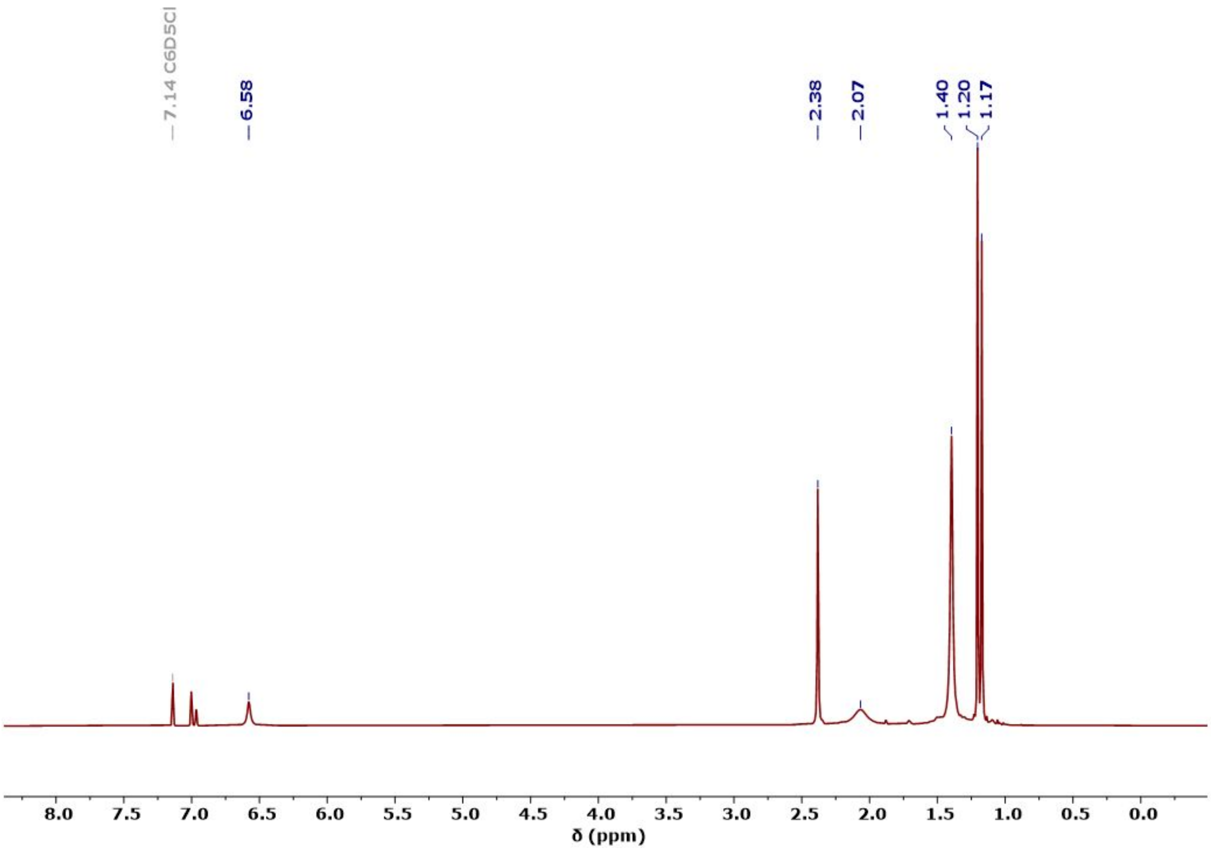

Figure S65. ^{1}H NMR spectrum of $\mathbf{3}_{\mathbf{Lu}}$ in chlorobenzene-D$_5$ at 70 °C.

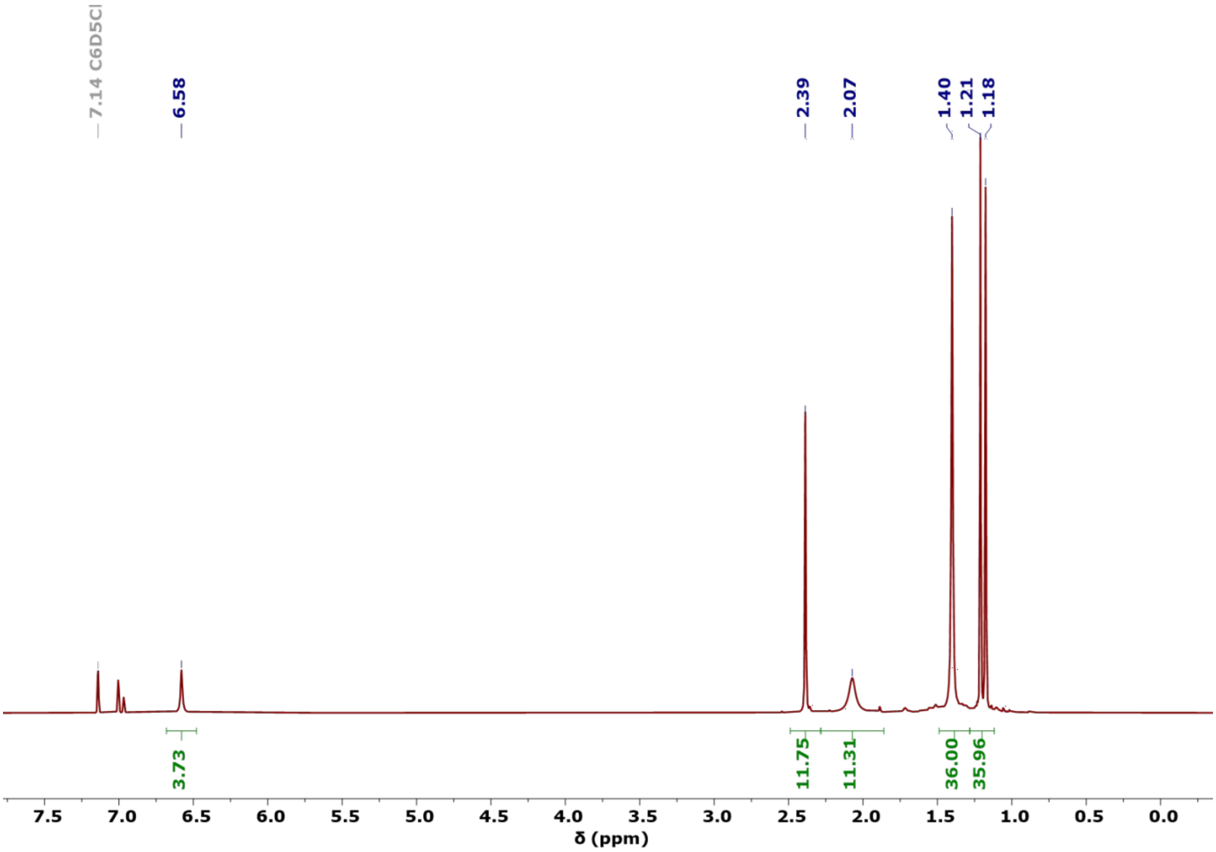

Figure S66. ^{1}H NMR spectrum of $\mathbf{3_{Lu}}$ in chlorobenzene-D$_5$ at 80 °C.

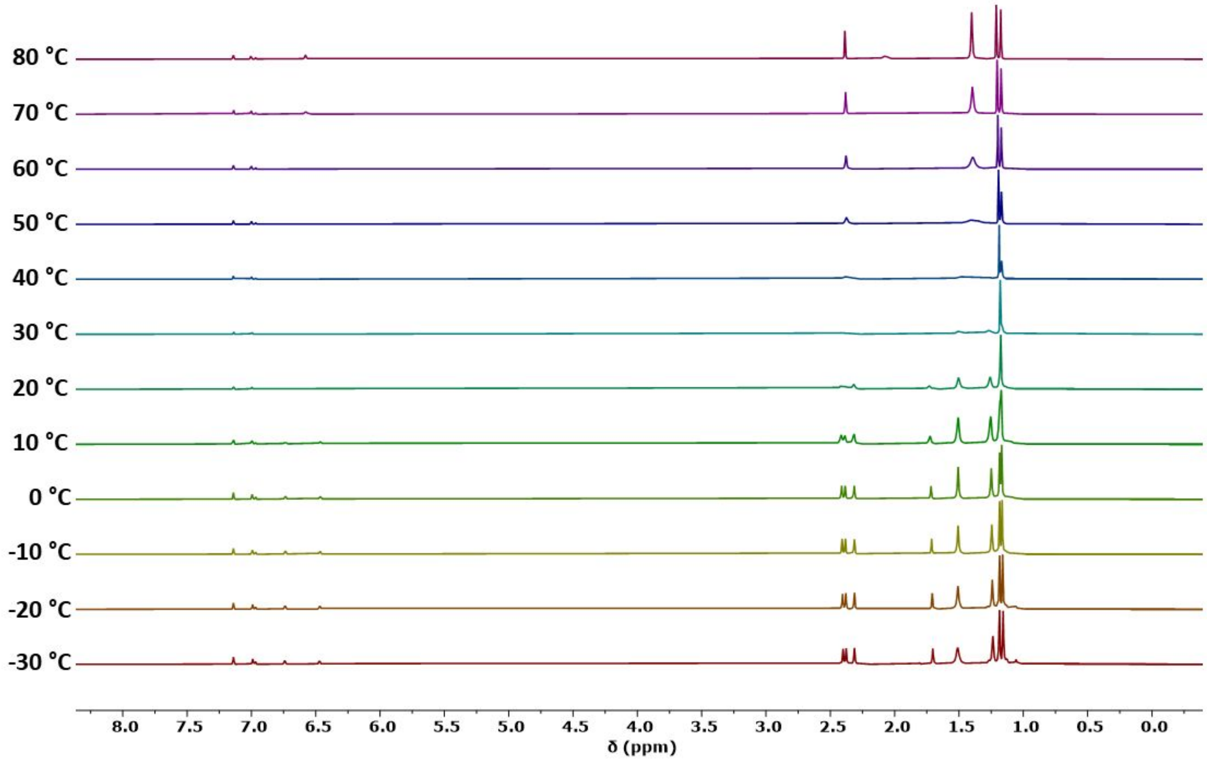

Figure S67. Variable-temperature ^{1}H NMR spectra of $\mathbf{3_{Lu}}$ in chlorobenzene-D_5 from –30 °C to 80 °C.

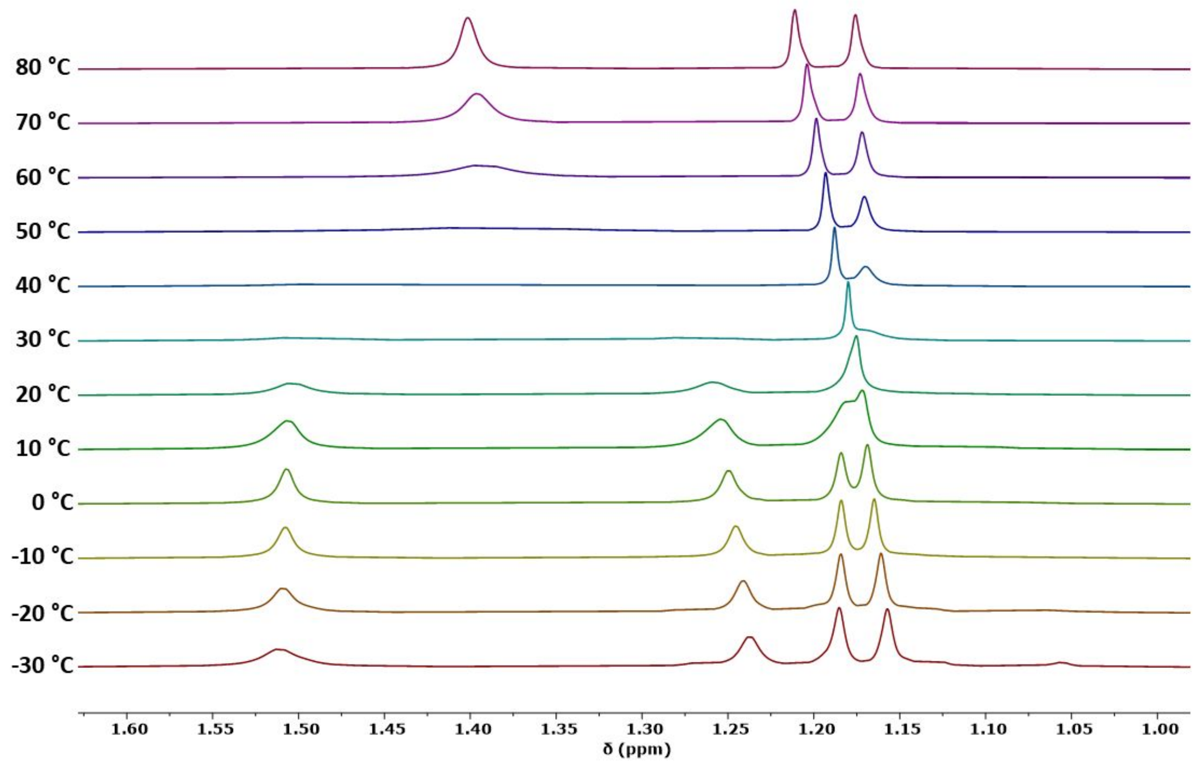

Figure S68. Variable-temperature ^{1}H NMR spectra of $\mathbf{3_{Lu}}$ in chlorobenzene-D_5 in the range 1.0-1.6 ppm from –30 °C to 80 °C.

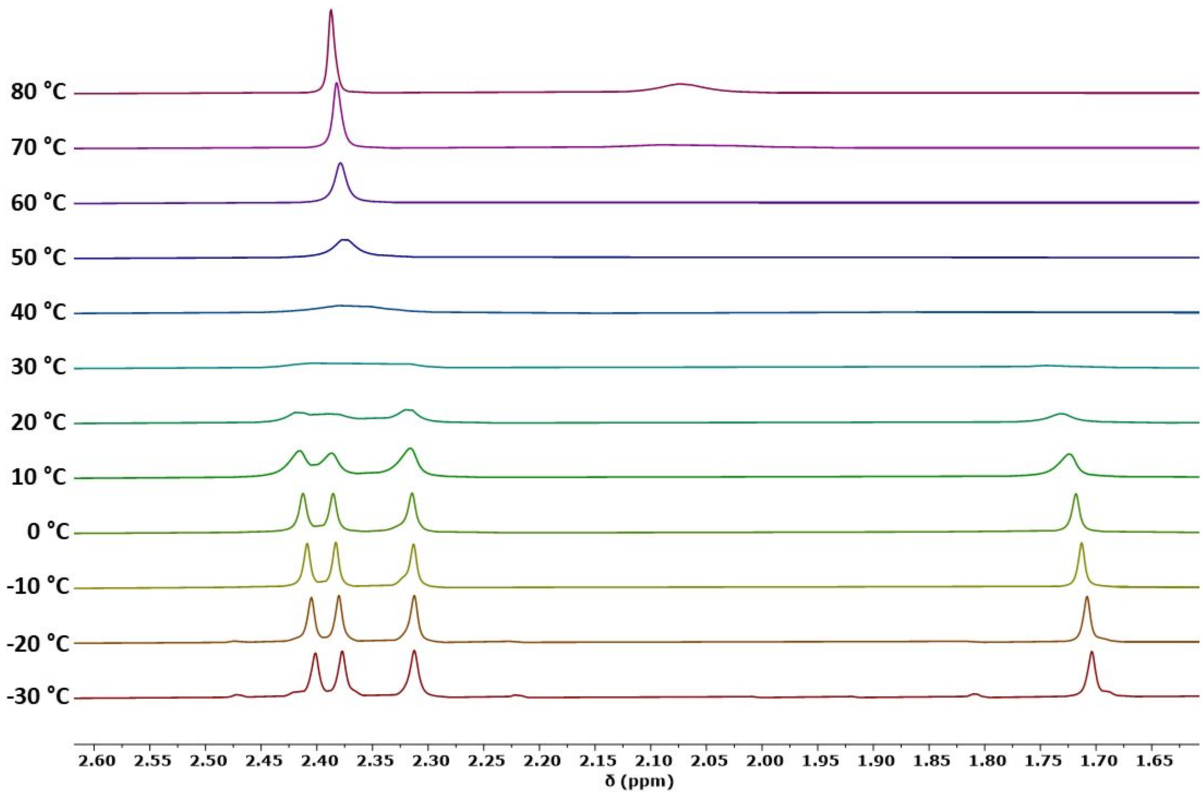

Figure S69. Variable-temperature ^{1}H NMR spectra of $\mathbf{3_{Lu}}$ in chlorobenzene-D$_5$ in the range 1.6-2.6 ppm range from –30 °C to 80 °C.

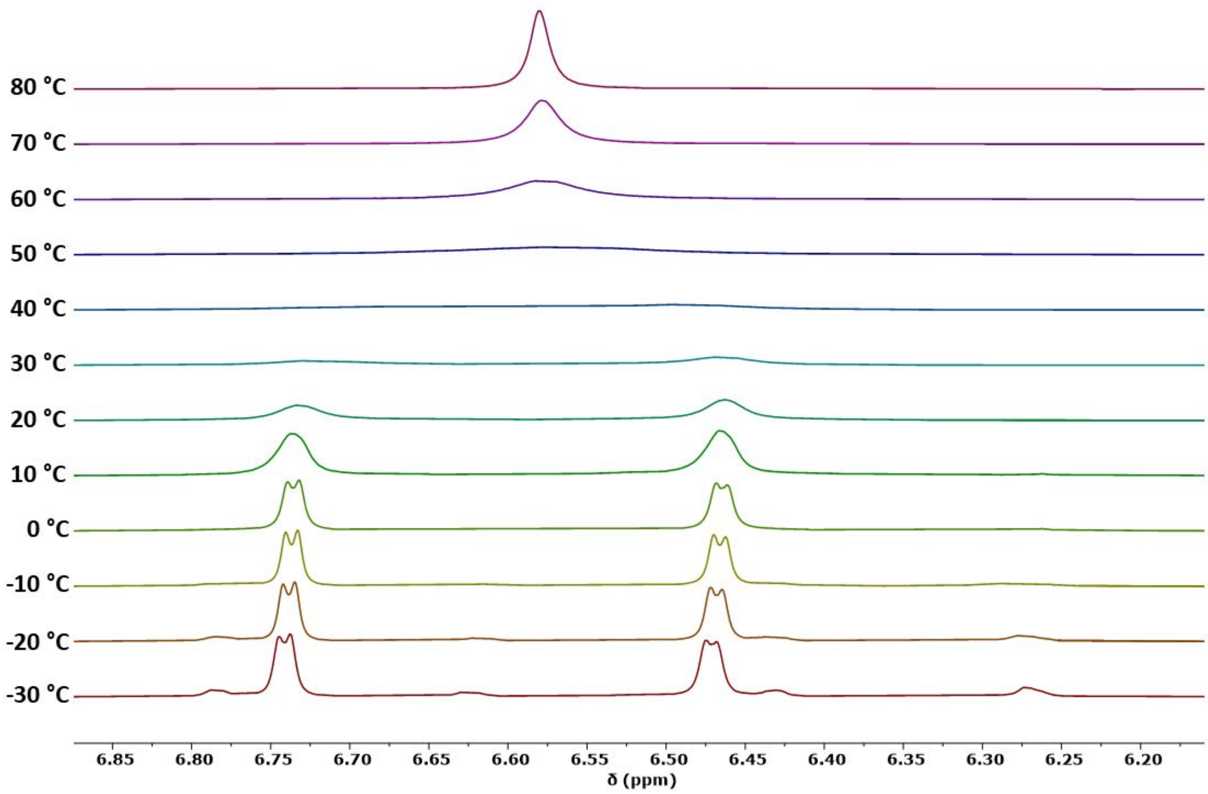

Figure S70. Variable-temperature ^{1}H NMR spectra of $\mathbf{3_{Lu}}$ in chlorobenzene-D_5 in the range 6.2-6.9 ppm range from –30 °C to 80 °C.

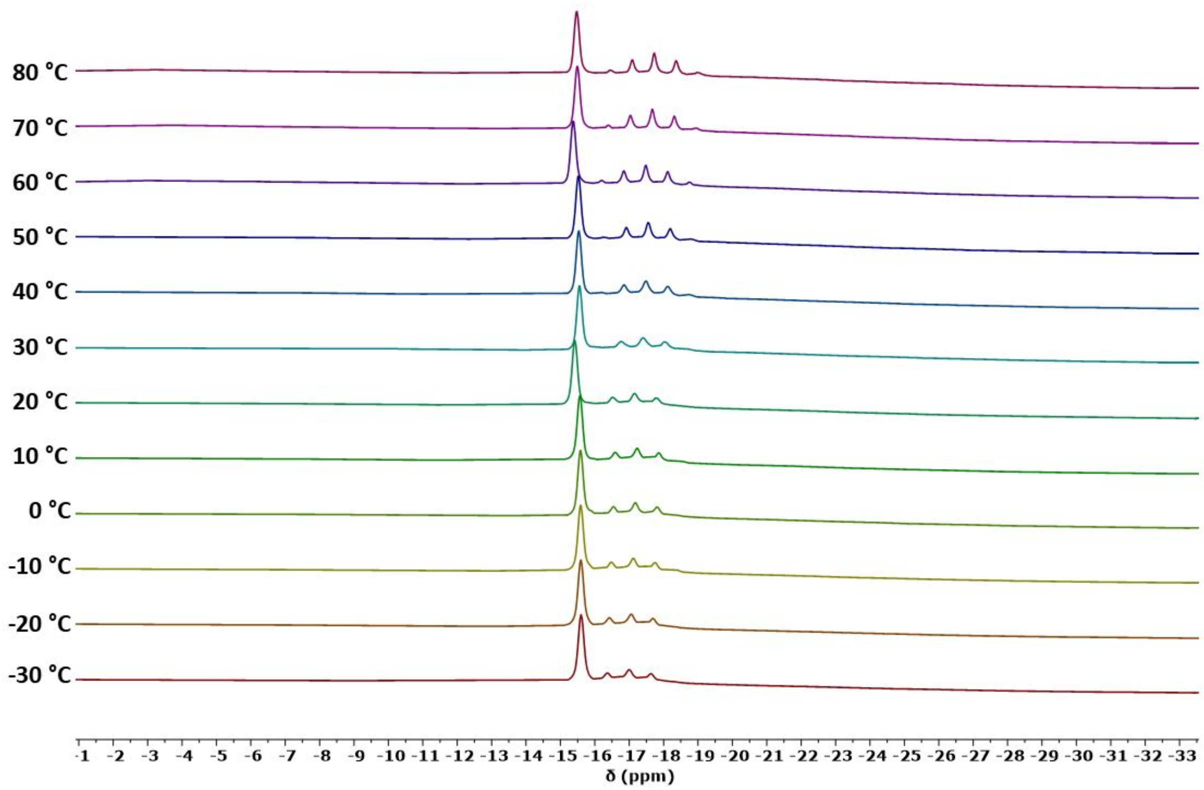

Figure S71. Variable-temperature ^{11}B NMR spectra of $\mathbf{3_{Lu}}$ in chlorobenzene-D$_5$ from –30 °C to 80 °C.

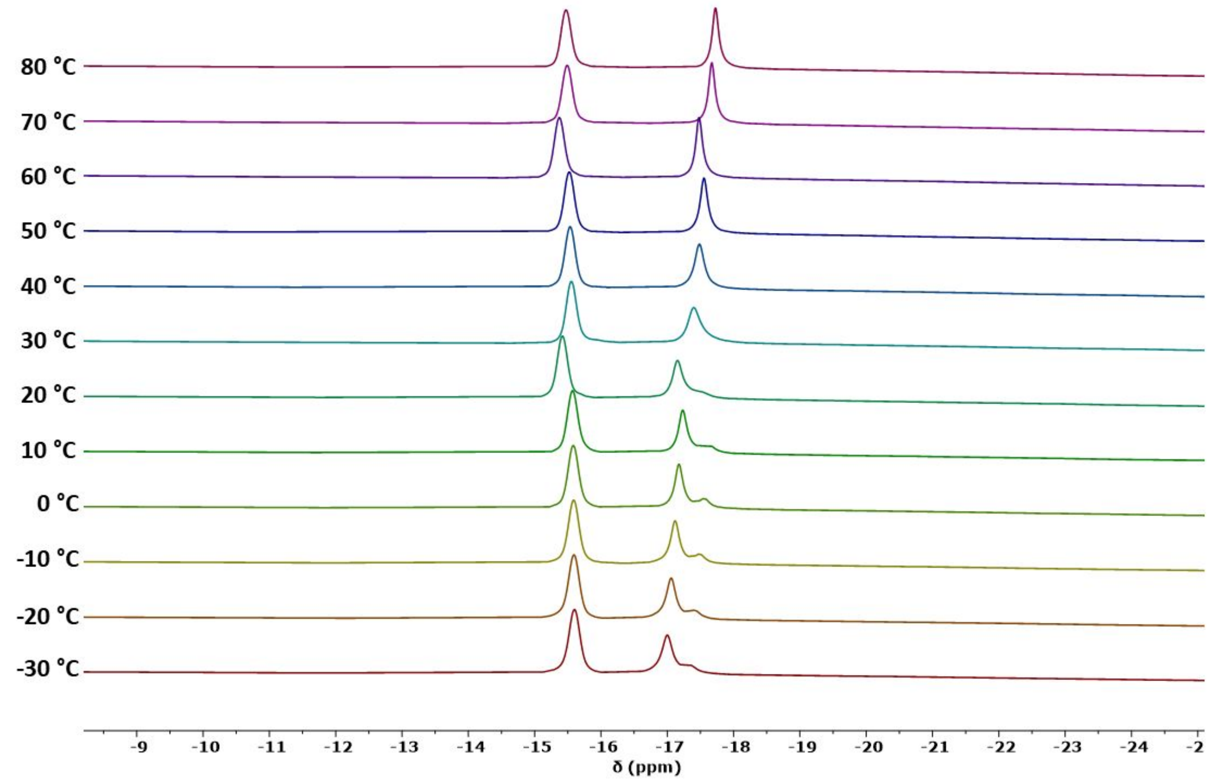

Figure S72. Variable-temperature $^{11}B\{^{1}H\}$ NMR spectra of $\mathbf{3_{Lu}}$ in chlorobenzene-D_5 from –30°C to 80°C.

Eyring Analysis of 3_Y and 3_{Lu}

Rotational barriers, $\Delta G^‡$, of the Cp^{ttt} ligands in $\mathbf{3_Y}$ and $\mathbf{3_{Lu}}$ were estimated directly using the respective coalescence temperatures, T_c, of the Cp^{ttt} methine proton signals (T_C = 333 K in $\mathbf{3_Y}$; 313 K in $\mathbf{3_{Lu}}$) (Table S1).[4] Rate constants were also determined for a simple two-site exchange process from the Cp^{ttt} methine proton chemical shifts and FWHM, in both slow and fast exchange regimes as well as at the coalescence temperatures. The method assumes a transmission coefficient of unity, and enthalpies and entropies of activation that are invariant with temperature. Plots of of $\ln(k/T)$ vs. T^{-1} yielded linear relationships (R^2 = 0.992 for $\mathbf{3_Y}$, R^2 = 0.995 for $\mathbf{3_{Lu}}$) from which the $\Delta H^‡$ and $\Delta S^‡$ could be estimated (Figure S73). Due to the relatively small number of temperatures measured, estimates for $\Delta S^‡$ are unreliable. However, the large negative values are indicative of an ordered transition state which is consistent with the non-dissociative rotational process.

Table S7. Thermodynamic parameters determined for $\mathbf{3_Y}$ and $\mathbf{3_{Lu}}$ via an Eyring analysis.

	$\mathbf{3_Y}$	$\mathbf{3_{Lu}}$
$\Delta G^‡$ / kJmol^{-1}	67.47	62.47
$\Delta H^‡$ / kJmol^{-1}	128.48	89.62
$\Delta S^‡$ / JK^{-1}mol^{-1}	–699.61	–795.57

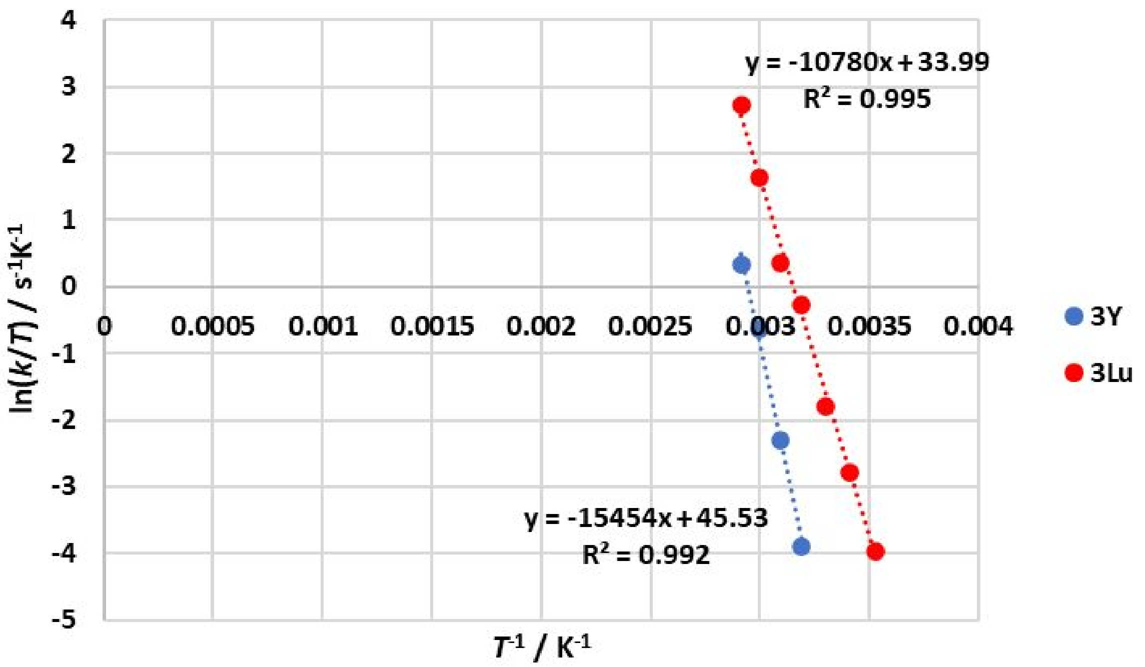

Figure S73. Plots of $\ln(k/T)$ vs. T^{-1} for the exchange of Cp^{ttt} methine protons in $\mathbf{3_Y}$ and $\mathbf{3_{Lu}}$, with equations and R^2 values describing the linear trendlines.

Magnetic property measurements

Magnetic measurements were recorded on a Quantum Design MPMS-XL7 SQUID magnetometer equipped with a 7 T magnet. The samples were restrained in eicosane and sealed in 7 mm NMR tubes. Direct current (DC) magnetic susceptibility measurements were performed on polycrystalline samples of $\mathbf{1}_{\mathbf{Dy}}$ (26.2 mg), $\mathbf{2}_{\mathbf{Dy}}$ (37.2 mg) and [$\mathbf{3}_{\mathbf{Dy}}$][$B(C_6F_5)_4$] (23.7 mg) in the temperature range 1.9-300 K and using an applied field of 1000 Oe. Alternating current (AC) susceptibility measurements were performed using an AC field of 3 Oe in zero DC field. Diamagnetic corrections were made using Pascal's constants for all the constituent atoms.[7] Measurements on the diluted sample were performed using a polycrystalline sample of **Dy@3**$_{\mathbf{Y}}$ (51.2 mg).

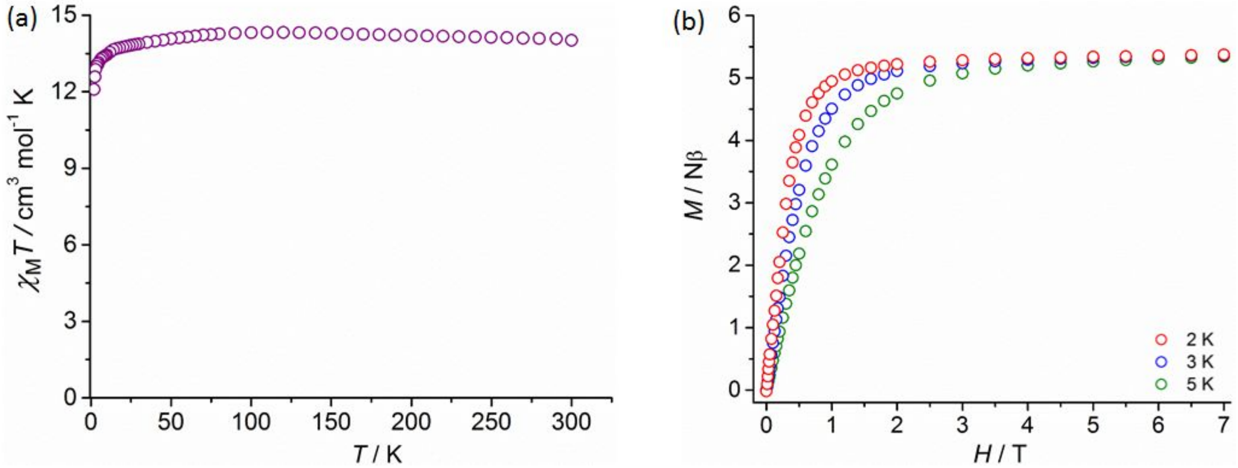

Figure S74. (a) Magnetic susceptibility as a function of temperature and (b) isothermal field dependence of magnetization for $\mathbf{1}_{\mathbf{Dy}}$. The $\chi_M T$ values are 14.01 cm^3 K mol^{-1} at 300 K and 12.09 cm^3 K mol^{-1} at 2 K.

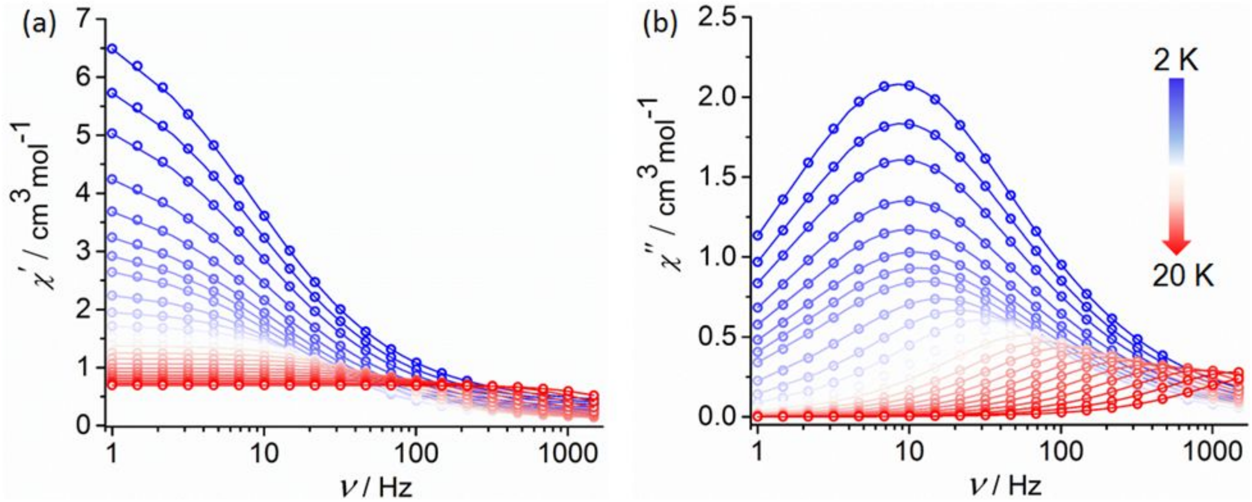

Figure S75. (a) In-phase and (b) out-of-phase (b) AC susceptibility as a function of frequency at T = 2-20 K under zero DC field for $\mathbf{1}_{\mathbf{Dy}}$.

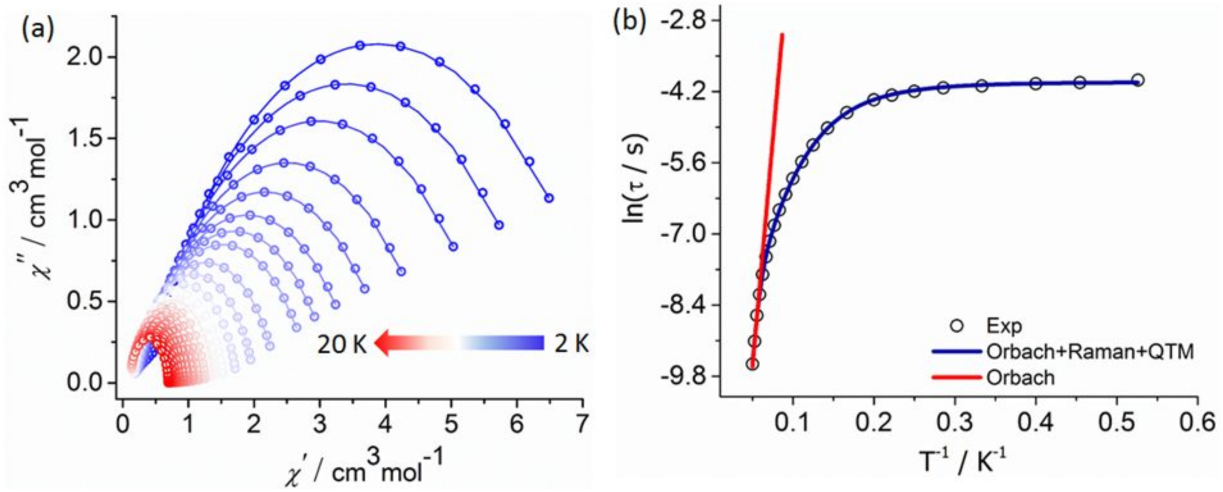

Figure S76. (a) Cole-Cole plot and (b) temperature-dependence of the relaxation time for $\mathbf{1_{Dy}}$, where solid lines are fits to the data using equations 1 and 2. The parameters are stated in Table S8.

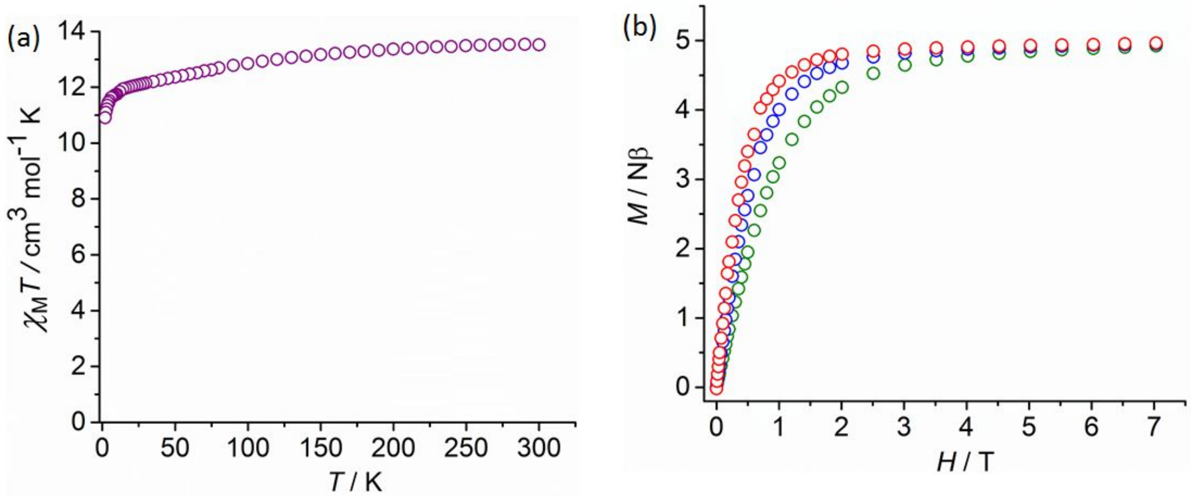

Figure S77. (a) Magnetic susceptibility as a function of temperature and (b) isothermal field dependence of magnetization for $\mathbf{2_{Dy}}$. The $\chi_M T$ values are 13.52 cm^3 K mol^{-1} at 300 K and 10.90 cm^3 K mol^{-1} at 2 K.

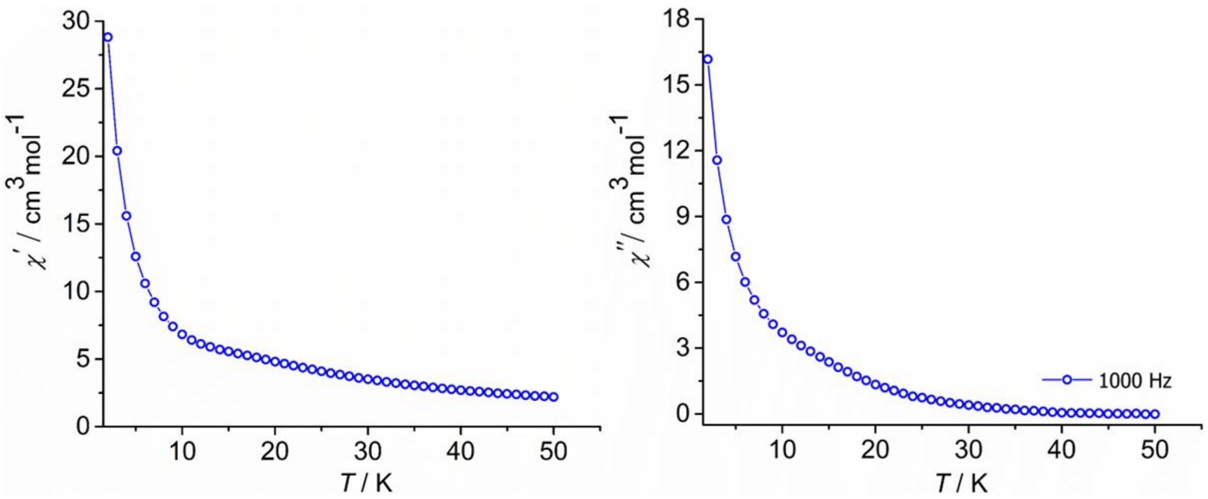

Figure S78. In-phase and (a) and out-of-phase (b) AC susceptibility as a function of temperature at 1000 Hz frequency under zero DC field for $\mathbf{2}_{\mathbf{Dy}}$.

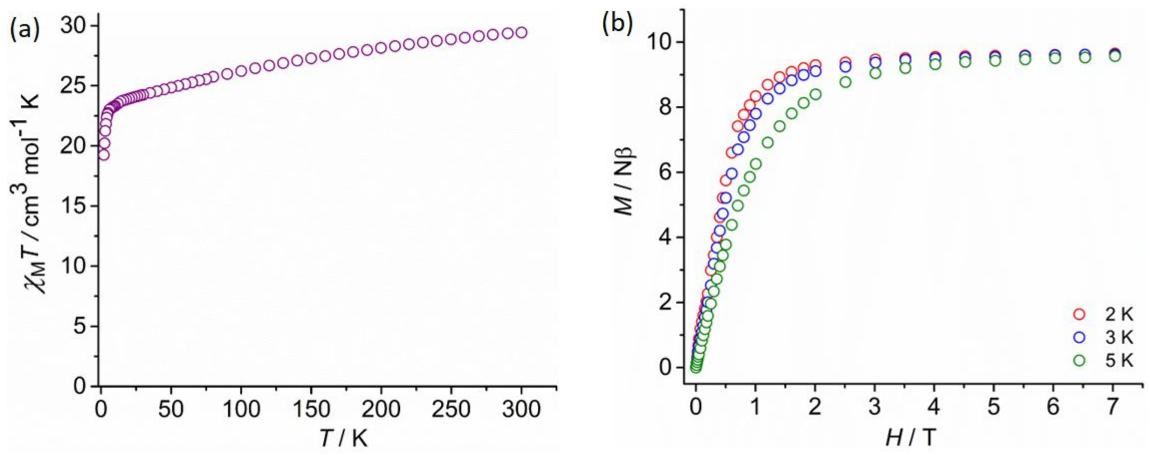

Figure S79. (a) Magnetic susceptibility as a function of temperature and (b) isothermal field dependence of magnetization for $[\mathbf{3}_{\mathbf{Dy}}][B(C_6F_5)_4]$. The $\chi_M T$ values are 29.43 cm^3 K mol^{-1} at 300 K and 19.25 cm^3 K mol^{-1} at 2 K.

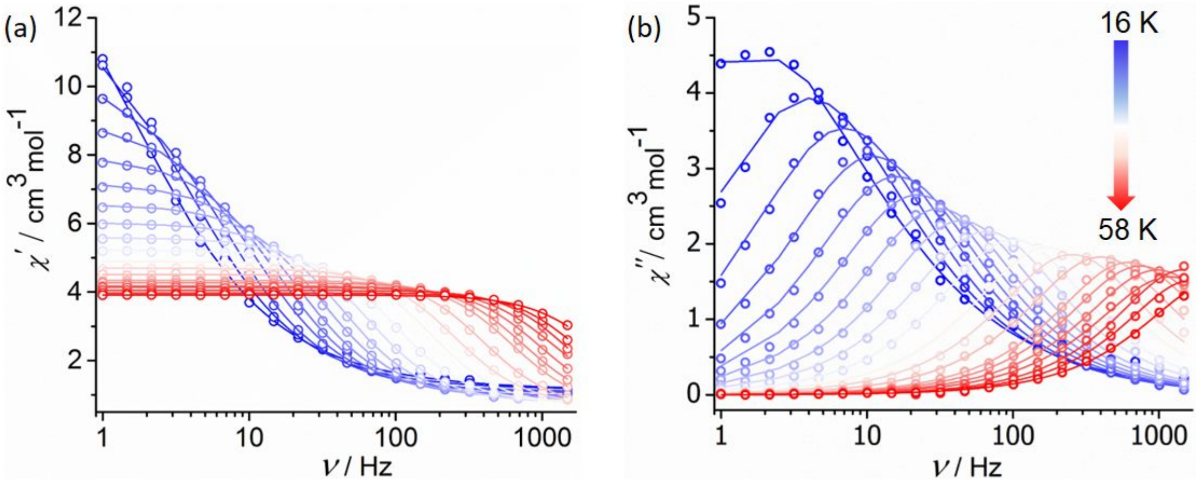

Figure S80. (a) In-phase and (b) out-of-phase (b) AC susceptibility as a function of frequency at T = 2-58 K under zero DC field for [$\mathbf{3}_{\mathbf{Dy}}$][B(C_6F_5)$_4$].

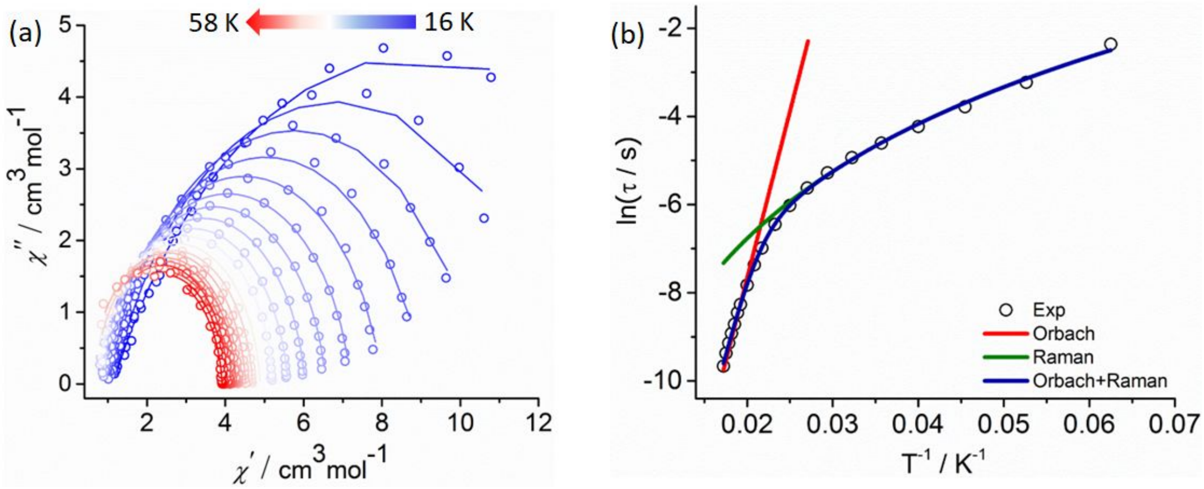

Figure S81. (a) Cole-Cole plot and (b) temperature-dependence of the relaxation time for [$\mathbf{3}_{\mathbf{Dy}}$][B(C_6F_5)$_4$], where solid lines are fits to the data using equations 1 and 2. The parameters are stated in Table S9.

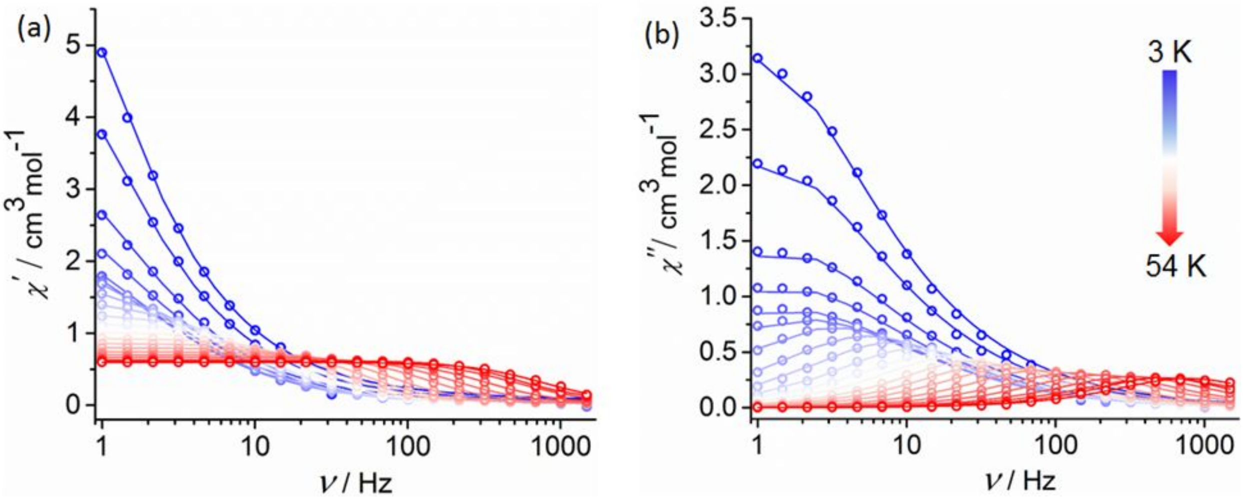

Figure S82. (a) In-phase and (b) out-of-phase (b) AC susceptibility as a function of frequency at T = 2-54 K under zero DC field for [**Dy@3$_Y$**][$B(C_6F_5)_4$].

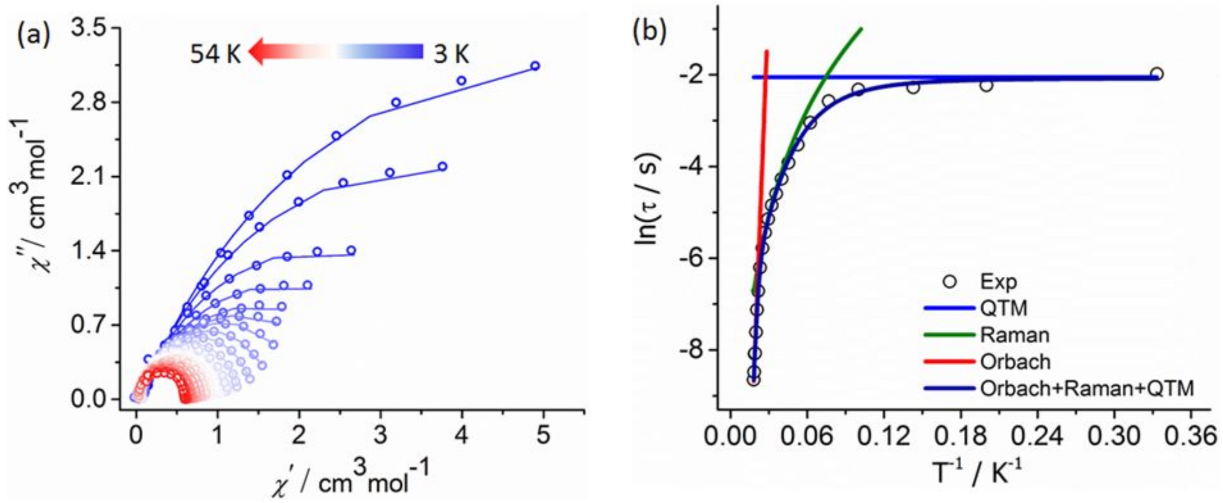

Figure S83. (a) Cole-Cole plot and (b) temperature-dependence of the relaxation time for [**Dy@3$_Y$**][$B(C_6F_5)_4$], where solid lines are fits to the data using equations 1 and 2. The parameters are stated in Table S10.

$$\chi'(\nu)=\chi_S+\frac{(\chi_T-\chi_S)[1+(2\Pi\nu\tau)^{(1-\alpha)}Sin(\frac{\alpha\Pi}{2})]}{1+2(2\Pi\nu\tau)^{(1-\alpha)}Sin\left(\frac{\alpha\Pi}{2}\right)+(2\Pi\nu\tau)^{2(1-\alpha)}} \text{.......................... (Equation 1)}$$

$$\chi''(\nu)=\frac{(\chi_T-\chi_S)(2\Pi\nu\tau)^{(1-\alpha)}Cos(\frac{\alpha\Pi}{2})}{1+2(2\Pi\nu\tau)^{(1-\alpha)}Sin\left(\frac{\alpha\Pi}{2}\right)+(2\Pi\nu\tau)^{2(1-\alpha)}} \text{.......................... (Equation 2)}$$

Table S8. Relaxation fitting parameters for $\mathbf{1_{Dy}}$ corresponding to Figure S76.

T / K	χ_S / cm^3 mol^{-1}	χ_T / cm^3 mol^{-1}	τ / s	α
1.9	0.34805	7.42342	0.01877	0.32328
2.2	0.31518	6.50588	0.01794	0.31884
2.5	0.28403	5.69681	0.0175	0.3177
3	0.24684	4.7751	0.01676	0.31516
3.5	0.22119	4.12698	0.01612	0.31238
4	0.20358	3.59431	0.01506	0.30537
4.5	0.19346	3.20162	0.01399	0.29469
5	0.18481	2.86672	0.01275	0.28212
6	0.17569	2.36093	0.00992	0.24369
7	0.16721	2.00717	0.00733	0.20298
8	0.15442	1.74148	0.00525	0.17087
9	0.14245	1.54092	0.00377	0.1476
10	0.12965	1.38281	0.00272	0.13248
11	0.12113	1.25493	0.00198	0.12039
12	0.11118	1.1544	0.00146	0.11674
13	0.10661	1.06518	0.00108	0.11077
14	0.1066	0.98878	7.90253E-4	0.10617
15	0.10623	0.92681	5.81677E-4	0.10154
16	0.10953	0.86841	4.10325E-4	0.09491
17	0.11282	0.81731	2.77349E-4	0.08478
18	0.12693	0.77143	1.83829E-4	0.06629
19	0.116	0.73048	1.10453E-4	0.06076
20	0.14945	0.69361	7.05284E-5	0.03839

Table S9. Relaxation fitting parameters for [$\mathbf{3_{Dy}}$][$B(C_6F_5)_4$] corresponding to Figure S81.

T / K	χ_S / cm^3 mol^{-1}	χ_T / cm^3 mol^{-1}	τ / s	α
16	1.13123	16.72355	0.09417	0.32396
19	1.14709	12.56862	0.03959	0.23208
22	1.08335	10.45158	0.02281	0.17744
25	1.04241	9.04531	0.01453	0.14882
28	0.96135	8.00449	0.00999	0.1243
31	0.90435	7.21175	0.00718	0.11023
34	0.8758	6.56651	0.00509	0.09037
37	0.85129	6.02556	0.00361	0.07256
40	0.76103	5.59211	0.00242	0.06984
43	0.76317	5.21935	0.00158	0.05118
46	0.6536	4.88657	9.21085E-4	0.051
48	0.67088	4.69649	6.29291E-4	0.0509
50	0.55964	4.51516	3.98497E-4	0.05079
52	0.60337	4.34043	2.56411E-4	0.03961
53	0.65244	4.27281	2.12629E-4	0.03788
54	0.64549	4.18016	1.64071E-4	0.04255
55	0.77449	4.13703	1.33392E-4	0.04617
56	0.70202	4.04057	1.0719E-4	0.0418
57	0.90831	3.96702	9.34707E-5	0.0034
58	0.6927	3.91448	6.32843E-5	0.04413

Table S10. Relaxation fitting parameters for [**Dy@3$_{Y}$**][B(C_6F_5)$_4$] corresponding to Figure S83.

T / K	χ_S / cm^3 mol^{-1}	χ_T / cm^3 mol^{-1}	τ / s	α
3	0.0496	7.05658	0.13799	0.29144
5	0.03507	4.46225	0.10755	0.28251
7	0.02558	3.54866	0.10262	0.3023
10	0.02721	2.94518	0.09783	0.30805
13	0.01863	2.55295	0.0759	0.28819
16	0.03894	2.08897	0.04761	0.22067
19	0.04209	1.71453	0.02936	0.16625
22	0.04053	1.46599	0.01986	0.12476
25	0.04929	1.27646	0.01401	0.09364
28	0.03983	1.14767	0.01012	0.10033
31	0.03534	1.01885	0.00784	0.05417
34	0.04489	0.92676	0.00583	0.03325
37	0.03906	0.85636	0.00434	0.04688
40	0.02901	0.79838	0.00309	0.05068
43	0.02925	0.74152	0.00202	0.03837
46	0.01234	0.69933	0.00121	0.06362
48	0.02197	0.66434	8.07005E-4	0.05967
50	0.0123	0.62816	4.92668E-4	0.06204
52	0.00012	0.62748	3.11695E-4	0.1009
53	0.08169	0.61706	3.08092E-4	0.0001
54	0.03057	0.59602	2.07251E-4	0.04724

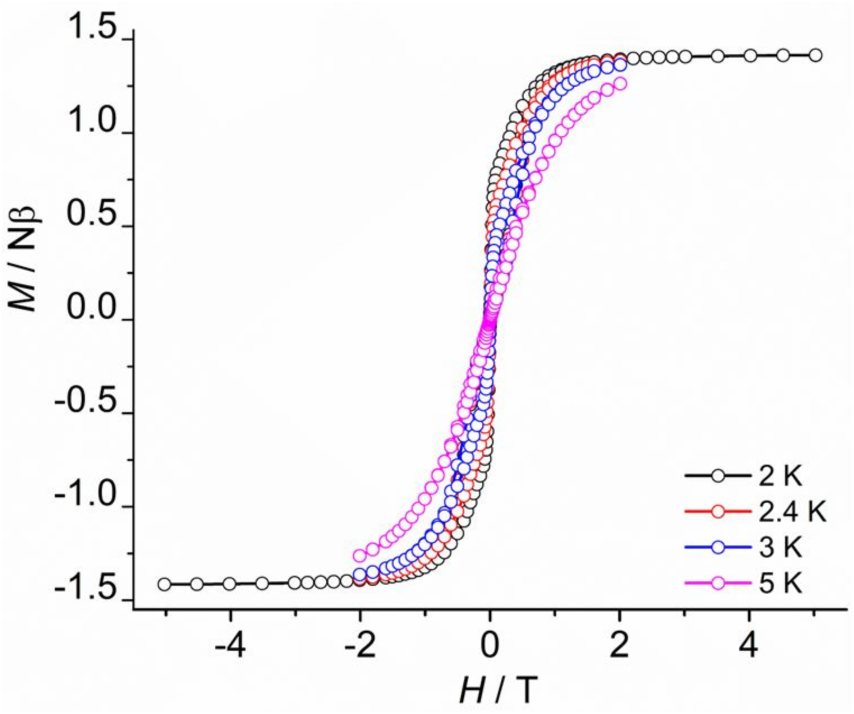

Figure S84. Magnetic Hysteresis loop for **Dy@3$_{Y}$**. The data were continuously collected at either 2 K (black circles), 2.4 K (red circles), 3 K (blue circles) and 5 K (pink circles) under a varying field sweep rate (5 mT s^{-1} |0-0.2| T, 10 mT s^{-1} |0.02-0.1| T, 50 mT s^{-1} |0.1-0.40| T, 100 mT s^{-1} |0.4-2.0| T, 200 mT s^{-1} |2.0-3.0| T, 500 mT s^{-1} |3.0-5.0| T). Solid lines are a guide to the eye.

Computational Details

All calculations were carried out on the coordinates obtained from the relevant crystal structure using the ORCA 5.0.2 software package.[8] The positions of hydrogen atoms were optimized at the DFT level using a pure GGA PBE exchange correlation functional, keeping the position of the other atoms constant.[9] To avoid the convergence problem, we replaced Dy^{3+} with Y^{3+} during the optimizations. The def2-TZVP basis sets with effective core potential (ECP) were used to treat the core electrons of yttrium throughout the DFT calculations.[10] In the multi-reference *ab initio* calculations, we used the DKH (Douglas-Kroll-Hess) Hamiltonian throughout to consider relativistic effects. The dysprosium centre was modelled with the SARC2-DKH-QZVP basis set, and all other atoms were treated with the DKH-def2-TZVP basis set in combination with the 'AutoAux' auxiliary basis set.[11] The active space CAS(7,9) was constructed from 9 electrons in 7 f-orbitals. In the configuration interaction procedure, 21 sextets, 128 quartets, and 130 doublets were computed for all the complexes. To consider the spin-orbit coupling, we also used the quasi-degenerate perturbation theory (QDPT) approach using SA-CASSCF wave functions.[12] The SINGLE_ANISO module as implemented in ORCA was used to compute the *g*-tensor and crystal field parameters of the low-lying excited states using previously calculated spin-orbit states.[13] Additionally, the POLY_ANISO module[14] was used to simulate the magnetic data and calculate the exchange coupled states for $\mathbf{3_{Dy}}$.

Table S11. Kramers doublet (KD) energies, g-tensors, angle between the anisotropic axis of the excited states and ground state, and wavefunction compositions for **1**$_{\mathbf{Dy}}$ (disordered part A).

<table>
<tr><th>KD</th><th>E / cm^{-1}</th><th>g_x</th><th>g_y</th><th>g_z</th><th>Angle/°</th><th>Wave function composition</th></tr>
<tr><td>1</td><td>0.000</td><td>0.006</td><td>0.016</td><td>19.70</td><td>-</td><td>97.86%|±15/2>+0.98%|±11/2>0.58%|±9/2>+0.11%|±7/2>++0.38%|±5/2></td></tr>
<tr><td>2</td><td>144.406</td><td>0.008</td><td>0.257</td><td>18.81</td><td>47.94</td><td>1.11%|±15/2>+17.42%|±13/2>+30.46%|±11/2>+23.01%|±9/2>+21.10%|±7/2>+3.47%|±5/2>+2.16%|±3/2>+1.24%|±1/2></td></tr>
<tr><td>3</td><td>175.159</td><td>1.086</td><td>2.659</td><td>15.53</td><td>131.95</td><td>0.15%|±15/2>+27.89%|±13/2>+6.06%|±11/2>+29.44%|±9/2>+11.80%|±7/2>+12.49%|±5/2>+7.08%|±3/2>+5.05%|±1/2></td></tr>
<tr><td>4</td><td>211.339</td><td>0.346</td><td>3.373</td><td>11.56</td><td>20.50</td><td>0.37%|±15/2>+34.36%|±13/2>+15.53%|±11/2>+10.56%|±9/2>+19.64%|±7/2>+13.64%|±5/2>+2.16%|±3/2>+3.72%|±1/2></td></tr>
<tr><td>5</td><td>279.080</td><td>9.978</td><td>5.941</td><td>1.85</td><td>106.18</td><td>0.13%|±15/2>+17.80%|±13/2>+21.11%|±11/2>+10.48%|±9/2>+19.95%|±7/2>+14.55%|±5/2>+13.62%|±3/2>+2.32%|±1/2></td></tr>
<tr><td>6</td><td>327.388</td><td>2.828</td><td>3.490</td><td>14.73</td><td>118.35</td><td>0.30%|±15/2>+1.84%|±13/2>+21.75%|±11/2>+15.58%|±9/2>+12.38%|±7/2>+30.75%|±5/2>+13.97%|±3/2>+3.39%|±1/2></td></tr>
<tr><td>7</td><td>401.653</td><td>0.243</td><td>1.772</td><td>15.28</td><td>83.02</td><td>0.03%|±15/2>+0.14%|±13/2>+3.45%|±11/2>+9.37%|±9/2>+9.15%|±7/2>+10.25%|±5/2>+35.36%|±3/2>+31.94%|±1/2></td></tr>
<tr><td>8</td><td>445.754</td><td>0.609</td><td>2.238</td><td>17.85</td><td>81.74</td><td>0.024%|±15/2>+0.49%|±13/2>+0.61%|±11/2>+0.94%|±9/2>+3.83%|±7/2>+14.18%|±5/2>+25.58%|±3/2>+52.31%|±1/2></td></tr>
</table>

Table S12. Kramers doublet (KD) energies, *g*-tensors, angle between the anisotropic axis of the excited states and ground state, and wavefunction compositions for $\mathbf{1}_{\mathbf{Dy}}$ (disordered part B).

<table>
<tr><th>KD</th><th>E / cm^{−1}</th><th>g_x</th><th>g_y</th><th>g_z</th><th>Angle/°</th><th>Wave function composition</th></tr>
<tr><td>1</td><td>0.000</td><td>0.018</td><td>0.035</td><td>19.62</td><td>-</td><td>97.49%|±15/2>+0.01%|±13/2>+0.34%|±11/2>0.70%|±9/2>+1.05%|±7/2>++0.33%|±5/2></td></tr>
<tr><td>2</td><td>157.061</td><td>0.640</td><td>1.858</td><td>16.37</td><td>38.45</td><td>0.58%|±15/2>+35.27%|±13/2>+13.78%|±11/2>+28.17%|±9/2>+9.83%|±7/2>+9.40%|±5/2>+2.01%|±3/2>+0.92%|±1/2></td></tr>
<tr><td>3</td><td>193.899</td><td>0.520</td><td>1.833</td><td>14.93</td><td>133.19</td><td>1.45%|±15/2>+8.30%|±13/2>+31.79%|±11/2>+8.28%|±9/2>+29.97%|±7/2>+6.92%|±5/2>+8.61%|±3/2>+4.66%|±1/2></td></tr>
<tr><td>4</td><td>259.463</td><td>1.294</td><td>2.631</td><td>13.68</td><td>23.88</td><td>0.23%|±15/2>+43.04%|±13/2>+5.31%|±11/2>+26.77%|±9/2>+3.66%|±7/2>+17.14%|±5/2>+1.16%|±3/2>+2.68%|±1/2></td></tr>
<tr><td>5</td><td>324.778</td><td>9.427</td><td>6.120</td><td>1.75</td><td>102.64</td><td>10.19%|±13/2>+29.86%|±11/2>+7.93%|±9/2>+27.62%|±7/2>+5.44%|±5/2>+15.52%|±3/2>+3.37%|±1/2></td></tr>
<tr><td>6</td><td>383.995</td><td>8.189</td><td>6.837</td><td>0.082</td><td>75.90</td><td>0.12%|±15/2>+1.83%|±13/2>+13.90%|±11/2>+19.60%|±9/2>+9.30%|±7/2>+42.19%|±5/2>+0.58%|±3/2>+12.43%|±1/2></td></tr>
<tr><td>7</td><td>419.989</td><td>2.533</td><td>6.041</td><td>12.34</td><td>95.97</td><td>0.05%|±15/2>0.66%|±13/2>+3.06%|±11/2>+6.27%|±9/2>+14.68%|±7/2>+8.26%|±5/2>+49.20%|±3/2>+17.79%|±1/2></td></tr>
<tr><td>8</td><td>476.610</td><td>0.350</td><td>0.912</td><td>19.04</td><td>85.90</td><td>0.03%|±15/2>+0.65%|±13/2>+1.94%|±11/2>+2.25%|±9/2>+3.85%|±7/2>+10.28%|±5/2>+22.86%|±3/2>+58.10%|±1/2></td></tr>
</table>

Table S13. Kramers doublet (KD) energies, *g*-tensors, angle between the anisotropic axis of the excited states and ground state, and wavefunction compositions for **2**$_{\mathbf{Dy}}$.

<table>
<tr><th>KD</th><th>E / cm^{-1}</th><th>g_x</th><th>g_y</th><th>g_z</th><th>Angle/°</th><th>Wave function composition</th></tr>
<tr><td>1</td><td>0.000</td><td>0.019</td><td>0.035</td><td>19.59</td><td>-</td><td>95.15%|±15/2>+4.53%|±11/2>+0.24%|±7/2></td></tr>
<tr><td>2</td><td>227.758</td><td>0.738</td><td>1.546</td><td>15.64</td><td>4.72</td><td>80.08%|±13/2>+0.30%|±11/2>+14.22%|±9/2>+0.39%|±7/2>+2.96%|±5/2>+0.67%|±3/2>+1.3%|±1/2></td></tr>
<tr><td>3</td><td>334.546</td><td>4.143</td><td>5.279</td><td>10.88</td><td>96.89</td><td>2.26%|±15/2>+6.25%|±13/2>+26.20%|±11/2>+1.51%|±9/2>+23.59%|±7/2>+8.56%|±5/2>+17.82%|±3/2>+13.77%|±1/2></td></tr>
<tr><td>4</td><td>416.997</td><td>0.574</td><td>2.770</td><td>8.49</td><td>88.62</td><td>1.19%|±15/2>+6.64%|±13/2>+40.94%|±11/2>+12.71%|±9/2>+3.16%|±7/2>+19.59%|±5/2>+3.11%|±3/2>+11.90%|±1/2></td></tr>
<tr><td>5</td><td>518.511</td><td>1.215</td><td>1.45</td><td>11.56</td><td>90.71</td><td>0.46%|±15/2>+5.27%|±13/2>+17.84%|±11/2>+37.65%|±9/2>+9.79%|±7/2>+4.84%|±5/2>+19.30%|±3/2>+4.80%|±1/2></td></tr>
<tr><td>6</td><td>627.959</td><td>0.226</td><td>0.238</td><td>14.39</td><td>91.90</td><td>0.13%|±15/2>+1.39%|±13/2>+8.04%|±11/2>+24.26%|±9/2>+34.20%|±7/2>+12.25%|±5/2>+1.04%|±3/2>+18.64%|±1/2></td></tr>
<tr><td>7</td><td>776.024</td><td>0.014</td><td>0.016</td><td>17.12</td><td>90.97</td><td>0.02%|±15/2>+0.28%|±13/2>+1.84%|±11/2>+8.26%|±9/2>+23.28%|±7/2>+36.16%|±5/2>+26.06%|±3/2>+4.06%|±1/2></td></tr>
<tr><td>8</td><td>1060.200</td><td>0.000</td><td>0.001</td><td>19.78</td><td>89.77</td><td>0.26%|±11/2>+1.33%|±9/2>+5.32%|±7/2>+15.60%|±5/2>+31.92%|±3/2>+45.49%|±1/2></td></tr>
</table>

Table S14. Kramers doublet (KD) energies, *g*-tensors, angle between the anisotropic axis of the excited states and ground state, and wavefunction compositions for $\mathbf{3_{Dy}}$ (Dy1).

KD	*E* / cm^{-1}	g_x	g_y	g_z	Angle/°	Wave function composition
1	0.000	0.001	0.001	19.78	-	97.58%|±15/2>+2.36%|±11/2>
2	280.832	0.028	0.035	16.89	1.97	95.68%|±13/2>+0.16%|±11/2>+3.92%|±9/2>+0.16%|±5/2>
3	489.948	0.533	0.717	13.97	3.85	2.31%|±15/2>+0.06%|±13/2>+87.60%|±11/2>+1.03%|±9/2>+7.29%|±7/2>+0.29%|±5/2>+1.02%|±3/2>+0.36%|±1/2>
4	611.565	8.089	7.018	4.290	92.54	0.02%|±15/2>+3.23%|±13/2>+1.77%|±11/2>+55.69%|±9/2>+3.58%|±7/2>+19.51%|±5/2>+6.45%|±3/2>+9.70%|±1/2>
5	684.814	1.206	3.919	9.75	92.90	0.05%|±15/2>+0.79%|±13/2>+5.42%|±11/2>+24.35%|±9/2>+28.58%|±7/2>+2.06%|±5/2>+24.95%|±3/2>+13.75%|±1/2>
6	786.482	0.856	0.940	13.43	93.80	0.01%|±15/2>+0.17%|±13/2>+2.29%|±11/2>+11.36%|±9/2>+41.03%|±7/2>+24.89%|±5/2>+0.45%|±3/2>+19.76%|±1/2>
7	925.131	0.176	0.274	16.59	91.87	0.32%|±11/2>+3.21%|±9/2>+16.52%|±7/2>+40.15%|±5/2>+34.26%|±3/2>+5.48%|±1/2>
8	1162.142	0.001	0.003	19.63	89.42	0.40%|±9/2>+2.90%|±7/2>+12.90%|±5/2>+32.83%|±3/2>+50.89%|±1/2>

Table S15. Kramers doublet (KD) energies, *g*-tensors, angle between the anisotropic axis of the excited states and ground state, and wavefunction compositions for $\mathbf{3_{Dy}}$ (Dy2).

<table>
<tr><th>KD</th><th>E / cm^{−1}</th><th>g_x</th><th>g_y</th><th>g_z</th><th>Angle/°</th><th>Wave-function composition</th></tr>
<tr><td>1</td><td>0.000</td><td>0.001</td><td>0.001</td><td>19.79</td><td></td><td>97.78%|±15/2>+2.16%|±11/2></td></tr>
<tr><td>2</td><td>303.549</td><td>0.030</td><td>0.038</td><td>16.87</td><td>1.02</td><td>95.63%|±13/2>+4.06%|±9/2>+0.22%|±5/2></td></tr>
<tr><td>3</td><td>517.058</td><td>0.493</td><td>0.630</td><td>13.97</td><td>1.76</td><td>2.15%|±15/2>+0.09%|±13/2>+88.65%|±11/2>+0.14%|±9/2>+7.49%|±7/2>+0.09%|±5/2>+1.07%|±3/2>+0.28%|±1/2></td></tr>
<tr><td>4</td><td>642.570</td><td>3.817</td><td>5.526</td><td>8.97</td><td>90.32</td><td>3.59%|±13/2>+1.22%|±11/2>+63.18%|±9/2>+1.56%|±7/2>+18.71%|±5/2>+4.02%|±3/2>+7.68%|±1/2></td></tr>
<tr><td>5</td><td>717.098</td><td>2.458</td><td>4.745</td><td>9.50</td><td>89.53</td><td>0.03%|±15/2>+0.51%|±13/2>+5.62%|±11/2>+18.09%|±9/2>+33.62%|±7/2>+1.53%|±5/2>+26.83%|±3/2>+13.72%|±1/2></td></tr>
<tr><td>6</td><td>813.442</td><td>0.383</td><td>0.429</td><td>13.46</td><td>90.00</td><td>0.14%|±13/2>+1.97%|±11/2>+11.10%|±9/2>+38.28%|±7/2>+27.24%|±5/2>+0.28%|±3/2>+20.95%|±1/2></td></tr>
<tr><td>7</td><td>957.061</td><td>0.128</td><td>0.201</td><td>16.56</td><td>90.15</td><td>0.27%|±11/2>+3.07%|±9/2>+16.25%|±7/2>+39.56%|±5/2>+34.96%|±3/2>+5.84%|±1/2></td></tr>
<tr><td>8</td><td>1205.267</td><td>0.002</td><td>0.002</td><td>19.61</td><td>90.23</td><td>0.33%|±9/2>+2.73%|±7/2>+12.61%|±5/2>+32.80%|±3/2>+51.47%|±1/2></td></tr>
</table>

Table S16. Magnitudes of transition magnetic moment matrix elements (in Bohr magneton) calculated for $\mathbf{1}_{\mathbf{Dy}}$ (disordered part A).

Climbing Transition			Crossing Transition		
Initial KD	Final KD	Magnitude	Initial KD	Final KD	Magnitude
1	2	0.85976	1	1	0.00384
1	3	1.08006	1	2	0.05051
1	4	1.18761	1	3	0.19336
1	5	0.64069	1	4	0.21211
1	6	0.27902	1	5	0.09931
1	7	0.09616	1	6	0.06397
1	8	0.09442	1	7	0.02362
2	3	1.14434	1	8	0.01635
2	4	1.37137	2	2	0.05885
2	5	1.22868	2	3	0.35815
2	6	0.31878	2	4	0.32780
2	7	0.30040	2	5	0.30710
2	8	0.41552	2	6	0.45040
3	4	2.35924	2	7	0.14021
3	5	0.83116	2	8	0.06511
3	6	0.87643	3	3	0.82541
3	7	0.76902	3	4	1.10132
3	8	0.35077	3	5	0.37588
4	5	2.65248	3	6	0.29159
4	6	0.89464	3	7	0.15534
4	7	0.50930	3	8	0.15980
4	8	0.30185	4	4	0.64686
5	6	2.56064	4	5	0.94461
5	7	1.47814	4	6	0.42393
5	8	0.53273	4	7	0.54404
6	7	1.66394	4	8	0.23764
6	8	0.52748	5	5	1.38609
7	8	2.10247	5	6	1.43245
			5	7	1.01137
			5	8	0.41349
			6	6	1.64378
			6	7	1.33427
			6	8	0.94489
			7	7	2.38162
			7	8	1.07478
			8	8	1.07022

Table S17. Magnitudes of transition magnetic moment matrix elements (in Bohr magneton) calculated for **1**$_{Dy}$ (disordered part B).

Climbing Transition			Crossing Transition		
Initial KD	Final KD	Magnitude	Initial KD	Final KD	Magnitude
1	2	1.36896	1	1	0.00900
1	3	0.78462	1	2	0.10251
1	4	1.08185	1	3	0.14522
1	5	0.50970	1	4	0.07142
1	6	0.20397	1	5	0.12695
1	7	0.11819	1	6	0.10384
1	8	0.09279	1	7	0.06624
2	3	2.02089	1	8	0.02274
2	4	1.24773	2	2	0.50502
2	5	1.00135	2	3	0.66574
2	6	0.49912	2	4	0.27192
2	7	0.35713	2	5	0.15485
2	8	0.32806	2	6	0.19467
3	4	2.21903	2	7	0.23700
3	5	0.90492	2	8	0.06389
3	6	0.98232	3	3	0.58716
3	7	0.76743	3	4	0.38003
3	8	0.18377	3	5	0.52825
4	5	2.76689	3	6	0.37493
4	6	0.38075	3	7	0.26318
4	7	0.55001	3	8	0.19120
4	8	0.28876	4	4	0.69935
5	6	2.89273	4	5	0.71128
5	7	0.86030	4	6	0.34727
5	8	0.53065	4	7	0.21143
6	7	2.95520	4	8	0.14072
6	8	0.81990	5	5	1.40113
7	8	1.45253	5	6	0.77423
			5	7	0.55878
			5	8	0.35670
			6	6	1.35200
			6	7	1.44321
			6	8	0.32211
			7	7	2.72315
			7	8	1.52033
			8	8	1.09072

Table S18. Magnitudes of transition magnetic moment matrix elements (in Bohr magneton) calculated for $\mathbf{2_{Dy}}$.

Climbing Transition			Crossing Transition		
Initial KD	Final KD	Magnitude	Initial KD	Final KD	Magnitude
1	2	1.80414	1	1	0.00906
1	3	0.29910	1	2	0.03744
1	4	0.13822	1	3	0.24181
1	5	0.10625	1	4	0.16670
1	6	0.06424	1	5	0.05274
1	7	0.03625	1	6	0.04699
1	8	0.00646	1	7	0.02104
2	3	2.25862	1	8	0.00953
2	4	1.19361	2	2	0.38234
2	5	0.13164	2	3	0.60668
2	6	0.14649	2	4	0.38015
2	7	0.09093	2	5	0.34759
2	8	0.01946	2	6	0.09337
3	4	1.87701	2	7	0.06212
3	5	0.32128	2	8	0.02640
3	6	0.12022	3	3	2.47196
3	7	0.09297	3	4	1.98577
3	8	0.02757	3	5	0.23957
4	5	2.16595	3	6	0.14753
4	6	0.25180	3	7	0.07805
4	7	0.06303	3	8	0.03235
4	8	0.03432	4	4	1.53666
5	6	2.10644	4	5	1.62136
5	7	0.19208	4	6	0.26232
5	8	0.06548	4	7	0.06551
6	7	2.29567	4	8	0.03862
6	8	0.04517	5	5	2.18385
7	8	0.04221	5	6	1.79659
			5	7	0.19063
			5	8	0.07244
			6	6	1.11574
			6	7	0.51730
			6	8	0.19369
			7	7	0.14619
			7	8	1.70311
			8	8	0.03749

Table S19. Magnitudes of transition magnetic moment matrix elements (in Bohr magneton) calculated for $\mathbf{3_{Dy}}$ (Dy1).

Climbing Transition			Crossing Transition		
Initial KD	Final KD	Magnitude	Initial KD	Final KD	Magnitude
1	2	1.78198	1	1	0.00041
1	3	0.23725	1	2	0.00115
1	4	0.11606	1	3	0.00465
1	5	0.01897	1	4	0.01371
1	6	0.02367	1	5	0.07344
1	7	0.01877	1	6	0.04448
1	8	0.00625	1	7	0.01547
2	3	2.36216	1	8	0.00382
2	4	0.28934	2	2	0.00106
2	5	0.18303	2	3	0.02369
2	6	0.09806	2	4	0.13793
2	7	0.04082	2	5	0.07705
2	8	0.02300	2	6	0.04541
3	4	2.62646	2	7	0.05693
3	5	0.45748	2	8	0.01583
3	6	0.17193	3	3	0.20899
3	7	0.06487	3	4	0.27849
3	8	0.02692	3	5	0.94852
4	5	2.54237	3	6	0.32923
4	6	0.74255	3	7	0.08246
4	7	0.14561	3	8	0.02067
4	8	0.07227	4	4	1.88656
5	6	2.04732	4	5	1.45218
5	7	0.49123	4	6	0.39499
5	8	0.08433	4	7	0.25896
6	7	2.27296	4	8	0.03132
6	8	0.14162	5	5	2.19692
7	8	0.27711	5	6	1.82299
			5	7	0.21408
			5	8	0.13033
			6	6	1.82179
			6	7	0.86920
			6	8	0.29572
			7	7	0.91163
			7	8	1.72550
			8	8	0.05022

Table S20. Magnitudes of transition magnetic moment matrix elements (in Bohr magneton) calculated for $\mathbf{3}_{\mathbf{Dy}}$ (Dy2).

Climbing Transition			Crossing Transition		
Initial KD	Final KD	Magnitude	Initial KD	Final KD	Magnitude
1	2	1.78247	1	1	0.00042
1	3	0.17897	1	2	0.00108
1	4	0.10682	1	3	0.00425
1	5	0.00770	1	4	0.00643
1	6	0.00699	1	5	0.05649
1	7	0.01932	1	6	0.04118
1	8	0.00462	1	7	0.00609
2	3	2.37860	1	8	0.00391
2	4	0.23119	2	2	0.01152
2	5	0.15828	2	3	0.02058
2	6	0.09303	2	4	0.10603
2	7	0.02101	2	5	0.04705
2	8	0.01846	2	6	0.00950
3	4	2.68963	2	7	0.05514
3	5	0.27212	2	8	0.01702
3	6	0.06929	3	3	0.18755
3	7	0.08118	3	4	0.23131
3	8	0.02101	3	5	0.76121
4	5	2.58457	3	6	0.30932
4	6	0.88519	3	7	0.02912
4	7	0.06796	3	8	0.02764
4	8	0.05483	4	4	1.55768
5	6	1.04419	4	5	0.90243
5	7	0.48429	4	6	0.16453
5	8	0.10270	4	7	0.29346
6	7	1.68636	4	8	0.05758
6	8	0.19741	5	5	2.42832
7	8	1.44329	5	6	1.83005
			5	7	0.15960
			5	8	0.11142
			6	6	2.54862
			6	7	1.10858
			6	8	0.20075
			7	7	2.68652
			7	8	1.01983
			8	8	0.11629

Table S21. SINGLE_ANISO computed crystal-field parameters for $\mathbf{1_{Dy}}$, $\mathbf{2_{Dy}}$ and $\mathbf{3_{Dy}}$.

k	*q*	$\mathbf{1_{Dy}}$		$\mathbf{2_{Dy}}$	$\mathbf{3_{Dy}}$	
		**Dy (part A)**	**Dy (part B)**		**Dy1**	**Dy2**
	-2	0.7336E+00	-0.9795E+00	-0.3157E+00	0.3012E+00	-0.2913E-01
	-1	0.1272E+01	0.9632E+00	0.2463E+00	-0.8643E-01	0.2232E+00
2	0	-0.1827E+01	-0.1972E+01	-0.4120E+01	-0.5441E+01	-0.5637E+01
	1	-0.2668E+00	0.2168E+00	-0.1776E+00	0.5073E+00	0.5083E-01
	2	0.1349E+01	0.1644E+01	0.6031E+01	0.4307E+01	0.4343E+01
	-4	-0.1070E-01	-0.1073E-02	-0.3457E-03	-0.1168E-03	-0.2438E-03
	-3	0.4035E-01	0.9070E-02	-0.2007E-02	-0.2198E-02	0.1671E-02
	-2	-0.3167E-02	-0.1072E-02	0.1538E-02	-0.1396E-02	-0.1172E-02
	-1	-0.4519E-02	-0.5940E-03	-0.1628E-02	0.1087E-03	-0.6039E-03
4	0	-0.8668E-03	-0.1626E-02	-0.1771E-02	-0.1633E-02	-0.1776E-02
	1	-0.6215E-02	-0.2025E-02	-0.9740E-03	0.3814E-03	-0.4393E-03
	2	0.1519E-03	0.5016E-02	-0.4606E-02	-0.3263E-02	-0.3403E-02
	3	0.1016E-02	0.2499E-01	-0.7595E-03	-0.6553E-02	0.3456E-02
	4	0.1277E-02	-0.1497E-01	0.4382E-03	-0.1143E-02	-0.8674E-02
	-6	-0.3943E-04	-0.6529E-04	-0.9124E-04	0.4811E-04	0.7244E-04
	-5	-0.7063E-03	-0.2659E-03	0.5186E-04	-0.4914E-04	-0.5970E-05
	-4	0.3753E-04	-0.8938E-05	-0.9027E-05	-0.9256E-06	0.9449E-05
	-3	-0.4200E-04	-0.1225E-03	-0.1356E-04	0.1404E-04	0.1032E-04
	-2	-0.1046E-03	0.9575E-04	0.1196E-03	-0.1198E-03	-0.1274E-03
	-1	-0.6023E-04	-0.1286E-03	-0.1828E-04	0.7583E-05	-0.1753E-04
6	0	-0.4214E-04	-0.4249E-04	-0.1689E-04	-0.8948E-05	-0.1391E-04
	1	0.1531E-03	0.4474E-03	0.2387E-04	-0.5946E-04	0.3379E-05
	2	-0.1561E-03	-0.1385E-03	0.1976E-03	0.2266E-03	0.2219E-03
	3	0.3688E-04	0.7789E-04	-0.4726E-04	0.3171E-04	0.6473E-05
	4	0.1592E-04	-0.1482E-03	-0.2781E-04	-0.2738E-04	-0.5818E-04
	5	0.7741E-03	-0.9377E-03	-0.4895E-04	-0.7308E-04	0.1251E-04
	6	0.1854E-03	-0.7695E-05	0.1479E-03	0.5291E-04	0.4049E-04

The following Hamiltonian was used to calculate the crystal field parameters:

$$\hat{H}_{\mathrm{CF}} = \sum\sum_{k=-q}^{+q} B_k^q \hat{O}_k^q$$

(Where $\hat{O}_k^q$ is the extended Stevens operator; B_k^q is the crystal field parameter; *k* is the rank of the irreducible tensor operator (ITO) (*k* = 2, 4, 6); *q* is the component of the ITO (*q* = -k, -k+1, ... 0, 1, ... k.)

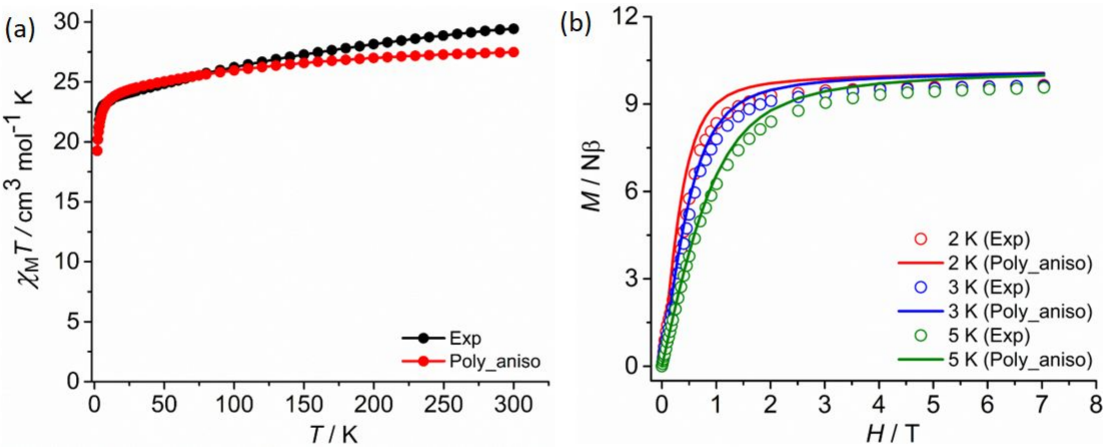

Figure S85. Experimental and simulated magnetic susceptibility and isothermal magnetization data for $\mathbf{3_{Dy}}$.

Table S22. Magnetic coupling parameters from the simulation of the susceptibility and magnetization data using a Lines model and an intermolecular interaction of –0.003 cm^{-1} for $\mathbf{3_{Dy}}$.

J_{ex} / cm^{-1}	J_{dip} / cm^{-1}	J_{tot} / cm^{-1}
-0.70	+0.04	-0.66

Table S23. Energies and the corresponding tunnelling gaps and g_Z values of the low-lying exchange doublet states obtained from the simulation of susceptibility and magnetization data for $\mathbf{3_{Dy}}$.

**Energy / cm^{-1}**	**D_{tun}**	**g_z**
0.00000<br>0.00000	2.08×10^{-7}	25.01
1.7330<br>1.7330	1.17×10^{-7}	30.58
280.872<br>280.872	5.06×10^{-6}	22.94
282.528<br>282.528	2.77×10^{-6}	28.79
303.717<br>303.717	9.39×10^{-6}	23.36
304.147<br>304.147	8.26×10^{-6}	28.39
584.578<br>584.578	1.31×10^{-4}	21.08
585.944<br>585.944	7.33×10^{-5}	26.44

References

1. Cendrowski-Guillaume, S. M.; Le Gland, G.; Nierlich, M.; Ephritikhine, M. Lanthanide Borohydrides as Precursors to Organometallic Compounds. Mono(cyclooctatetraenyl) Neodymium Complexes. *Organometallics*, **2000**, *19*, 5654–5660.
2. Venier, C. G.; Casserly, E. W.; Di-tert-butylcyclopentadiene and tri-tert-butylcyclopentadiene. *J. Am. Chem. Soc.* **2002**, *112*, 2808–2809.
3. Du Plooy, K. E.; Du Toit, J.; Levendis D. C.; Coville, N. J. Multiply substituted cyclopentadienyl metal complexes: I. Solid-state and Solution Conformational Studies on (η^5-C_5Me_4R)Fe(CO)LI (R = H, ^{t}Bu). *J. Organomet. Chem.* 1996, **508**, 231–242.
4. J. Sandström, *Dynamic NMR Spectroscopy*, Academic Press, Cambridge, 1982.
5. Dolomanov, O. V.; Bourhis, L. J.; Gildea, R. J.; Howard, J. A. K.; Puschmann H. OLEX2: A Complete Structure Solution, Refinement and Analysis Program. *J. Appl. Cryst.* **2009**, *42*, 339–341.
6. Sheldrick, G. M. Crystal Structure Refinement with SHELXL. *Acta Cryst.* **2015**, *71*, 3–8.
7. D. Gatteschi, R. Sessoli, J. Villain, Molecular Nanomagnets (Oxford Univ. Press, 2006).
8. Neese, F.; Wennmohs, F.; Becker, U.; Riplinger, C. The ORCA Quantum Chemistry Program Package. *J. Chem. Phys.* **2020**, *152*, 224108.
9. (a) Perdew, J. P.; Burke, K; Ernzerhof, M. Generalized Gradient Approximation Made Simple *Phys. Rev. Lett.* **1997**, *78*, 1396-1396; (b) Perdew, J. P.; Burke, K; Ernzerhof, M. Generalized Gradient Approximation Made Simple. *Phys. Rev. Lett.* **1996**, *77*, 3865-3868.
10. (a) Schäfer, A.; Horn H.; Ahlrichs, R. Fully Optimized Contracted Gaussian Basis Sets for Atoms Li to Kr *J. Chem. Phys.* **1992**, *97*, 2571-2577; (b) Weigend, F.; Ahlrichs, R. Balanced Basis Sets of Split Valence, Triple Zeta Valence and Quadruple Zeta Valence Quality for H to Rn: Design and Assessment of Accuracy. *Phys. Chem. Chem. Phys.* **2005**, *7*, 3297-3305.
11. (a) Aravena, D.; Neese, F.; Pantazis, D. A. Development of All-Electron Basis Sets. *J. Chem. Theory Comput.* **2016**, *12*, 1148-1156. (b) Chmela, J.; Harding, M. E. Optimized Auxiliary Basis Sets for Density Fitted Post-Hartree-Fock Calculations of Lanthanide Containing Molecules. *Mol. Phys.* **2018**, *116*, 1523-1538.
12. Ganyushin, D.; Neese, F. First-principles Calculations of Zero-field Splitting Parameters. *J. Chem. Phys.* **2006**, *125*, 024103.
13. Chibotaru, L. F.; Ungur, L. Ab Initio Calculation of Anisotropic Magnetic Properties of Complexes. I. Unique Definition of Pseudospin Hamiltonians and their Derivation. *J. Chem. Phys.* **2012**, *137*, 064112.
14. Chibotaru, L. F.; Ungur, L.; Aronica, C.; Elmoll, H.; Pilet, G.; Luneau, D. Structure, Magnetism, and Theoretical Study of a Mixed-Valence $Co^{II}_3Co^{III}_4$ Heptanuclear Wheel: Lack of SMM Behavior Despite Negative Magnetic Anisotropy. *J. Am. Chem. Soc.* **2008**, *130*, 12445–12455.
